# Organic Redox Species in Aqueous Flow Batteries: Redox Potentials, Chemical Stability and Solubility

**DOI:** 10.1038/srep39101

**Published:** 2016-12-14

**Authors:** Kristina Wedege, Emil Dražević, Denes Konya, Anders Bentien

**Affiliations:** 1 Department of Engineering - Aarhus University, Hangøvej, 2,8200 Aarhus N, Denmark; 2 Research Centre for Natural Sciences, Magyar tudósok körútja 2, 1117 Budapest, Hungary

## Abstract

Organic molecules are currently investigated as redox species for aqueous low-cost redox flow batteries (RFBs). The envisioned features of using organic redox species are low cost and increased flexibility with respect to tailoring redox potential and solubility from molecular engineering of side groups on the organic redox-active species. In this paper 33, mainly quinone-based, compounds are studied experimentially in terms of pH dependent redox potential, solubility and stability, combined with single cell battery RFB tests on selected redox pairs. Data shows that both the solubility and redox potential are determined by the position of the side groups and only to a small extent by the number of side groups. Additionally, the chemical stability and possible degradation mechanisms leading to capacity loss over time are discussed. The main challenge for the development of all-organic RFBs is to identify a redox pair for the positive side with sufficiently high stability and redox potential that enables battery cell potentials above 1 V.

The widespread interest in replacing fossil fuels with renewable energy sources is a challenging task motivated by a need for energy independence and carbon footprint reduction. The cost of wind and photovoltaics electricity is still decreasing and will be integrated even more in a future fully renewable-based utility grid. However, this integration is faced with major challenges with respect to stability and security of supply because of the mismatch between electricity production and demand^1^. This can to some extent be solved by expansion of the transmission grid, intelligent demand-response schemes and storage/use of surplus electricity with heat pumps in reservoirs or pumped hydro^2^,^3^,^4^,^5^. Nonetheless, there will be a need for local/regional electricity storage^5^. Here batteries, which already are applied in the utility grid, are an obvious solution. However, a broader market penetration is critically dependent on the cost, which can be quantified by the *levelized cost of electricity storage (LCES*)^6^,^7^. In the simplest form it is given by 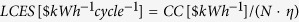, where *LCES* expresses the cost of storing and discharging one kWh in a cycle, *CC* are the capital costs per kWh, *N* the number of cycles during lifetime and *η* is the cycle efficiency^8^. Here capital cost in the range $120-$150 kWh^−1^ and a LCES of less than ¢10 kWh^−1^ cycle^−1^ is generally considered a breakthrough in energy storage^9^,^10^.

Recently, organic aqueous flow batteries have been proposed as a low-cost alternative to the present metal-based RFB technology^11^,^12^,^13^,^14^. Additionally, it is envisioned that the redox potential and solubility can be tailored by introduction of specific side groups on the organic redox-active species. The maximum allowable cost of organic redox species was studied in a scenario where a system capital cost for an aqueous RFB of $120 kWh^−1^ was targeted^9^. With moderately optimistic assumptions of $300 kW^−1^ for balance-of-plant/profit and flow cell cost normalised to internal resistance of 5 $ mΩ, it was shown that cell potentials of 1 V and 1.5 V result in a maximum acceptable costs of redox species of $4 kg^−1^ ($15 kAh^−1^) and $7 kg^−1^ ($26 kAh^−1^) respectively^9^. An interesting point here is that the redox species maximum allowable cost scales with the cell potential with a power greater than one. This is because not only the energy density increases with cell potential, but also cycle efficiency for a given internal flow cell resistance. Cost limits in the range $4 kg^−1^ ($15 kAh^−1^) and $7 kg^−1^ ($26 kAh^−1^) for organic redox species are not unrealistic. The base chemical cost for e.g. benzene is around $1.2 kg^−1^ while the cost of unsubstituted anthraquinone is $10–15 kAh^−1^ (see Supplementary, S9).

Figure 1 outlines an all-organic RFB^13^. It is based on 4,5-dihydroxy-1,3-benzenedisulfonate (HQ(1,2)DS) on the positive side (container A) and anthraquinone-2-sulfonate (AQ(2)S) on the negative (container B). During charging/discharging, two electrons are exchanged through an external circuit as HQ(1,2)DS and AQ(2)S is reduced and oxidized and two protons are exchanged through a Nafion membrane. It shows high coulombic efficiency, however the cell voltage is merely 0.7 V and significant capacity loss was observed due to chemical instability of the sulfonated hydroquinone^13^,^15^. Two other all-organic, radical based RFBs were recently demonstrated with a cell potential of 1.25 V^14^,^16^. They employ the organic radical TEMPOL (4-hydroxy-TEMPO) on the positive side and viologen on the negative side. In one of the studies, TEMPOL and viologen are bonded to water soluble polymers, which allows the use of a cheap Celgard separator^14^. However, at higher concentrations both systems lose capacity after only 100 cycles.

In addition to all-organic systems, RFBs that employ organics only on one side have been studied^11^,^12^. Here anthraquinone-2,7-disulfonic acid (AQDS(2,7)) and bromine in an acidic environment result in a cell voltage of 0.9 V, while 2,6-dihydroxy anthraquinone (AQDH(2,6)) and potassium ferricyanide in an alkaline environment results in a cell potential of 1.2 V^11^,^12^. A similar more recent advance is the use of an alloxazine-based (i.e. a vitamin B analogue) anolyte with ferrocyanide in an alkaline environment also producing a cell voltage of 1.2 V and high capacity retention^17^. Although discharge current densities in these RFBs are almost twice those observed for optimised vanadium RFBs, the maximum discharge power densities are about the same (0.4–0.6 W cm^−2^) because of the lower cell potentials^18^.

The possibility of tuning the solubility and redox potentials through substitutions of the side groups has recently been explored for quinones with density functional theory (DFT) calculations^19^,^20^. The results show that all-quinone aqueous RFBs with cell potential above 1.0 V is attainable by addition of electron withdrawing groups on hydroquinones and electron donating groups on anthraquinones.

However, so far there have been no systematic experimental studies of the actual change in redox potential and solubility with different functionalization. In the present study, we have experimentally determined redox potentials and aqueous solubility of 28 different benzo/hydroquinones (BQs), naphtoquinones (NQs) and anthraquinones (AQs) in acidic, neutral and alkaline solutions along with a few nitrogen based redox species. We discuss the limitations of their properties with respect to RFB performance and discuss some of the main challenges with respect to realising all-organic RFBs.

## Experimental Methods

**Figure 2:**
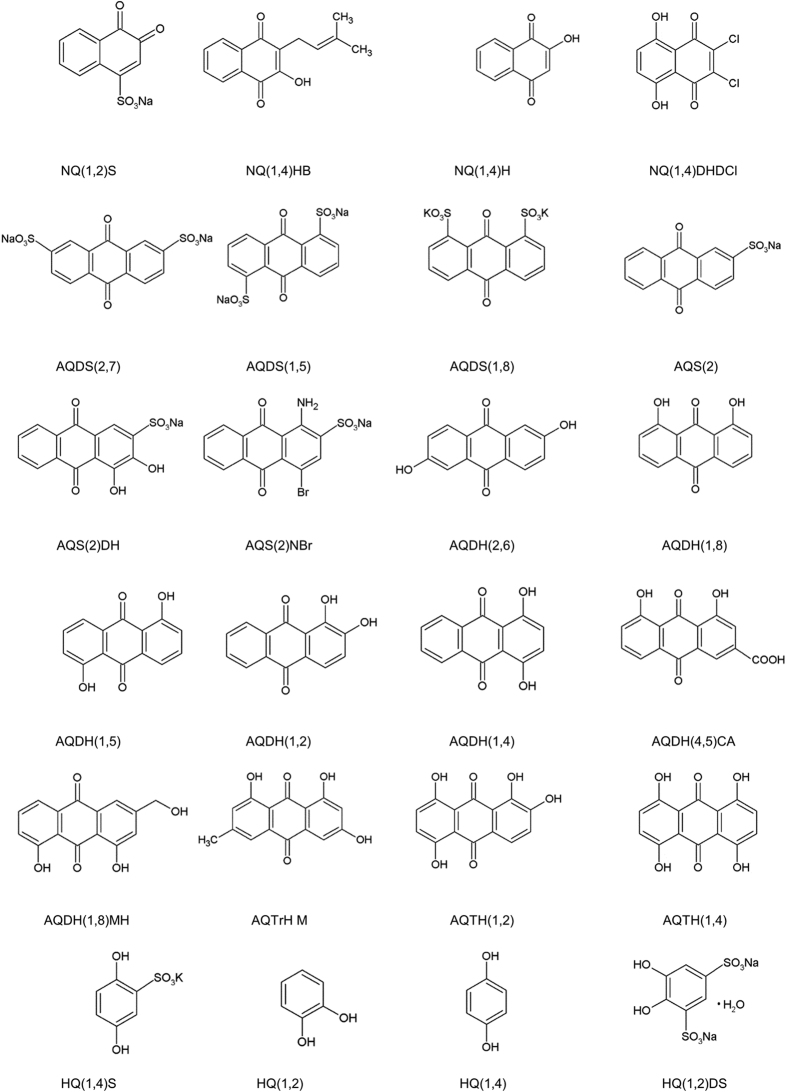
Structures and abbreviations of organic species. BQ = benzoquinone, HQ = hydroquinone, NQ = naphtoquinone and AQ = anthraquinone.

Figure 2 shows the structures of tested organic compounds as well as their abbreviations. In this work the denotation anthraquinone (AQ) is strictly the 9,10 isomer.

### Cyclic voltammetry studies

The investigated compounds were used as-purchased and without any purification. Supplier data and purity are found in Supplementary, S4. Cyclic voltammograms (CVs) were recorded with a Chemical Instruments potentiostat (CHI660E) using a standard three-electrode configuration consisting of a 3 M KCl Ag/AgCl reference electrode (+0.210 V_NHE_) (CHI111), a platinum wire counter electrode (CHI115) and a homemade circular 1 mm glassy carbon (GC) electrode prepared as reported elsewhere^21^. For each series of CVs, a freshly polished electrode was used. The CVs for soluble compounds in 1 mM concentration were recorded in a glass beaker with a customized lid in nitrogen purged solutions of three different pHs, namely pH 0 (1 M H_2_SO_4_), pH 7 (0.5 M phosphate buffer) and pH 13 (1 M NaOH). The pH of the NaOH solution is taken as 13 to account for carbonization of the solution with exposure to air. A background CV was recorded before each CV experiment to exclude background contaminations. The CVs of insoluble compounds in 1 M H_2_SO_4_, or 0.5 M phosphate buffer were recorded as follows: 6 mL of the supporting electrolyte was mixed with approximately 4 mL of acetone, and then a small amount of the quinone was added so that its concentration was around 1 mM. When the concentration of water in the mixture with organic solvent is greater than 7.2 M (here it was around 30 M) the separation between first and second reduction peak becomes 0 V^22^. This procedure gives a fair estimate of the redox potential of the quinone at pHs where it is insoluble in water. All CVs are found in Supplementary, S6.

### CV analysis

Each CV was analyzed in terms of peak potentials and peak currents. Peak currents were determined by subtracting a polynomial fit of the background from the recorded current as exemplified in Supplementary, S1. A plot of the absolute values of the peak currents to the square root of the scan rate was constructed and, when reasonable, a linear fit included to evaluate the electrochemical (Nernstian) reversibility of the electrochemical reaction. The peak current *I*_*p*_ for reversible reactions are given by equation (1) 23.


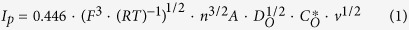


Where *F* is the Faraday constant, *R* the gas constant, *T* the temperature, *n* the number of electron transferred, *A* the area of the electrode, *D*_*O*_the diffusion coefficient and *C*_O_^*^the bulk concentration of the analyte and *v* the scan rate. The peak current ratio is expected to be close to one for reversible species. The formal redox potential (E^0^’), which is the adjusted form of the standard redox potential, where effects of the medium is taken into account, is reported here as the midpoint potential between the two peak potentials in the CV^24^. All half-cell potentials in this work refer to the normal hydrogen electrode (V_NHE_). All derived CV and solubility data are found in Supplementary, S4 and S5.

### RFB tests

A single 25 cm^2^ cell with carbon felt electrodes, graphite bipolar plates, copper current collectors and Nafion-117 ion exchange membrane was used. Prior to all battery tests the solutions were purged with nitrogen for 10 min and the tests were performed at room temperature. Carbon felt electrodes (4 mm thick, Sigracell) were pre-treated for one hour at 80 °C in H_2_SO_4_/HNO_3_ mixture (3:1 volumetric ratio) and then they were thoroughly washed with demineralized water. Nafion-117 was pre-treated in a following way: i) boiled for 30 min in 3% H_2_O_2_ aqueous solution, ii) washed with milliQ water and iii) left for one hour in 1 M H_2_SO_4_, phosphate buffer or KOH, depending on the pH value of the solution during RFB test. To assemble the cell, current collectors were screwed together by 6–11 bolts with bipolar plates, electrodes and Teflon + silicon gaskets in between. Two Grundfos DDA pumps circulated the electrolyte at a flow rate of 100 mL/min using Teflon tubing. The current was maintained constant at +/− 200 mA during tests using a Neware Battery Testing System CT-3008-5V3A-S1 (Shenzhen, China).

### Aqueous solubility estimation

Aqueous solubility (OECD test #105) was estimated in 1 M H_2_SO_4_, 1 M KCl and 1 M KOH prepared from MilliQ water^25^. Around 0.10 g of the pulverized compound was transferred to a glass tube after which increasing volumes of the supporting electrolyte solution was added in intervals of 10–100 μL depending on the expected solubility. After each addition the test tube was ultrasonicated and/or shaken vigorously using a Vortex Generator for at least 10 minutes at room temperature. Then the tube was inspected visually for undissolved sample and the addition of supporting electrolyte solution continued until the sample seemed just dissolved. The volumes just before and after full dissolution are used for calculating the solubility interval (Supplementary, S4 and S5) and the solubility is estimated as the average between the two. Addition of water was terminated when the solubility of the compound was less than 10 mM. Dissolved samples were left to stand for 12 hours and visually inspected for thixotropy using a vortex generator. For very insoluble compounds the amount of sample was decreased to 0.01 g and the dissolution time increased up to 24 h.

## Results and Discussion

### The electrochemical reaction of quinones in aqueous media

**Figure 3:**
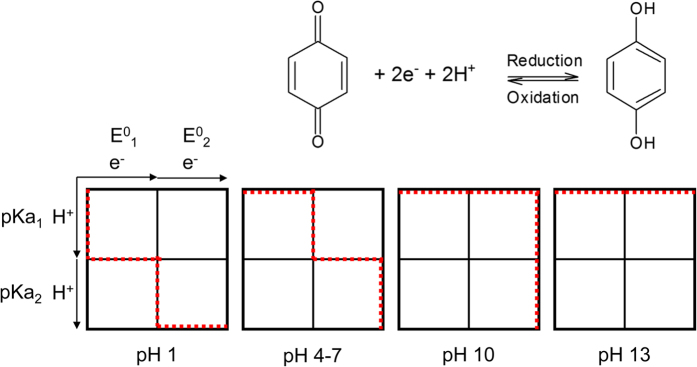
General quinone reduction at low pH (left) and scheme-of-squares for AQ-2,6-disulfonate and AQS(2) (right). Horizontal movement indicates electron transfer and vertical indicates proton transfer. They are guided by the redox potentials and pKa values of the quinone groups. Adapted from Batchelor *et al*.^26^.

Quinones generally undergo a reversible two-electron reduction/oxidation. The redox potential is strongly pH dependent, as the reaction may or may not involve two protons as illustrated in the scheme-of-squares mechanistic pathway in Fig. 3 26. At pH < pKa_1_, the reaction includes two protons and thus E^0^’ decreases with 59 mV per pH unit (at 25 °C)^24^. At pKa_1_ < pH < pKa_2_ and pKa_2_ < pH only one and no protons are involved, whereby E^0^’ decreases with 30 and 0 mV per pH unit, respectively. Generally, the reactions that include protons are slower and result in a lower electrochemical rate constant (*k*_0_) and may ultimately lead to higher charge-transfer resistance in the RFB. Thus, the choice of pH in the RFB environment strongly influences quinone electrochemical performance.

#### E^0^’ of anthraquinone derivatives

**Figure 4:**
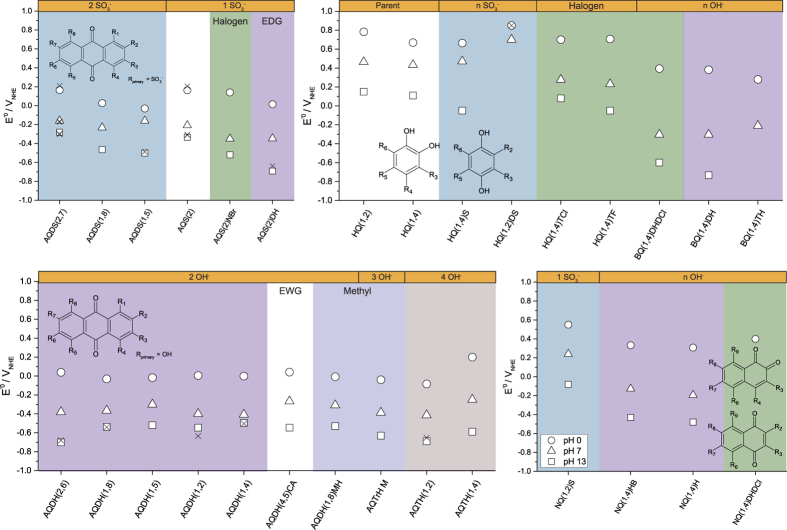
Formal standard potential determined from CV analysis at three different pH values for the tested quinones divided by group functionality. Crosses indicate literature values^11^,^12^,^13^,^26^,^27^. AQs are found to the left with the sulfonated on top and the hydroxylated in the bottom, while BQs and NQs are found to the right in the top and bottom, respectively. Numbering sequence of the substitution sites are indicated in the structures in the figure.

**Figure 5:**
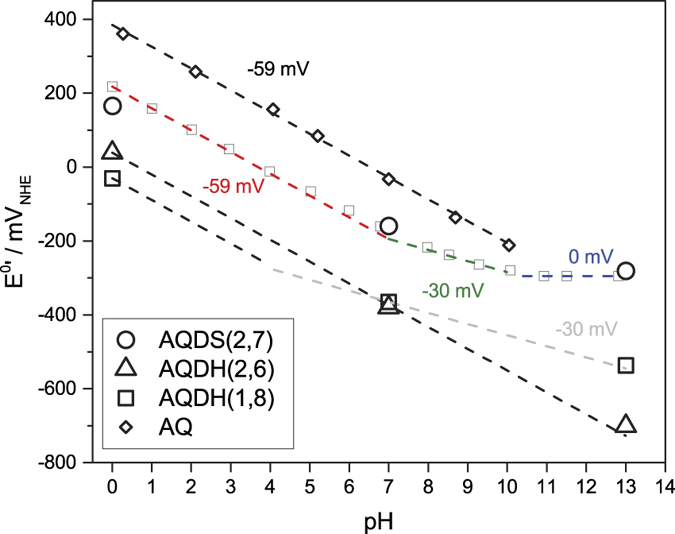
Pourbaix diagram for some AQ derivatives and pure AQ (Supplementary, S2) and input of lines with slopes −59 mV/pH unit for two-proton transfer and −30 mV/pH unit for one-proton transfer. All black marks refer to data from this report, while grey boxes refers to already published data^11^.

Figure 4 shows the experimental E^0^’ at three different pH values for all quinones in the study. Figure 5 shows a more detailed Pourbaix diagrams of selected AQ species, where it is seen that E^0^’ of unsubstituted AQ is about 300 mV lower than that of HQ(1,4) at pH 0. This illustrates that AQs are inherently electron rich species with redox potentials that are generally lower than those of the BQs, because unsubstituted AQ is in fact a BQ substituted with two benzene groups. In a recent DFT studies on a large ensample of quinones with various functional groups, it was found that it should be possible to change the standard redox potential from as high as 0.6 V_NHE_ to −1.5 V_NHE_^19^,^20^. Functional groups such as halogens, sulfonate or carboxyl groups were considered as electron withdrawing groups (EWGs) and hydroxyl and methyl groups as electron donating groups (EDGs). In this sense, higher redox potential would be expected for AQs with EWDs such as sulfonated AQs and lower for hydroxylated due to electron donation/withdrawal^19^. Nonetheless, the experimental data presented in Fig. 4 does not reproduce the full impact of this trend. It is seen that for sulfonated AQs, E^0^’ is only about 200 mV–300 mV higher than that of hydroxylated ones. Additionally, the position of sulfonate groups is very important, while the number seems to have very little effect. For instance, the two sulfonate groups in the edge positions in AQDS(2,7) decrease E^0^’ compared to the parent AQ from + 0.4 V_NHE,pH=0_ to + 0.165 V_NHE,pH=0_. Almost the same effect on E^0^’ is observed with only one sulfonate group in AQS(2), which has E^0^’ of + 0.163 V_NHE,pH=0_, indicating that sulfonate groups behave as EDGs by decreasing the potential, not as EWGs as expected. Changing only the positions of the sulfonate groups lowers the potential even further, by about 400 mV to + 0.03 V_NHE,pH=0_ for AQDS(1,8) and −0.03 V_NHE,pH=0_ for AQDS(1,5). This leads to the conclusion that when substituting with sulfonate groups, there is a higher electron donating effect in positions 1, 5 and 8 close to the quinone groups, while it is less pronounced in edge positions 2,7. This is also the case for edge position 6, as E^0^’ for AQDS(2,6) is around +0.166 V_NHE,pH=0_^26^.

Addition of two hydroxyl groups to the mono-sulfonated AQS(2) to give AQS(2)DH lowers E^0^’ to +0.015 V_NHE,pH=0_ indicating that the electron donating effect of hydroxyl groups is larger than that of one sulfonate group and similar to sulfonate group substitution closer to the quinone. Addition of one bromo and one amino group on the mono-sulfonated AQ to AQS(2)NBr lowers E^0^’ by only 20 mV at pH 0 indicating very small overall effect by these groups.

E^0^’ for the hydroxylated AQs is around 0 V_NHE,pH=0_, −0.4 V_NHE,pH=7_ and −0.6 V_NHE,pH=13_ and the variation is limited. Compared to E^0^’ of unsubstituted AQ of + 0.4 V_NHE,pH=0_, 0 V_NHE,pH=7_ and an estimated −0.4 V_NHE,pH=13_ (by extrapolation in Fig. 5), the electron donating effect of hydroxyl groups on AQ decreases E^0^’ by 200 mV–400 mV depending on pH. Again the experimental data here does not reproduce the large effect on E^0^’ expected from theoretical calculations. Addition of more hydroxyl groups does not lower the potential significantly, though the number of hydroxyl groups slightly affects E^0^’ in alkaline, which agrees well with DFT calculations^19^. For example, the tetrahydroxylated AQTH(1,4) has approximately 100 mV more negative E^0^’ compared to AQDH(1,4), but none have more negative E^0^’ than AQDH(2,6) at −0.70 V_NHE,pH=13_. AQDH(1,2) has an E^0^’ of −0.67 V_NHE,pH=13_, very similar to AQDH(2,6) indicating the importance of having a hydroxyl group in an edge position in order to reach the most negative potential. It is quite similar to AQTrH M which has three hydroxyl groups on the AQ (one edge group) and a methyl group on the edge giving E^0^’ of −0.69 V_NHE,pH=13_.

Similarly to sulfonated AQs, the positions of the hydroxyl groups affect E^0^’, but in acidic only to a small extent. Comparing dihydroxylated AQs, the two hydroxyl groups in AQDH(2,6) gives the most positive potential, of +0.04 V_NHE,pH=0_ while AQDH(1,5) and AQDH(1,8) is most negative (just below 0 V_NHE,pH=0_). In alkaline, E^0^’ of the hydroxylated AQs varies much more with position, by 200 mV from about −0.50 V_NHE,pH=13_ for AQDH(1,4), AQDH(1,8) and AQDH(1,5) to −0.70 V_NHE,pH=13_ for AQDH(2,6). The three former all have the hydroxyl groups on positions close to the quinone groups. The difference in E^0^’ for these hydroxylated AQs and others may be found in the pKa values of the central (hydro)quinone groups. Those are not trivial to measure given the insolubility at lower pH values for hydroxylated AQs, but they can be estimated from the Pourbaix diagram in Fig. 5 24,26. For instance, in the Pourbaix diagram of AQDS(2,7), E^0^’ follows the expected −59 mv/pH unit from pH 0 to pH 7 (pKa_1_). Then the first (hydro)quinone group deprotonates, and the slope changes to 30 mV/pH unit until pH 10 (pKa_2_) and E^0^’ becomes pH-independent. AQDH(1,8) shows a different dependence, where pKa_1_ is somewhere around pH 4 and pKa_2_ above pH 13. AQDH(2,6) shows an even more different behaviour as E^0^’ follows −59 mV/pH unit dependence straight to pH 13, which implies that both pKa values are higher than 13. The difference in protonation behaviour explains the observed more negative E^0^’ for AQDH(2,6) compared to AQDH(1,8) since it is only at higher pH than pKa_1_ of AQDH(1,8) around pH 4 that E^0^’ deviates between the compounds. Thus, if one can predict or measure reasonable values for these pKa values, it should be possible to predict which ones will have a more negative or positive E^0^’ at higher pH.

Taken together, it thus appears that the magnitude of the negative potentials that can be reached by hydroxyl group substitution is limited to about −0.7 V_NHE,pH=13_ and redox potentials below −1 V_NHE,pH=13_ appear unlikely. This is in good agreement with experimentally obtained E^0^’ of around −0.7 V_NHE,pH=13_ for hexahydroxy anthraquinone^27^. Irrespective of pH, EWGs placed on the edge of the dihydroxylated AQ do not alter E^0^’. This can be seen by comparing AQDH(4,5)CA with other AQDHs which have hydroxyl groups in similar positions. E^0^’ of AQDH(4,5)CA is around −0.53 V_NHE,pH=13_, which is similar to the other dihydroxylated AQs with hydroxyl groups in positions close to the quinone groups. Additionally, AQS(2)DH has both a hydroxyl and a sulfonate group in edge positions, and E^0^’ of +0.07 V_NHE,pH=0_ and −0.69 V_NHE,pH=13_, very close to that of AQDH(2,6). It virtually behaves as there is no EWG group.

#### E^0^’ of naphtoquinone and benzoquinone derivatives

Of the tested naphthoquinones, NQ(1,2)S show the highest E^0^’ of +0.6 V_NHE,pH=0_. The halogenated and hydroxylated NQ(1,4)DHDCl are electrochemically irreversible. Interestingly, the addition of one hydroxyl group in the case of NQ(1,4)H and NQ(1,4)HB shifts E^0^’ in alkaline by 0.8 V, to around −0.5 V_NHE,pH=13_, which gives a slope of 67 mV/pH unit. This is higher than observed for AQs, and lower than BQs, as will be described later. The potentials of NQs in alkaline are sufficiently negative for use as anolyte, provided sufficient solubility and stability can be reached.

It can be seen from Fig. 4 that ortho-BQs in general have an E^0^’ almost 100 mV higher compared to para-BQs. The most interesting results are the effect of substituting these two parent BQs with EWGs, and its implication on chemical stability. Although DFT calculations suggest that sulfonate group substitution on para-BQ should change E^0^’ by +100 mV, such an electron withdrawing effect was not observed experimentally as seen from HQ(1,4) and HQ(1,4)S where the opposite tendency is seen^19^. Similarly, the two sulfonate groups on ortho-BQ in HQ(1,2)DS increases E^0^’ by only +50 mV, which is less than predicted. The halogens, e.g. four fluoro or chloro groups (EWGs) on HQ(1,4)TC and HQ(1,4)TF show very small effect on the potential, which is in good agreement with the DFT calculations^19^. However, halogen substituents are prone to the nucleophilic attack of/in water and substitution reactions^28^. This can be verified by comparing the chemically irreversible CVs at pH 13 (Supplementary, S6, Figs 31 and 33). Chemical instability in water has also been reported for other benzoquinones, e.g. tetracyano benzoquinone^29^. E^0^’ was estimated to be +0.3 V higher than that of dicyano dichloro benzoquinone, which has E^0´^ of 0.9 V_NHE_, and also readily reacts with water^30^. Another example of a high-potential but very reactive benzoquinone is tetracarboxyl para benzoquinone which decomposes after synthesis and has a calculated standard potential of above +1 V_NHE_^19^,^31^. From the BQ CVs (Supplementary, Figures 25–33) they all generally appear (electro)chemically unstable in water.

Hydroxylation and methylation should affect E^0^’ towards more negative values^19^. Two hydroxyl groups on HQ(1,4)DH reduce E^0^’ by 300 mV at pH 0 compared to the parent BQ compound. Further reduction of E^0^ by substitution with more hydroxyl groups lowers the electrochemical reversibility. This is exemplified by BQ(1,4)TH which completely loses its electrochemical activity (Supplementary, S6, Figure 32). Other EDGs do not cause this instability, as BQ substituted with four methyl groups (duroquinone) has been reported to remain electrochemically reversible and chemically stable, with a potential of 0.5 V_NHE_ in aqueous (acidic and neutral) solutions^32^. Many methylated and alkylated BQs are natural compounds, i.e. Coenzyme Q10 (ubiquinone), duroquinone or plastoquinone. Generally, it appears that fully methyl and alkyl group substituted BQs are chemically stable in water, but this comes at the cost of a low E^0^’ and very low solubility.

Perhaps the most interesting effect from hydroxyl groups on BQs is their effect on the pH dependence of E^0^’. With just two hydroxyl groups in BQ(1,4)DH, E^0^’ changes from +0.38 V_NHE,pH=0_ to −0.73 V_NHE,pH=13_ corresponding to almost −90 mV/pH unit change. For para-BQ it changes from +0.70 V_NHE,pH=0_ to +0.11 V_NHE,pH=13_, and is only half (−45 mV/pH unit). This suggests that one of the two hydroxyl substituents also protonates/deprotonates in the electrochemical reaction adding around −30 mV/pH unit to the −59 mV/pH unit ending up at the total of −90 mV/pH unit. A similar effect is observed for BQ(1,4)DHDCl where the potential changes by 0.99 V for 13 pH units, which corresponds to −76 mV/pH unit. Both species have large negative E^0^’ in alkaline, −0.73 V_NHE,pH=13_ for the former and −0.60 V_NHE,pH=13_, for the latter. This suggests that E^0^’ is more affected by the pH of the supporting electrolyte than expected. To our knowledge this has not been described or explained in literature and it leads to the surprising conclusion that the E^0^’ of BQs are in the range, and in some cases even more negative than of those of AQs in alkaline. This could make them possible to use as anolytes in alkaline, provided it is possible to achieve fair aqueous solubility and in particular chemical stability.

### Chemical stability of organics in RFBs

The chemical stability of redox couples in RFBs is very important, since low life-time leads to impractical systems and increased *LCES*. By introducing organic molecules in high concentrations in oxidative/reductive environments, the possibility of (electro)chemical side reactions increases compared to state-of-the-art inorganic RFBs.

**Figure 6:**
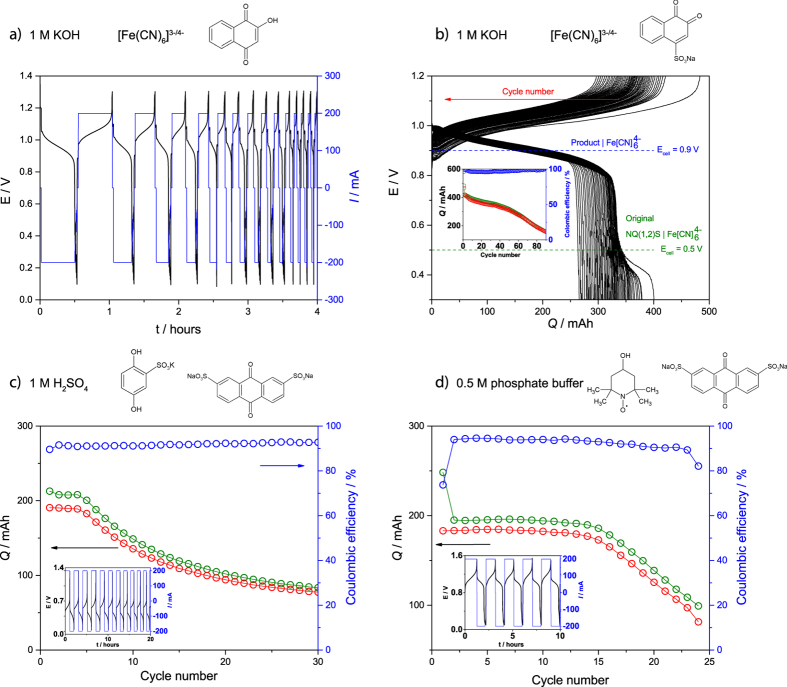
RFB tests. (**a**) Current and voltage characteristics versus time for a RFB with 200 mM solutions in 1 M KOH of NQ(1,4)H on the negative side and excess ferrocyanide on the positive side. There is significant capacity loss after only two cycles. (**b**) Capacity versus voltage curves for an RFB with 200 mM NQ(1,2)S and excess ferrocyanide in 1 M KOH. The inset shows the capacity (green for charging and red for discharging) and coulombic efficiency (blue) and significant capacity loss over 90 cycles. (**c**) Capacity and coulombic efficiency over time for an RFB comprised of 50 mM HQ(1,4)H and excess AQDS(2,7) in 1 M H_2_SO_4_. The inset shows current and voltage characteristics over time for the first eleven cycles. (**d**) Capacity and coulombic efficiency over time for an RFB comprised of 50 mM solutions of TEMPOL and AQDS(2,7) in 0.5 M phosphate buffer at pH 7 on the positive and negative side, respectively, for 24 cycles. The inset shows current and voltage characteristics over time for the first five cycles.

**Figure 7:**
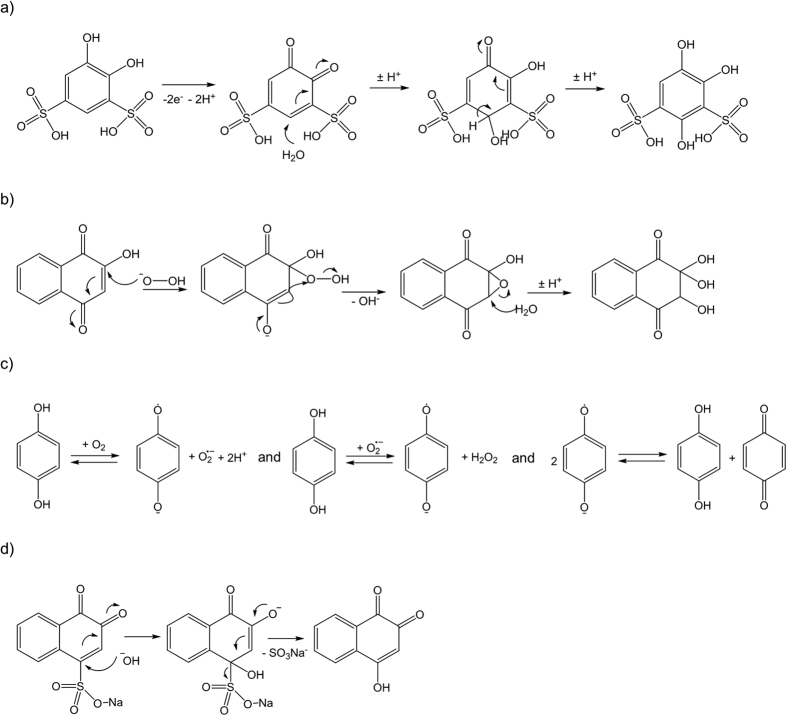
Possible degradation mechanisms of quinone species in Fig. 6 in the RFB environment. (**a**) Michael addition of water to the quinone form of HQ(1,2)DS that results from the electrochemical oxidation in the battery, (**b**) epoxidation and nucleophilic attack on NQ(1,4)H, c) superoxide and hydrogen peroxide formation with hydroquinones and (**d**) nucleophilic substitution reaction of NQ(1,2)S.

Figure 6 shows single cell flow battery tests of selected organic redox species mainly illustrating the challenges associated with stability of smaller, unsubstituted quinones. In Fig. 6a and b, a hydroxylated and sulfonated NQ are used on the negative side and fast, gradual capacity loss is seen. In Fig. 6c and d two possibilities for a small organic species for the positive side is tested and here capacity loss is observed after a small number of cycles. Generally, capacity loss in flow batteries can be attributed to redox species crossover through the membrane, oxidation from the outside environment and chemical degradation of redox species. Here major external oxidation is not likely since sealed and purged containers are used. Furthermore, a dense and highly selective Nafion membrane was used in all RFB tests displayed in Fig. 6 and crossover has been shown to be unlikely for the relatively large quinone molecules (HQ(1,2)DS, AQDS(2,7) and AQDH(2,6))^11^,^12^,^15^. For this reason we attribute the capacity loss to chemical degradation of the organic redox species. The interpretation is substantiated by considering the CVs of e.g. HQ(1,2)DS and HQ(1,4)S. At all pH values they show significantly low reduction/oxidation peak ratio, which indicate a fast side-reaction that follows the electrochemical oxidation (Supplementary, S6, Figure 25 and 28) and creates non-electroactive species. According to recent work, HQ(1,2)DS transforms to the tri- and tetrahydroxylated versions shortly after the oxidation to the quinone form, since water performs a Michael addition (1,4 to the α-β unsaturated carbonyl compound) as exemplified in Fig. 7a 15.

The multi-hydroxylated quinones are usually found to be electrochemically irreversible and/or insoluble. In the case of increasing the pH, a stronger nucleophile (OH^−^) is predominant and thus the reduction/oxidation peak ratio is even smaller than in acidic and neutral solution. This is confirmed further in an RFB battery tests in Fig. 6c, where an acidic all-organic RFB with HQ(1,4)S loses half of its capacity after only 30 cycles.

NQ(1,4)H has a E^0^’ of −0.5 V_NHE,pH=13_ and seemingly reversible CV (Supplementary, S6, Figure 13) in addition to being soluble in 1 M KOH making it is a fairly good anolyte candidate. However, RFB test shows significant capacity loss over time (Fig. 6a). Possible side-reactions includes a Michael-like conjugated addition of hydroxyl as in Fig. 7a for the sulfonated benzoquinone on the unsubstituted position (3), while the rest are protected by the benzene ring. In addition, an oxygen-sensitive possibility can be identified from the literature being the epoxidation with the hydroperoxide anion^33^. Epoxidation is usually followed by a nucleophilic attack here creating a non-aromatic and electrochemically irreversible hydroxylated species (Fig. 7b)^34^:

In practical RFB setups, there is always a risk of contamination with small amounts of oxygen which in the case of hydroquinones creates extremely reactive superoxides and usually hydrogen peroxide. Hydrogen peroxide, and the hydrogen peroxide anion created from it in alkaline solutions, are even better nucleophiles than the hydroxide ion itself. In addition, superoxides can oxidize a range of substituents and side groups. The formation is described in Fig. 7c.

Another example is an RFB test with NQ(1,2)S shown in Fig. 6b that also suffers from significant capacity loss. Here data indicates that the degradation process involves formation of a new redox active species giving a cell voltage of 0.9 V with ferrocyanide as seen from the main panel. This corresponds to the NQ compound having transformed to an electroactive hydroxylated form. Two possibilities can be readily identified, namely the addition of a hydroxyl group in position 3 in a Michael-like conjugated addition as earlier described in Fig. 7a or a nucleophilic substitution as that suggested in Fig. 7d 35. This reaction indicates how OH^−^ is a stronger nucleophile than water as its conjugate base pair and is able to substitute the usually bad leaving group sulfonate (see Fig. 7a for comparison in acidic media). The following capacity loss is then explained as in the case of NQ(1,4)H by further hydroxylation to electrochemically inactive species.

Obviously, there are stability issues with organic redox species and in particular with partially substituted quinones (NQs and BQs). The quinone contains a ketone functional group and a carbon-carbon double bond next to it (α,β unsaturated ketone). Because of the electron withdrawing property of the ketone, this double bond is prone to react with nucleophiles, while hydroquinone is a phenol derivative (aromatic) so because of the structural differences of these two compounds, they behave differently. Generally, small aromatic rings (e.g. the hydroquinone) are nucleophilic, so they are prone to react with electrophiles^34^. There is a difference in reactivity between the charged and discharged species: the phenolate form – as a conjugate base pair of the phenol – is a stronger nucleophile so in basic condition it reacts with quinones if it is sterically allowed. A range of nucleophilic reactions of quinones are available in the literature^36^. Nucleophiles such as H_2_O and hydroxyl ions are predominant in the RFB environment, especially in an alkaline solution so it is likely that the nucleophilic substitution of the quinone will be the greatest challenge in quinone stability^28^,^33^. In alkaline solution e.g. a sulfonate group can be substituted by a hydroxyl group as exemplified by the reaction in Fig. 7d while in acidic solution the same reaction can take place with water, but since the hydroxyl group is stronger nucleophile than water, the reaction is slower and the Michael-addition of Fig. 7a is more likely^28^.

Consequently, quinones with possible leaving groups and unsubstituted sites are likely to be unstable in aqueous solutions, however, the degradation reaction rate depends on the specific supporting electrolyte. A further complication is that as both oxidation states are present in the battery during operation and hydroquinones can act as nucleophiles and react with the benzoquinones, there can be unwanted polymerizations at both low and high pH^37^,^38^. Thus, when considering stability the use of only partially substituted BQs and NQs and those substituted with possible leaving groups in aqueous RFBs is very limited.

As an alternative to quinones, the nitroxide radical TEMPOL, was tested. It has reversible CVs at pH 7, and no degradation even after 30 days in aqueous buffer in the uncharged state (Supplementary, S7). However, in a great number of RFB battery tests of which an example seen in Fig. 6d, it performed well for only a limited number of cycles, after which significant capacity loss was observed, even at low concentrations. This behaviour is similar to previous studies^16^,^14^. It has been shown that the similar TEMPO radical disproportionates to the N-oxo ammonium salt in warm acidic water and further on to the hydroxylamine with the possibility of further dimerization^39^: While the temperatures in the RFB environment are generally lower than those studied, it is plausible that the TEMPOL radical reacts in a similar manner after a couple of cycles, which would explain the capacity loss observed in Fig. 6d. A few other nitrogen-containing redox active species (Fig. 2 and Supplementary, S7) were included. The general conclusion from this data is that the pyrazine-derived heteroaromatics are only seemingly stable only at pH values between 0 and 3 and have quite negative potentials.

### Aqueous solubility of quinones in different supporting electrolytes

Potentially, an all-quinone RFB can reach the same energy density (30 WhL^−1^) as the vanadium RFB given a cell potential of 1 V and a solubility of 1 M^40^. Apart from some of the sulfonated anthraquinones/benzoquinones, the properties of quinones in aqueous solution is that of weak acids or salts of weak acids and strong bases. In that case their solubility can be described by the Henderson–Hasselbalch equation^41^:


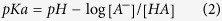


**Figure 8:**
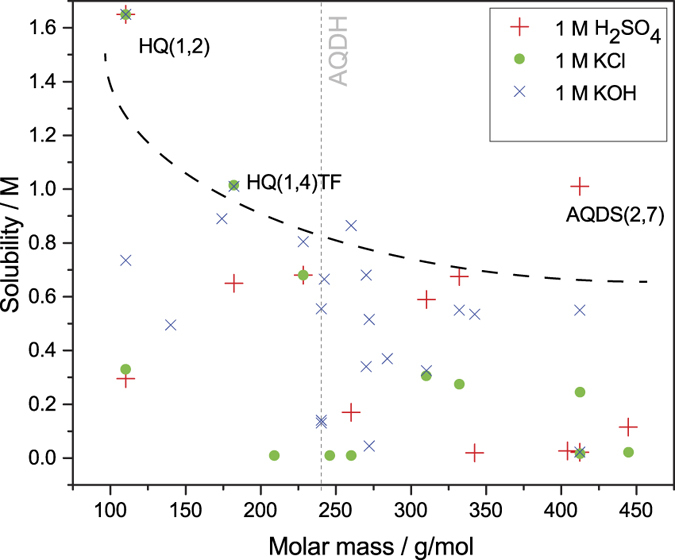
Overview of the experimentally estimated solubility range of the quinones in three different supporting electrolytes of 1 M concentration as a function of their molar mass. The dotted line is a guide for the eye indicating maximum solubility. Solubility data are found in Fig. 7 and solubilities <10 mM are not included in the figure.

The total concentration of species dissolved in water (*S*_*tot*_) is the sum of the concentration of dissociated species ([*A*^*−*^]) and the non-dissociated species ([*HA*]). If [*HA*] is replaced with the intrinsic solubility of the species (*S*_*0*_) from above, and [*A*^*−*^] = *S*_*tot*_ *− S*_*0*_, the total solubility can be presented as^41^:


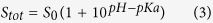


by rearranging equation 2. From equation 3, one can conclude that solubility/miscibility of the quinones in/with water can be further enhanced if the substituent groups dissociate in water, i.e. their pKa values are low and preferably the pH is high. In this report, the aqueous solubility of the chemicals was measured as-received. In the case of the BQs many of them are in hydroquinone form, which gives higher solubility compared to the quinone form. For instance it is expected that HQ(1,4)TF or HQ(1,2) have very low solubility in water in their benzoquinone form. Figure 8 show that quinones tested in this work in general do not show aqueous solubility greater than 1.6 M (HQ(1,2)), in any supporting electrolyte. One would expect a decreasing maximum solubility with the molar mass, since the dissolved mass and volume of the molecules becomes comparable to that of water. However, such behaviour cannot be clearly observed from Fig. 8 especially given the high solubility of AQDS(2,7).

**Figure 9:**
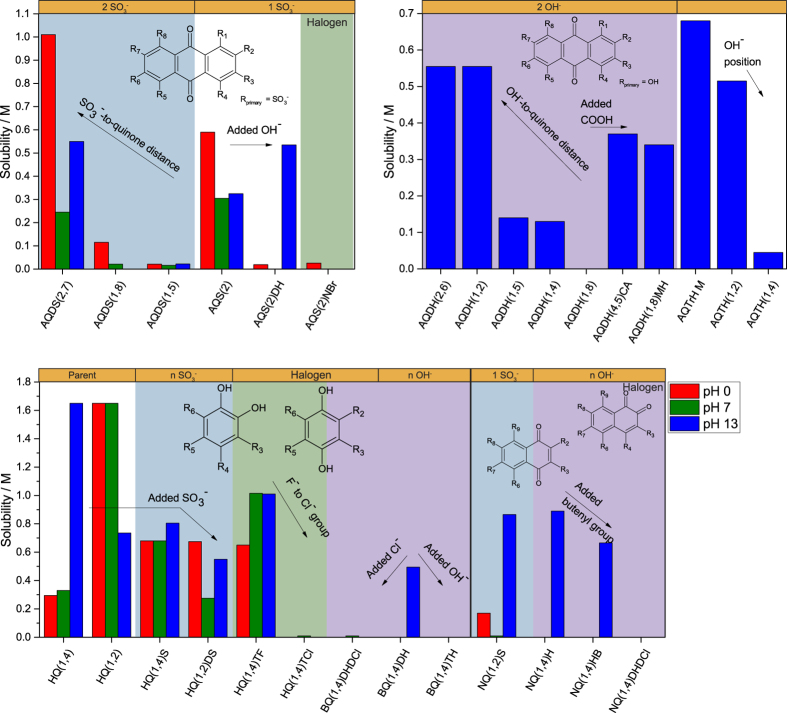
Experimentally estimated solubility of the quinone species in 1 M H_2_SO_4_ (red), 1 M KCl (green) and 1 M KOH (blue). Sulfonated AQs (top left), hydroxylated AQs (top right) and BQs and NQs (bottom). Solubilities <10 mM are denoted as having zero solubility. Numbering sequence of the substitution sites are indicated in the structures in the figure.

The solubilites of all quinones are shown in Fig. 9 sorted by quinone type and functional groups. The general trend is that sulfonated quinones have high solubility in all supporting electrolytes, while the hydroxylated are soluble only in alkaline. This is reasonable, as hydroxyl groups in general have much higher pKa values, so higher pH is needed to deprotonate them to salts. Most halogen substituted quinones, exemplified by AQS(2)NBr, HQ(1,4)TC, BQ(1,4)DCDH and NQ(1,4)DHDC, are insoluble in water and limits their practical use in aqueous flow batteries.

The solubility of all of the sulfonated AQs is lower in 1 M KCl and KOH than in 1 M H_2_SO_4_. Considering the sulfonate group position, AQDS(2,7) is the most soluble followed by AQS(2) while AQDS(1,8) and AQDS(1,5) shows more than ten times lower solubility. For these compounds, solubility and redox potential follow the same trend where solubility increases with E^0^’. With addition of hydroxyl or halogen groups to sulfonated AQs, i.e. AQS(2)DH and AQS(2)NBr, the compounds become barely soluble in 1 M H_2_SO_4_ and almost insoluble in 1 M KCl. The two hydroxyl groups on AQS(2)DH does however considerably increase solubility in 1 M KOH.

Hydroxylated AQs are almost insoluble in 1 M H_2_SO_4_ and KCl which limits their practical use to only alkaline RFBs. This also indicates that *S*_*0*_ for these compounds is low, and that the solubility in alkaline is almost entirely related to the pKa of the substituent groups. Changing the hydroxyl group positions to 1,4,5 and 8 in AQDHs dramatically reduces the solubility, this is the same trend as observed for sulfonated AQs. In general, whenever the edge substituent is a hydroxyl group, the solubility is between 0.5 and 0.7 M.

The highest solubility observed in alkaline is for the trihydroxylated and methylated AQTrHM. When substituting the otherwise insoluble AQDH(1,8) with carboxylate or hydroxymethyl on the edge groups, the solubility increases to above 0.3 M. A higher number of hydroxyl groups can reduce the solubility in alkaline, as exemplified by AQTH(1,4), with four hydroxyl groups, which has only half the solubility compared to AQDH(1,4). However, this effect is not observed for AQTH(1,2), which again underlines the importance of edge group substitution compared to positions closer to the ring.

NQs are more soluble in alkaline than AQs. NQ(1,2)S has high solubility of 0.9 M, while NQ(1,4)H has solubility of 0.82 M, Interestingly, addition of the large butenyl group on NQ(1,4)H in NQ(1,4)HB only slightly reduces the solubility to 0.70 M in alkaline.

#### Thixotropy of AQs

When performing solubility tests, thixotropy was observed for a number of compounds^42^. In practical RFBs thixotropy is not wanted, as the redox solutions can be kept still for long times. Samples were observed to dissolve well in the supporting electrolyte under agitation, but when left for hours it turned into a thick paste that could be brought back to the liquid state by repeated agitation. This was most notably observed for AQDS(2,7) in all supporting electrolytes (tested in the disodium form as received) and for a number of hydroxylated AQs in 1 M KOH solution (namely AQS(2)DH, AQDH(1,4), AQDH(1,2), AQDH(1,8)MH, AQDH(4,5)CA). Thixotropy of AQ dyes in aqueous dispersions has been described earlier, as well as some methods to overcome the problem such as addition of polyvinylpyrrolidone to the solution^43^.

## Conclusions & future perspectives

By combining the quinones with the highest and lowest E^0^’ (HQ(1,2)DS and AQTH) a hypothetical cell potential for an all-quinone RFB can be as high as 1.1 V at pH 7, while at pH 0 and 13 it could be around 0.9 V. A cell potential in between 0.9 V and 1.1 V provide fair energy density of an RFB, provided the compounds on both sides have sufficient water solubility (>1 M) and chemical stability.

Currently, only semi-organic RFBs appear feasible, since many AQ derivatives have sufficiently low E^0^’ for the negative side together with high solubility and seemingly high chemical stability, while the positive side remains a challenge. Both sulfonated and hydroxylated AQs, which are low cost compounds used in large quantities in the dye industry, are examples of promising anolytes, however future studies are needed to determine their long term stability.

Most AQ derivatives have a negative E^0^’ with a rather narrow variation between different functionalized derivatives. This behaviour is not fully predicted by DFT calculations and it is doubtful whether redox potential improvements to the already reported AQs, AQDS(2,7) and AQDH(2,6) for use as anolytes in aqueous RFBs can be realised. The solubility of AQDH(2,6) might be improved by further functionalization in the edge positions.

The experimental data presented here, together with existing work in literature, shows that the main challenge is to develop highly soluble and chemically stable organic redox species for the positive side with a sufficiently high E^0^’ that would enable a cell potential >1 V. Benzoquinones generally have the most positive E^0^’ from around +0.4 to +1.2 V_NHE,pH=0_. However, currently known BQs with high E^0^’ are not stable in aqueous solutions which is exemplified by most studied BQs in this work.

While it is clear that functional groups are needed to enhance the solubility and chemical stability of benzoquinone compounds and obtain the desired redox potential for practical applications, the experimental data here shows that this functionalization is not simple, and could possibly add significantly to the BQs costs. A way of addressing the stability issue could be synthesis of fully substituted BQs with more stable C-R or C-C-R bonds, where R is a functional substituent i.e. hydroxyl, sulfonate or other groups (e.g. phosphates) that increases solubility and does not compromise the redox potential. An alternative approach is water soluble polymers with immobilized and fully substituted benzoquinone species as has been attempted with the TEMPOL radical.

## Additional Information

**How to cite this article**: Wedege, K. *et al*. Organic Redox Species in Aqueous Flow Batteries: Redox Potentials, Chemical Stability and Solubility. *Sci. Rep.* **6**, 39101; doi: 10.1038/srep39101 (2016).

**Publisher's note:** Springer Nature remains neutral with regard to jurisdictional claims in published maps and institutional affiliations.

## Supplementary Material

Supplementary Information

## Figures and Tables

**Figure 1:**
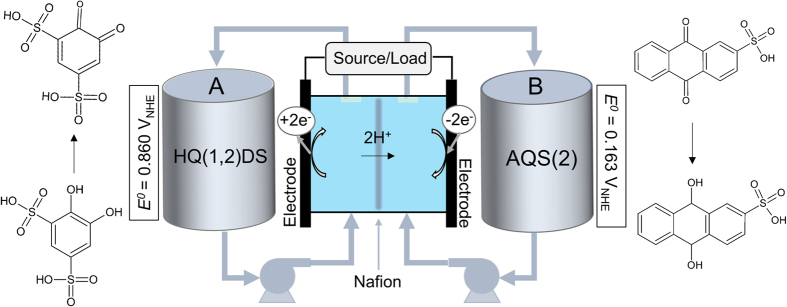
Schematic of an all-quinone RFB during charging^13^. Quinone species are dissolved in 1 M H_2_SO_4_, and the cell potential is 0.7 V. During charging each quinone stores 2 electrons while at the same time 2 H^+^ are exchanged through Nafion.
