# Supplementary material for: Organic Redox Species in Aqueous Flow Batteries: Redox Potentials, Chemical Stability and Solubility
Source: Sci Rep. 2016 Dec 14;6:39101. doi: 10.1038/srep39101 (PMC5155426; doi:10.1038/srep39101)
Supplement: Supplementary Information [file srep39101-s1.pdf]

Supplementary information

Organic Redox Species in Aqueous Flow Batteries: Redox Potentials, Chemical Stability and Solubility

Kristina Wedege,[a] Emil Drazevic*[a], Denes Konya[b], and Anders Bentien*[a]

a: Department of Engineering - Aarhus University, Hangøvej 2 8200 Aarhus N, Denmark.

Email: edrazevic@eng.au.dk, bentien@eng.au.dk

b: Research Centre for Natural Sciences, Magyar Tudósok körútja 2, 1117 Budapest, Hungary.

Supporting information content

S0: Symbols, abbreviations and standard chemicals
S1: CV background subtraction examples
S2: Pure Anthraquinone Pourbaix diagram
S3: RFB cell
S4: Collected data for quinones
S5: Collected data on nitrogen containing compounds
S6: CV data of organic species
S7: TEMPOL stability
S8: Examples of side reactions
S9: Cost estimation of organic redox species

S0: Symbols, abbreviations and standard chemicals

Symbol/Abbreviation	Definition
CV	Cyclic Voltammetry / voltammogram
Epa	Anodic peak pontential
Epc	Cathodic peak potential
ipa	Anodic peak current
ipc	Cathodic peak current
V_{NHE}	Potential in volts reference to the Normal Hydrogen Electrode
v	Scan rate [V/s]
GC	Glassy carbon
$E^{'0}$	Formal standard potential

Compound	Purity	Supplier
H_2SO_4	95-98%	Sigma-Aldrich
KOH	90 %	Sigma-Aldrich
KCl	99 %	Sigma-Aldrich
KOH	90% (flakes)	Sigma-Aldrich
NaOH	32% solution analytical grade	AppliChem

S1: CV background subtraction examples

Each CV was analyzed in terms of peak potentials and peak currents. Peak currents were determined by manually subtracting a polynomial fit (3rd-5th order depending on the visual fit) of the background from the recorded current as exemplified in Figure 1.

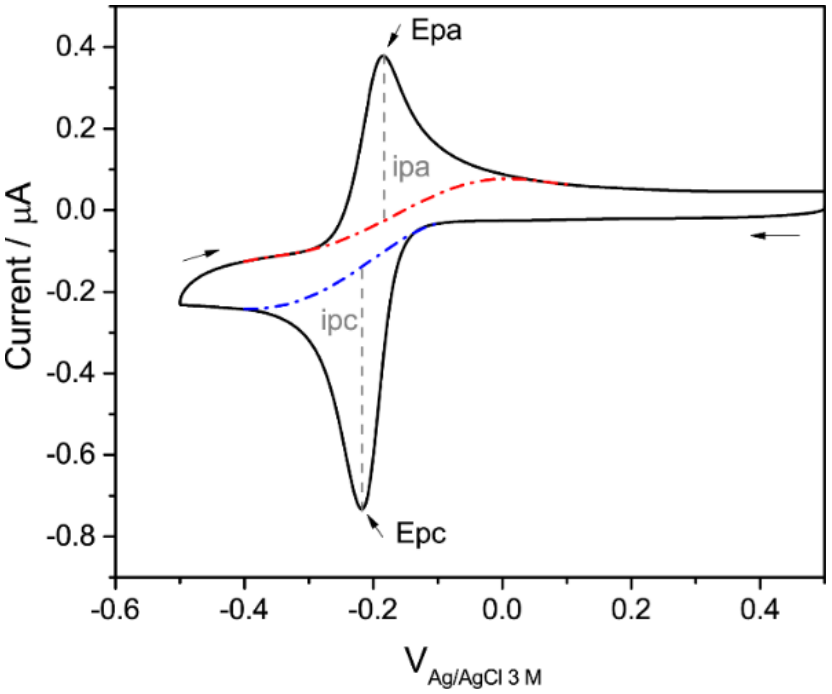

Figure 1: CV with polynomial fitted background currents subtracted from the measured currents to get background corrected values of ipa and ipc.

S2: Pure Anthraquinone Pourbaix diagram

The redox potential of pure 9,10-anthraquinone (Sigma-Aldrich, 97%) was estimated in aqueous solutions at different pH values by measuring the CV response of a GC electrode that had been freshly grinded with anthraquinone mixed with acetone to yield a surface covered/attached species. The CVs are shown in Figure 2, and it is noticed that as the pH increases, there is a significant side-reaction, and it was only possible to obtain reliable data up to pH 10. Thus, the redox potential at pH 13 is only extrapolated. The solutions were freshly prepared and were: 1 M H_2SO_4 for pH 0, 0.2 M HCl/KCl buffer for pH 2, Reagecon Technical buffers for pH 4 and 7, 0.2 M acetate buffer for pH 5 and Radiometer Analytical buffer (carbonate) for pH 10.

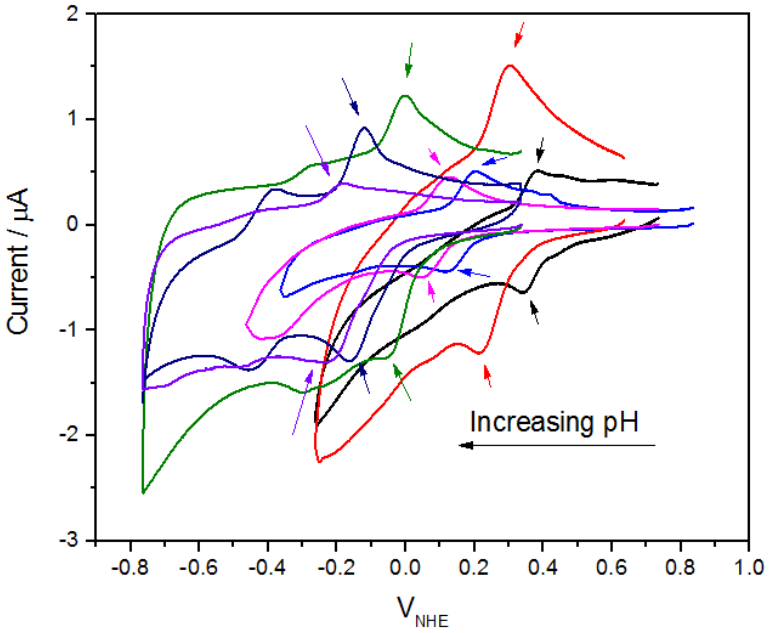

Figure 2 CVs for the anthraquinone-grinded GC electrode at different pH values

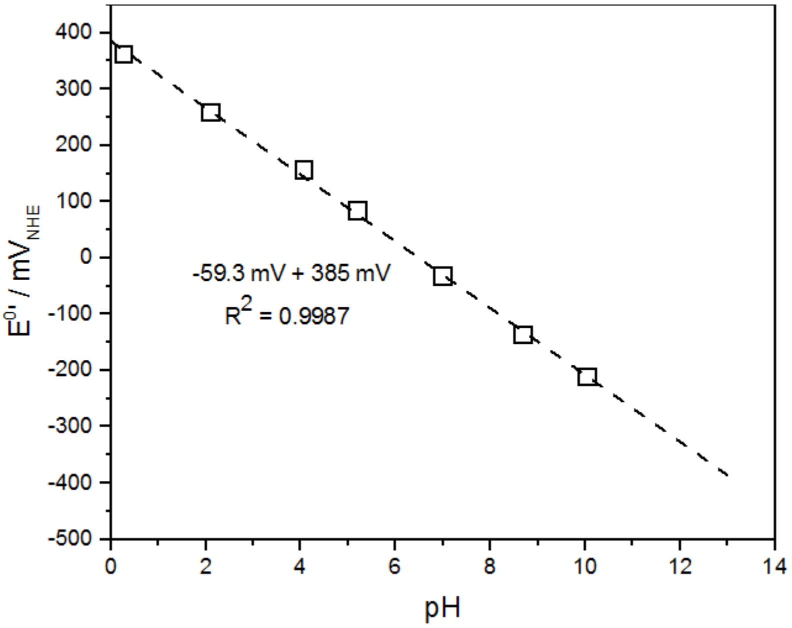

Figure 3: The observed standard potential as a function of pH (right). The line represents linear regression data. Colored arrows indicates the peaks interpreted as the pure AQ peaks used for the Pourbaix diagram.

S3: RFB cell

In total four RFB tests were performed in this work in a 25 cm^2 cell depicted in Fig. 3.

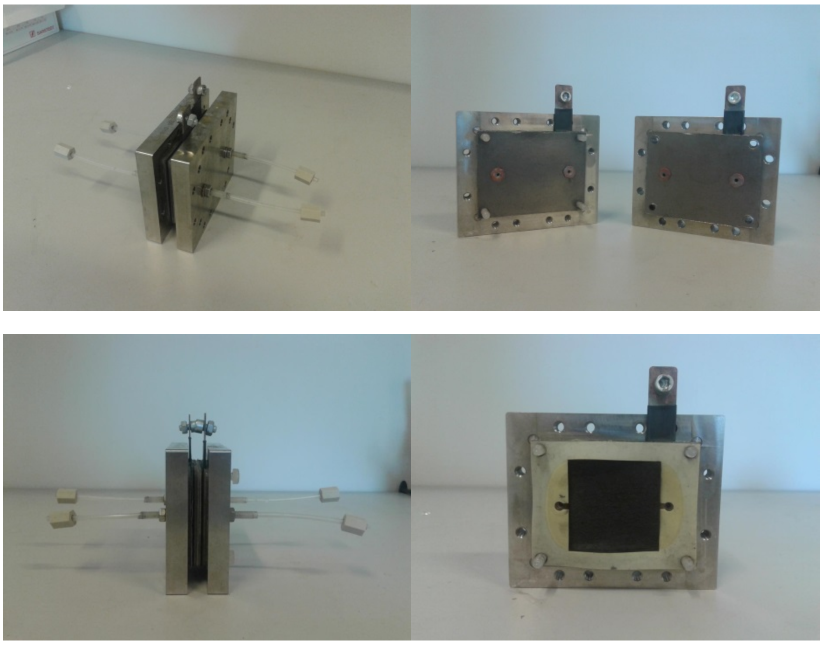

Figure 4: RFB cell used in this work

S4: Collected data for quinones

The following tables 1-4 collects the names, abbreviations, supplier, purity data, determined formal redox potential and solubility at three different pHs as well as comments on stability and solubility. CV data are found in S5.

Table 1a: Solubility and electrochemistry data collected on sulfonated anthraquinones (1-3 of 6).

**Structure**		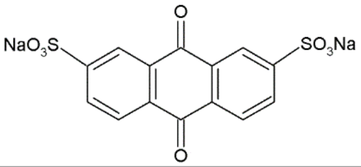	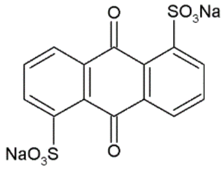	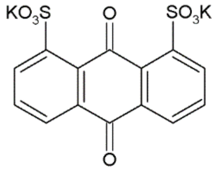
**Name**		9,10-anthraquinone-2,7-disulfonate disodium	9,10-anthraquinone-1,5-disulfonate disodium	9,10-anthraquinone-1,8-disulfonate dipotassium
**Abbreviation**		AQDS(2,7)	AQDS(1,5)	AQDS(1,8)
**Supplyer**		TCI	TCI	TCI
**Purity**		95%	98%	98%
**Molarity / M**		412.29	412.29	444.51
**E'^0 / V_{NHE}**	**pH 0**	0.165(5)	- 0.03(4)	0.027(4)
	**pH 7**	- 0.16(1)	- 0.16(1)	- 0.232(7)
	**pH 13**	- 0.281(2)	- 0.501(2)	-0.464(4)
**Peak current ratio**	**pH 0**	0.7(1)	1.7(1)	1.04(7)
	**pH 7**	0.56(3)	0.49(3)	0.4(1)
	**pH 13**	0.96(7)	1.17(4)	1.0(3)
**Solubility / M**	**1 M H_2SO_4**	1.21-0.81	0.12-0.11	0.022-0.02
	**1 M KCl**	0.27-0.22	0.024-0.018	0.017-0.015
	**1 M KOH**	0.61-0.49	< 0.01	0.029-0.015
**Electrochemical reversibility**		Quasireversible at pH 0 and 7<br>Reversible at pH 13	Quasireversible at pH 0 and 7<br>Reversible at pH 13	Quasireversible at pH 0 and 7<br>Reversible at pH 13
**Comment**		Thixotropic behaviour	Phase separation upon standing	Phase separation upon standing

Table 1b: Solubility and electrochemistry data collected on sulfonated anthraquinones (3-6 of 6).

**Structure**		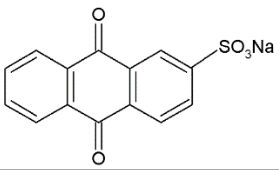	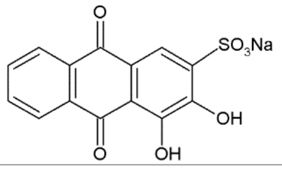	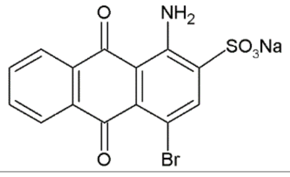
**Name**		9,10-anthraquinone-2-sulfonate sodium	Alizarin red s 3,4-dihydroxy-9,10-anthraquinone-2-sulfonate sodium	1-amino-4-bromo-9,10-anthraquinone-2-sulfonate sodium
**Abbreviation**		AQS(2)	AQS(2)DH	AQS(2)NBr
**Supplyer**		Sigma-Aldrich	Sigma-Aldrich	AK Scientific
**Purity**		98%	99%	98%
**Molarity / M**		310.26	342.26	404.171
**E'^0 / V_{NHE}**	**pH 0**	0.163(3)	0.015(4)	0.14(4)
	**pH 7**	- 0.208(1)	- 0.347(1)	≈ - 0.35*
	**pH 13**	-0.334(3)*	- 0.6918(2)	≈ - 0.52*
**Peak current ratio**	**pH 0**	1.0(1)	0.9(1)	0.4(1)
	**pH 7**	0.88(1)	0.85(6)	
	**pH 13**	1.2(2)	1.1(1)	-
**Solubility / M**	**1 M H_2SO_4**	0.64-0.54	0.021-0.017	0.027-0.025
	**1 M KCl**	0.32-0.29	< 0.01	
	**1 M KOH**	0.36-0.29	0.58-0.49*	< 0.01
**Electrochemical reversibility**		Reversible	Quasieversible	Quasireversible
**Comment**		Phase separation upon standing * Double peak at high scan rates largest peak reported	* Thixotropic behavior in KOH	*Average of double peak

Table 2: Solubility and electrochemistry data collected on naphotquinones.

**Structure**		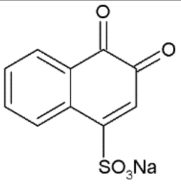	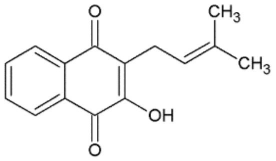	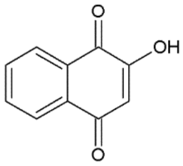	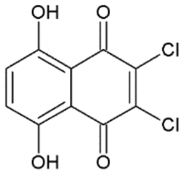
**Name**		1,2-naphtoquinone-4-sulfonate sodium	*Lapachol* 2-hydroxy-3-(3-methyl-2-butenyl)-1,4-naphthoquinone	2-hydroxy-1,4-naphtoquinone	2,3-dichloro-5,8-dihydroxy-1,4-naphthoquinone
**Abbreviation**		NQ(1,2)S	NQ(1,4)HB	NQ(1,4)H	NQ(1,4)DHDCl
**Supplyer**		TCI	Santa Cruz Biotechnology	TCI	TCI
**Purity**		98%	ND	98%	97%
**Molarity / M**		260.19	242.27	174.16	259.04
**E'^0 / V_{NHE}**	**pH 0**	0.55(1)	0.333(3)*	0.308(4)	-
	**pH 7**	0.24(1)	- 0.128(7)	- 0.194(3)	
	**pH 13**	- 0.081(4)*	-0.43(3)	- 0.48(1)	≈ 0.4*
**Peak current ratio**	**pH 0**	0.8(1)	-	0.81(8)	-
	**pH 7**	0.52(3)	1.08(5)	1.2(3)	
	**pH 13**	0.9(1)	0.38(4)	0.73(6)	
**Solubility / M**	**1 M H_2SO_4**	0.19-0.15	< 0.01	< 0.01	< 0.01
	**1 M KCl**	≈ 0.01			
	**1 M KOH**	0.96-0.77	0.70-0.63	* 0.96-0.82	
**Electrochemical reversibility**		Quasireversible	Reversible at pH 7 Quasireversible at pH 0 and 13	Quasireversible	Irreversible
**Comment**		* From 3 first voltammograms	*Average of 4 observed peaks	* Slight phase separation upon standing in KOH	*2 irreversible oxidation peaks at pH 13 at ≈ -0.3 V_{NHE} and ≈ 0.4 V_{NHE}

Table 3a: Solubility and electrochemistry data collected on hydroxylated anthraquinones (1-4 of 10).

**Structure**		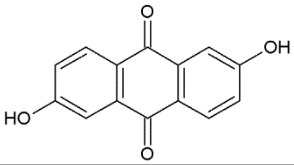	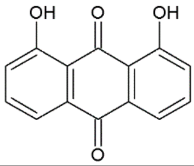	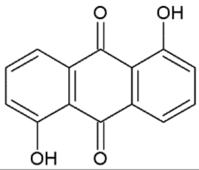	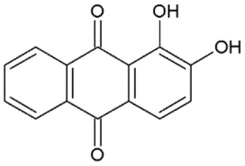
**Name**		*Anthraflavic acid* 2,6-dihydroxy-9,10-anthraquinone	*Chrysazin* 1,8-dihydroxy-9,10-anthraquinone	*Anthrarufin* 1,5-dihydroxy-9,10-anthraquinone	*Alizarin* 1,2-dihydroxy-9,10-anthraquinone
**Abbreviation**		AQDH(2,6)	AQDG(1,8)	AQDH(1,5)	AQDH(1,2)
**Supplyer**		AK Scientific	TCI	TCI	TCI
**Purity**		90%	98%	85%	90%
**Molarity / M**		240.21	240.21	240.21	240.2
**E'^0 / V_{NHE}**	**pH 0**	0.039(4)	- 0.03*	-0.017(1)*	0.005(4)
	**pH 7**	- 0.38(1)	- 0.365(3)*	- 0.3**	- 0.400(6)
	**pH 13**	-0.701(6)	- 0.537(4)	- 0.520(2)	- 0.546(2)*
**Peak current ratio**	**pH 0**	0.83(4)	-	-	0.73(7)
	**pH 7**	0.9(1)			0.5(1)
	**pH 13**	1.1(1)	1.0(1)	0.79(9)	1.2(1)
**Solubility / M**	**1 M H_2SO_4**	< 0.01	< 0.01	< 0.01	< 0.01
	**1 M KCl**				
	**1 M KOH**	0.59-0.52		0.15-0.13	0.59-0.52**
**Electrochemical reversibility**		Reversible at pH 13	Reversible at pH 13	Quasireversible at pH 13	Quasireversible at pH 13
**Comment**			Concentration for electrochemical test: 0.25 mM *electrode reaction	* Electrode reaction, two peaks, largest reported **estimated mid-point potential of 2 broad peaks	*Double peak at high scanrates pH 0 and7 show electrode reaction ** Thixotropic behavior in KOH

Table 3b: Solubility and electrochemistry data collected on hydroxylated anthraquinones (5-7 of 10).

**Structure**		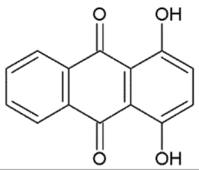	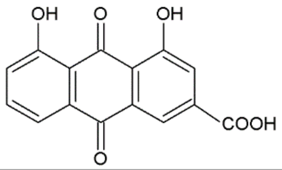	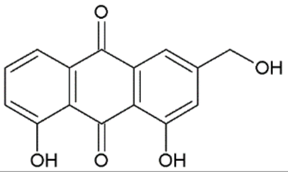
**Name**		*Quinizarin* 1,4-dihydroxy-9,10-anthraquinone	*Rhein* 4,5-dihydroxy-9,10-anthraquinone-2-carboxylic acid	*Aloemodin* 1,8-dihydroxy-3-(hydroxymethyl)-9,10-anthraquinone
**Abbreviation**		AQDH(1,4)	AQDH(4,5)CA	AQDG(1,8)MH
**Supplyer**		TCI	Shanghai Tianfu Chemical Limited	Spring & Autumn
**Purity**		98%	Not disclosed	98%
**Molarity / M**		242.23	284.22	270.24
**E'^0 / V_{NHE}**	**pH 0**	- 0.001(5)	0.041(9)*	- 0.008(9)
	**pH 7**	- 0.407(1)*	-0.267(1)	- 0.309(7)
	**pH 13**	- 0.496(3)	-0.546(2)**	- 0.529(4)
**Peak current ratio**	**pH 0**	0.6(1)	0.39(6)	0.58(4)
	**pH 7**	0.4(2)	0.69(8)	0.51(4)
	**pH 13**	1.05(3)	1.23(7)	1.03(1)
**Solubility / M**	**1 M H_2SO_4**	< 0.01	< 0.01	< 0.01
	**1 M KCl**			
	**1 M KOH**	0.15-0.11**	0.39-0.35***	0.37-0.31*
**Electrochemical reversibility**		Reversible at pH 13	Quasireversible at pH 7<br>Reversible at pH 13	Reversible at pH 13
**Comment**		*Multiple peaks at pH 7 & shows electrode reaction<br>** Thixotropic behavior in KOH	*Multiple peaks (quinone peak reported) and electrode reaction<br>**double peak at high scanrates<br>*** Thixotropic behavior in KOH	*Thixotropic behavior in KOH

Table 3c: Solubility and electrochemistry data collected on hydroxylated anthraquinones (7-10 of 10).

**Structure**		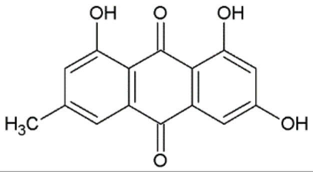	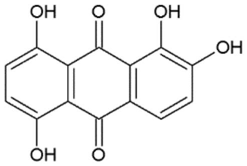	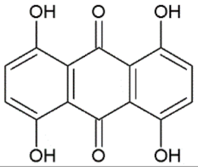
**Name**		Emodin<br>1,3,8-trihydroxy-6-methylanthracene-9,10-dione	*Quinalizarin*<br>1,2,5,8-tetrahydroxy-9,10-anthraquinone	1,4,5,8-tetrahydroxy-9,10-anthraquinone
**Abbreviation**		AQTrHM	AQTH(1,2)	AQTH(1,4)
**Supplyer**		Spring & Autumn	Merck	ALFA
**Purity**		98%	Analytical grade	96%
**Molarity / M**		270.23	272.21	272.2
**E'^0 / V_{NHE}**	**pH 0**	-0.04(1)	-0.084(5)	0.200(4)*
	**pH 7**	-0.386(2)	-0.414(3)*	- 0.249(4)*
	**pH 13**	-0.63(1)	-0.69(1)	- 0.589(1)
**Peak current ratio**	**pH 0**	0.15(4)		
	**pH 7**	0.19(6)	-	-
	**pH 13**	0.2(2)	0.96(6)	1.2(1)
**Solubility / M**	**1 M H_2SO_4**	< 0.01	< 0.01	< 0.01
	**1 M KCl**			
	**1 M KOH**	0.74-0.62	0.59-0.44	0.05-0.04
**Electrochemical reversibility**		Reversible	Reversible at pH 13	Reversible at pH 13
**Comment**		Double oxidation peak at all pHs	* Electrode reaction	Multiple peaks at all pHs, quinone peak reported<br>*Electrode reaction

Table 4a: Solubility and electrochemistry data collected on benzoquinones (1-4 of 9).

**Structure**		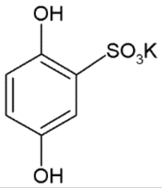	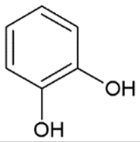	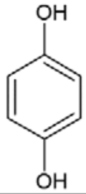	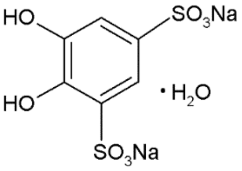
**Name**		2,5-dihydroxybenzenesulfonate potassium	Ortho hydroquinone	Hydroquinone	*Tiron* 4,5-dihydroxy-1,3-benzenedisulfonate disodium monohydrate
**Abbreviation**		HQ(1,4)S	HQ(1,2)	HQ(1,4)	HQ(1,2)DS
**Supplyer**		TCI	Sigma-Aldrich	Sigma-Aldrich	Sigma-Aldrich
**Purity**		96%	99%	99%	97%
**Molarity / M**		228.26	110.11	110.11	332.22
**E'^0 / V_{NHE}**	**pH 0**	0.664(8)	0.783(1)	0.669(5)	0.86(3)
	**pH 7**	0.471(4)	0.464(5)	0.433(7)	0.7*
	**pH 13**	-0.07(1)	0.15*	0.11(1)*	-
**Peak current ratio**	**pH 0**	0.35(6)	2.8(4)	2.3(1)	0.4(1)
	**pH 7**	0.6(3)	2(1)	1.2(3)	-
	**pH 13**	0.09(4)	-	-	
**Solubility / M**	**1 M H_2SO_4**	0.73-0.63	1.8-1.5	0.30-0.29	0.75-0.60
	**1 M KCl**			0.34-0.32	0.30-0.25
	**1 M KOH**	0.88-0.73	0.77-0.70	1.8-1.5	0.60-0.50
**Electrochemical reversibility**		Quasireversible	Quasireversible at pH 0 and 7. Irreversible at pH 13	Quasireversible at pH 0 and 7.	Quasireversible
**Comment**			* Estimated from almost irreversible peak	*From first reduction peak	* Estimated from almost irreversible peak

Table 4b: Solubility and electrochemistry data collected on benzoquinones (5 of 9).

**Structure**		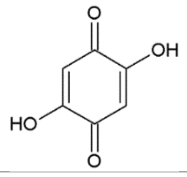	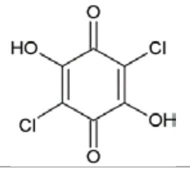	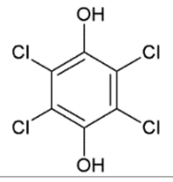	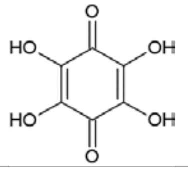	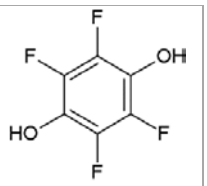
**Name**		2,5-dihydroxy-1,4-benzoquinone	*Chloranilic acid* 2,5-dichloro-3,6-1,4-benzoquinone	Tetrachloro hydroquinone	Tetrahydroxy-1,4-benzoquinone	1,2,4,5-tetrafluoro-3,6-dihydroxybenzene
**Abbreviation**		BQ(1,4)DH	BQ(1,4)DHDCl	HQ(1,4)TCl	BQ(1,4)TH	HQ(1,4)TF
**Supplyer**		TCI	Sigma-Aldrich	Sigma-Aldrich	TCI	TCI
**Purity**		95%	99%	99%	96%	97%
**Molarity / M**		140.09	208.98	245.88	172.09	182.07
**E'^0 / V_{NHE}**	**pH 0**	0.382(4)	0.394(1)	0.70(1)*	0.28	0.707(4)
	**pH 7**	- 0.303(7)	- 0.303(4)	0.277(1)	- 0.21*	0.23(7)
	**pH 13**	- 0.732(2)	- 0.6(1)	0.08(1)**	-	-0.05(6)
**Peak current ratio**	**pH 0**	0.87(4)	1.3(1)	0.72(5)		0.5(1)
	**pH 7**	1.1(2)	1.2(4)	1.1(2)		0.21(7)
	**pH 13**	0.8(1)	0.8(2)	-	-	0.2(1)
**Solubility / M**	**1 M H_2SO_4**		< 0.01	< 0.01		0.69-0.61
	**1 M KCl**	< 0.01	0.01	0.01		
	**1 M KOH**	0.51-0.48	< 0.01	< 0.01	< 0.01	1.10-0.92
**Electrochemical reversibility**		Quasireversible	Reversible at pH 0, Quasireversble at pH 7 & 13	Quasireversible	Quasireversible at pH 6.2 & 13	Quasireversible
**Comment**				* double oxidation peak at low scan rates **from estimated reduction peak around -0.25 V_{NHE}	*at pH 6.2	

S5: Collected data on nitrogen containing compounds

**Structure**		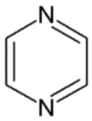	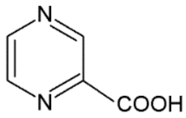	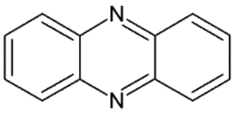	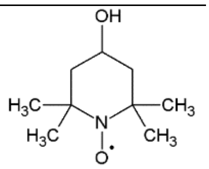
**Name**		Pyrazine	Pyrazine-2-carboxylic acid	Phenazine	TEMPOL
**Supplyer**		Sigma-Aldrich	Sigma-Aldrich	Sigma-Aldrich	TCI
**Purity**		99 %	99 %	98 %	98 %
**$E^{0´}$ / V_{NHE}**	pH = 0<br>pH = 7<br>pH = 13	-0.013(2)<br>Unstable	-0.013(3)<br>Unstable	0.403(3)<br>Unstable	0.73(5)<br>0.803(2)<br>Unstable
**Solubility / M**	1 M H_2SO_4<br>1 M KCl<br>1 M KOH	≈ 7 (forms azeotrope with water)	0.07<br>0.08<br>0.9	0.01<br>< 0.01	2.5<br>1.5<br>1.2

S6: CV data of organic species

In cases where data are only shown for certain pH values, tests in other pH values yielded no reasonable electrochemical response.

Contents

AQDS(2,7): 9,10-anthraquinone-2,7-disulfonate disodium 16
AQDS(1,5): 9,10-anthraquinone-1,5-disulfonate disodium 17
AQDS(1,8): 9,10-anthraquinone-1,8-disulfonate dipotassium........ 18
AQS(2): 9,10-anthraquinone-2-sulfonate sodium 19
AQS(2)DH (Alizarin red s): 3,4-dihydroxy-9,10-anthraquinone-2-sulfonate sodium........ 20
AQS(2)NBr: 1-amino-4-bromo-9,10-anthraquinone-2-sulfonate sodium........ 21
NQ(1,4)HB (Lapachol): 2-hydroxy-3-(3-methyl-2-butenyl)-1,4-naphthoquinone 23
NQ(1,4)H: 2-hydroxy-1,4-naphtoquinone........ 24
NQ(1,4)DHCl: 2,3-dichloro-5,8-dihydroxy-1,4-naphtoquinone 25
AQDH(2,6) (Anthraflavic acid): 2,6-dihydroxy-9,10-anthraquinone........ 26
AQDH(1,8) (Chrysazin): 1,8-dihydroxy-9,10-anthraquinone 27
AQDH(1,5) (Anthrarufin): 1,5-dihydroxy-9,10-anthraquinone........ 28
AQTH(1,2) (Quinalizarin): 1,2,5,8-tetrahydroxy-9,10-anthraquinone 29
AQDH(1,2) (Alizarin): 1,2-dihydroxy-9,10-anthraquinone 30
AQDH(1,4) (Leucoquinizarin): 2,3-dihydro-9,10-dihydroxy-1,4-anthraquinone........ 31
AQDH(1,8)MH (Aloemodin): 1,8-dihydroxy-3-(hydroxymethyl)-9,10-anthraquinone........ 32
AQTrHM (*Emodin)*: 1,3,8-trihydroxy-6-methylanthracene-9,10-dione........ 33
AQTH(1,4): 1,4,5,8-tetrahydroxy-9,10-anthraquinone 34
AQDH(1,8)CA (Rhein): 4,5-dihydroxy-9,10-anthraquinone-2-carboxylic acid........ 35
HQ(1,4)S: 2,5-dihydroxybenzenesulfonate potassium........ 36
HQ(1,2): Ortho hydroquinone 37
HQ(1,4): Para hydroquinone........ 38
HQ(1,2)DS (Tiron): 4,5-dihydroxy-1,3-benzenedisulfonate disodium monohydrate........ 39
HQ(1,4)DH: 2,5-dihydroxy-1,4-benzoquinone........ 40
BQ(1,4)DHDCl (Chloranilic acid): 2,5-dichloro-3,6-1,4-benzoquinone........ 41
HQ(1,4)TCl: Tetrachloro hydroquinone........ 42
BQ(1,4)TH: Tetrahydroxy-1,4-benzoquinone........ 43
HQ(1,4)TF: 1,2,4,5-tetrafluoro-3,6-dihydroxybenzene 44
Pyrazine: 1,4-diazine 45
Pyrazine-2-carboxylic acid........ 46
Phenazine........ 47
TEMPOL: 4-hydroxy-2,2,6,6-tetramethylpiperidine-1-oxyl 48

AQDS(2,7): 9,10-anthraquinone-2,7-disulfonate disodium

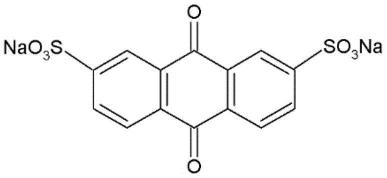

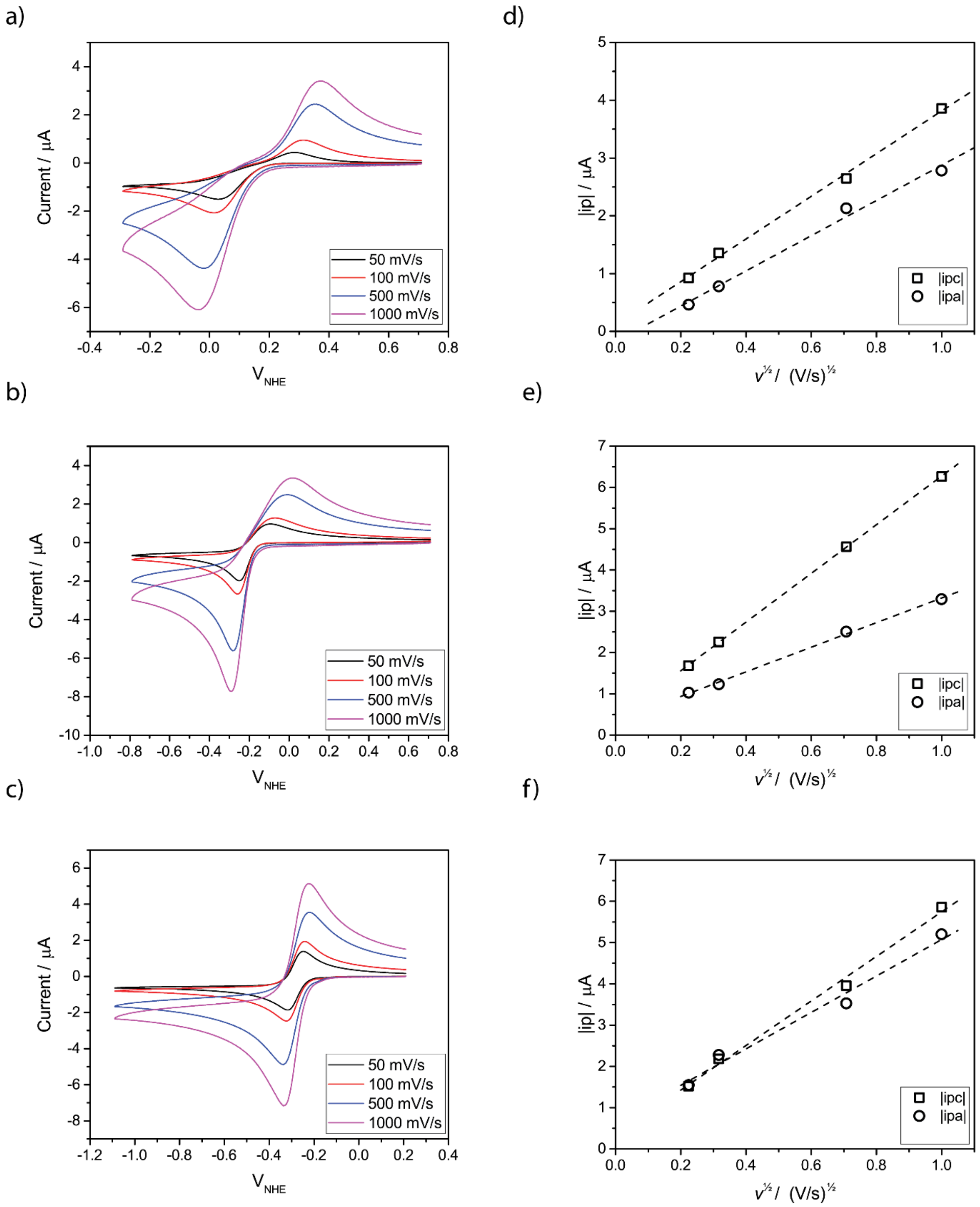

Figure 5: CV and ip vs. v plot for AQDS(2,7) a)+d) pH 0, b)+e) pH 7 and c)+f) pH 13.

AQDS(1,5): 9,10-anthraquinone-1,5-disulfonate disodium

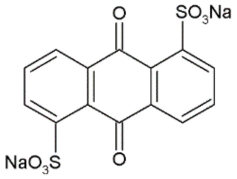

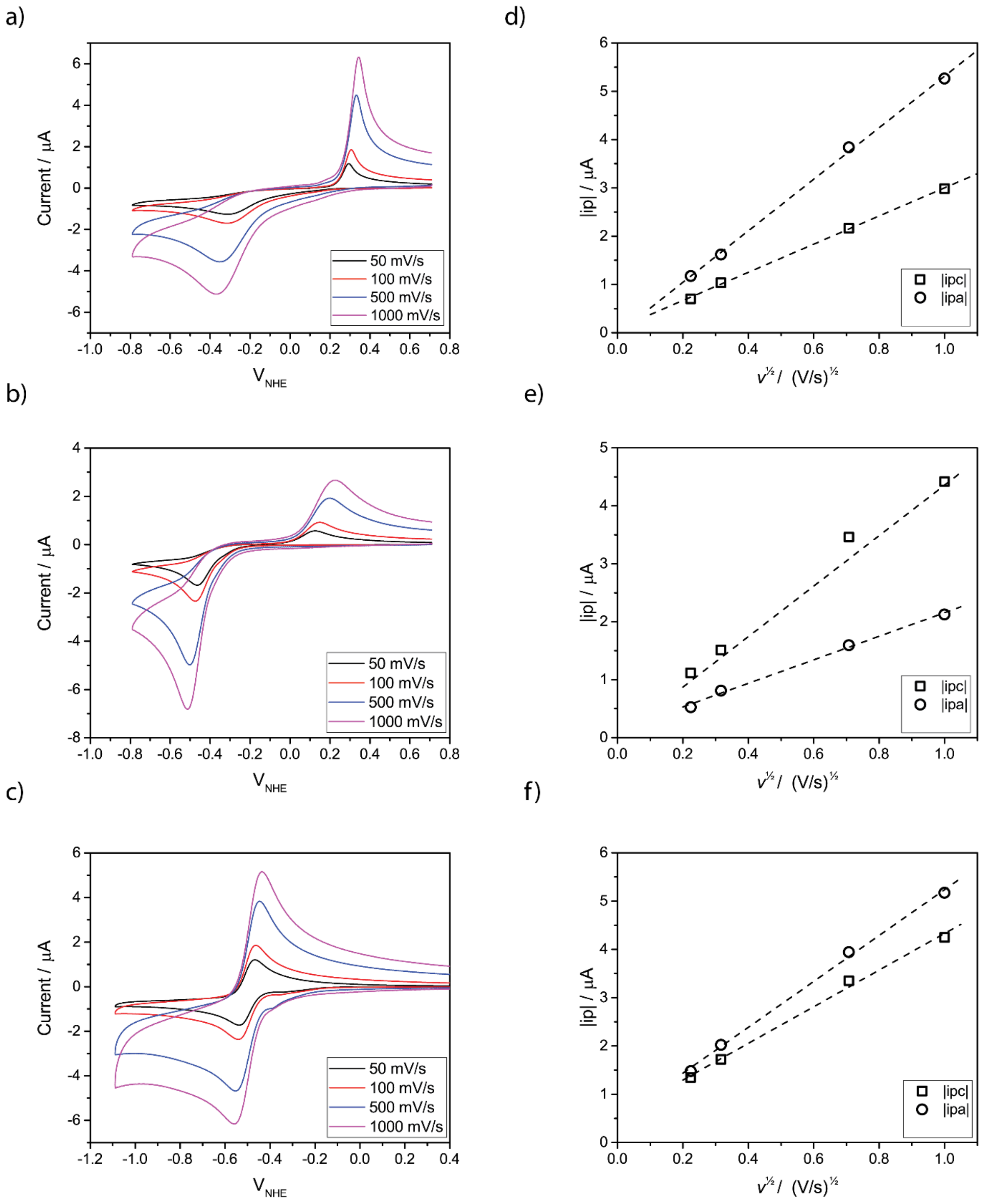

Figure 6: CV and ip vs. v plot for AQDS(1,5) a)+d) pH 0, b)+e) pH 7 and c)+f) pH 13.

AQDS(1,8): 9,10-anthraquinone-1,8-disulfonate dipotassium

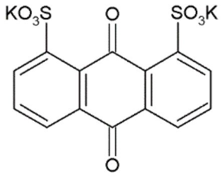

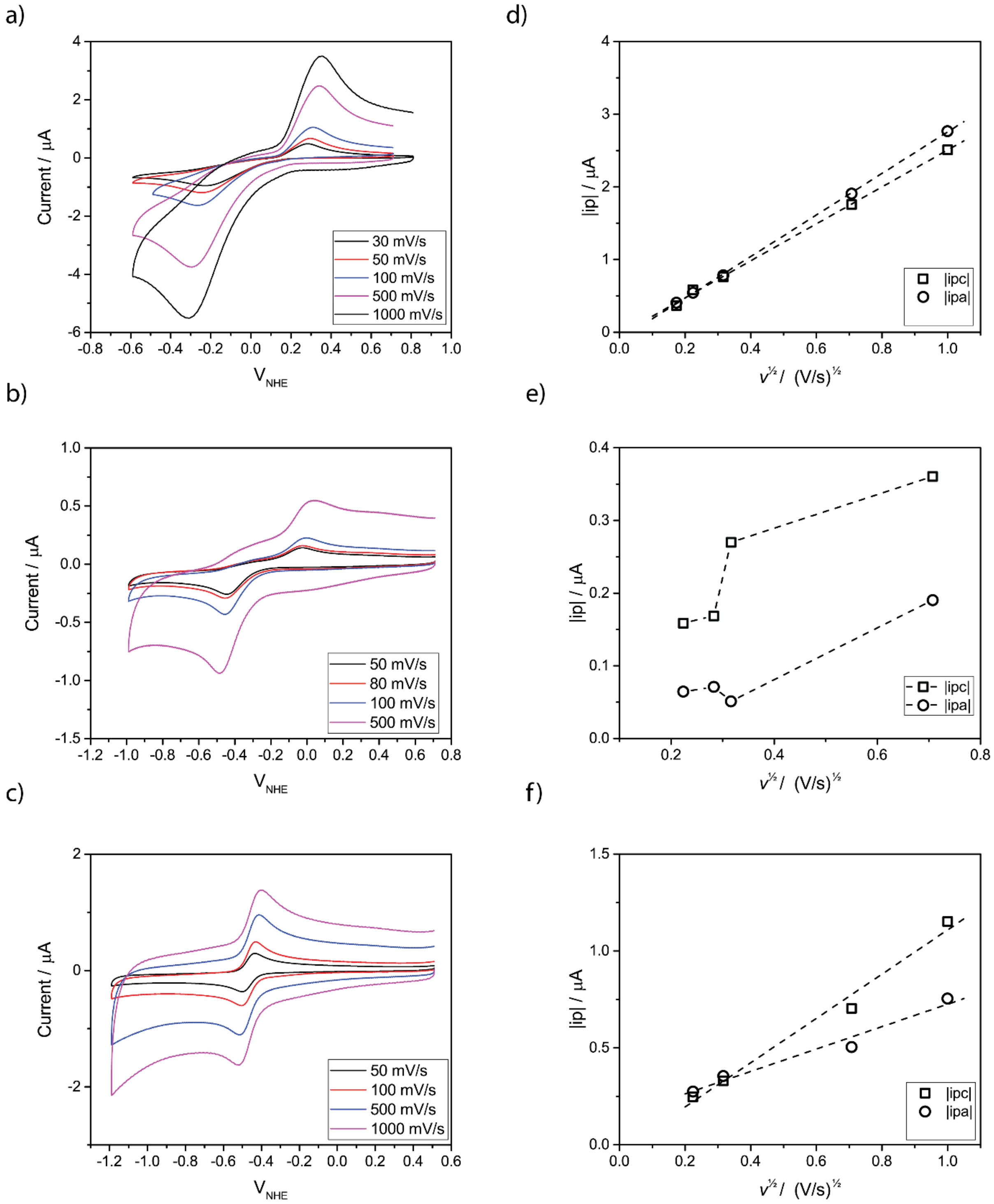

Figure 7: CV and ip vs. v plot for AQDS(1,8) a)+d) pH 0, b)+e) pH 7 and c)+f) pH 13.

AQS(2): 9,10-anthraquinone-2-sulfonate sodium

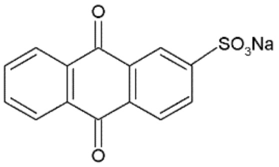

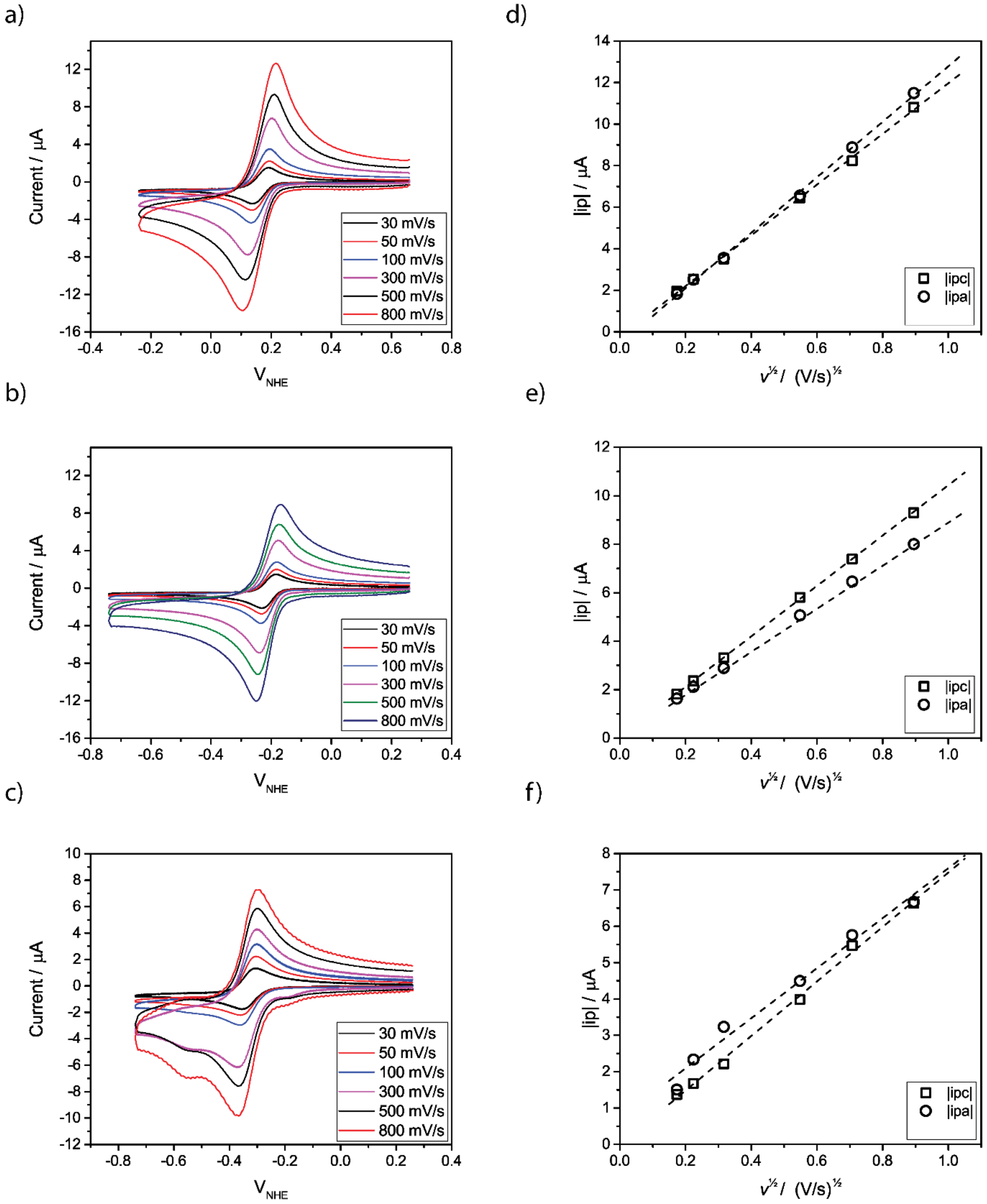

Figure 8: CV and ip vs. v plot for AQS(2) a)+d) pH 0, b)+e) pH 7 and c)+f) pH 13.

AQS(2)DH (Alizarin red s): 3,4-dihydroxy-9,10-anthraquinone-2-sulfonate sodium

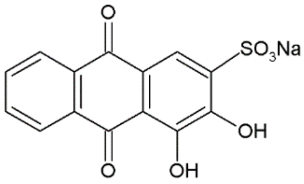

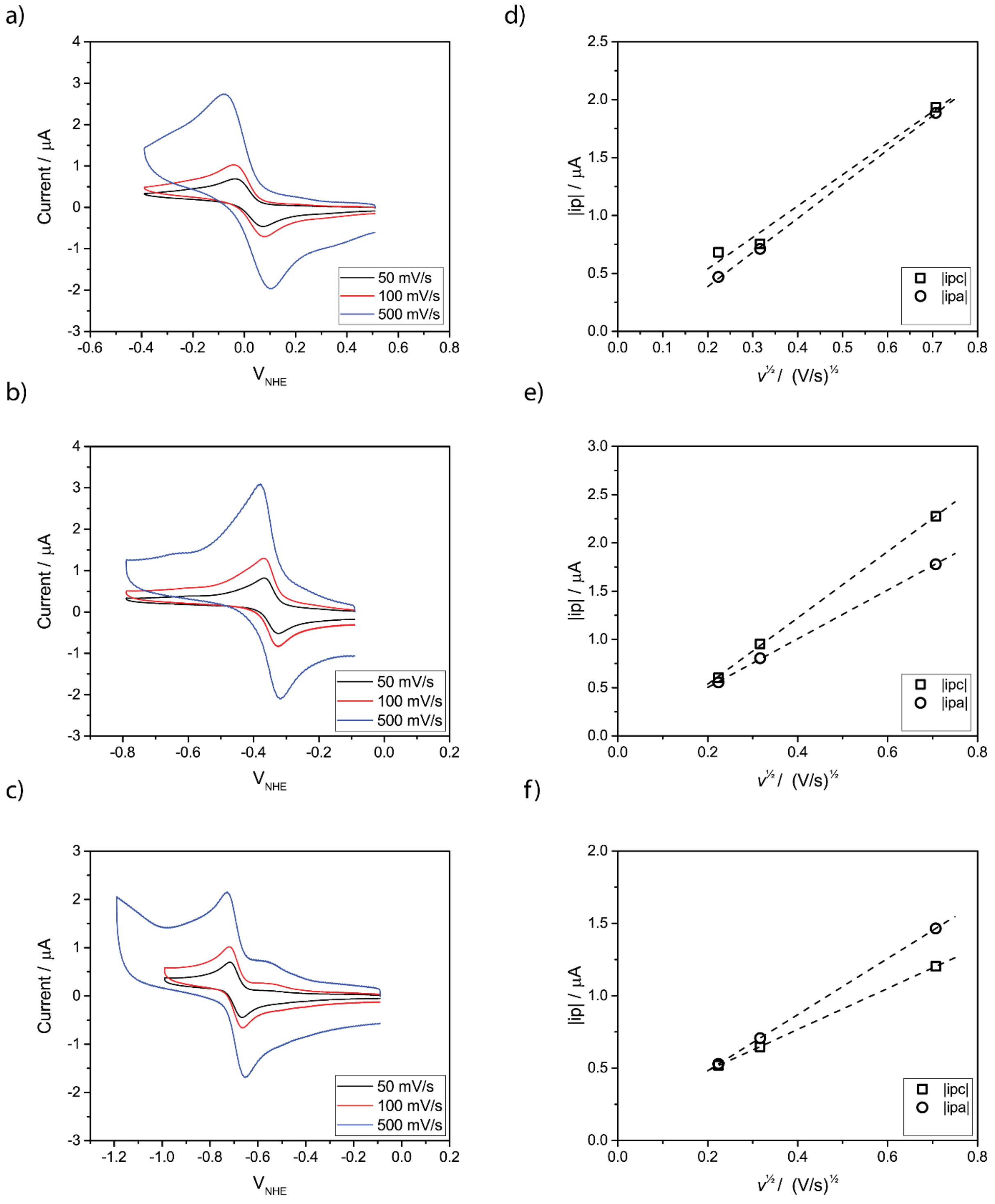

Figure 9: CV and ip vs. v plot for AQS(2)DH a)+d) pH 0, b)+e) pH 7 and c)+f) pH 13.

AQS(2)NBr: 1-amino-4-bromo-9,10-anthraquinone-2-sulfonate sodium

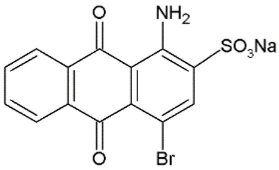

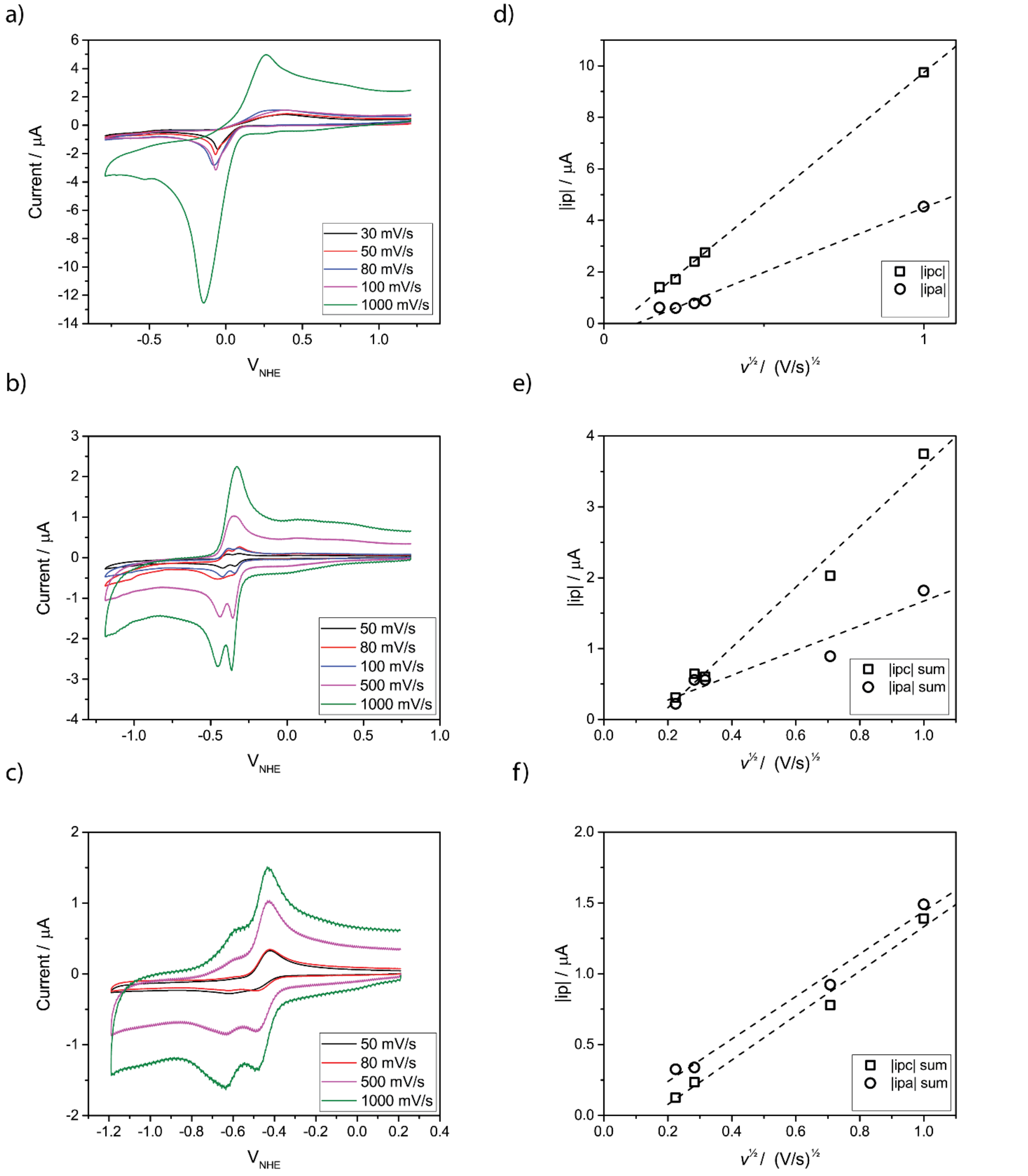

Figure 10: CV and ip vs. v plot for AQS(2)NBr a)+d) pH 0, b) + e) pH 7 and c)+f) pH 13. For double peaks, the plotted ip is the sum of both ips.

NQ(1,2)S: 1,2-naphtoquinone-4-sulfonate sodium

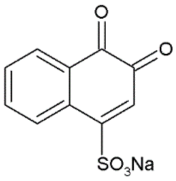

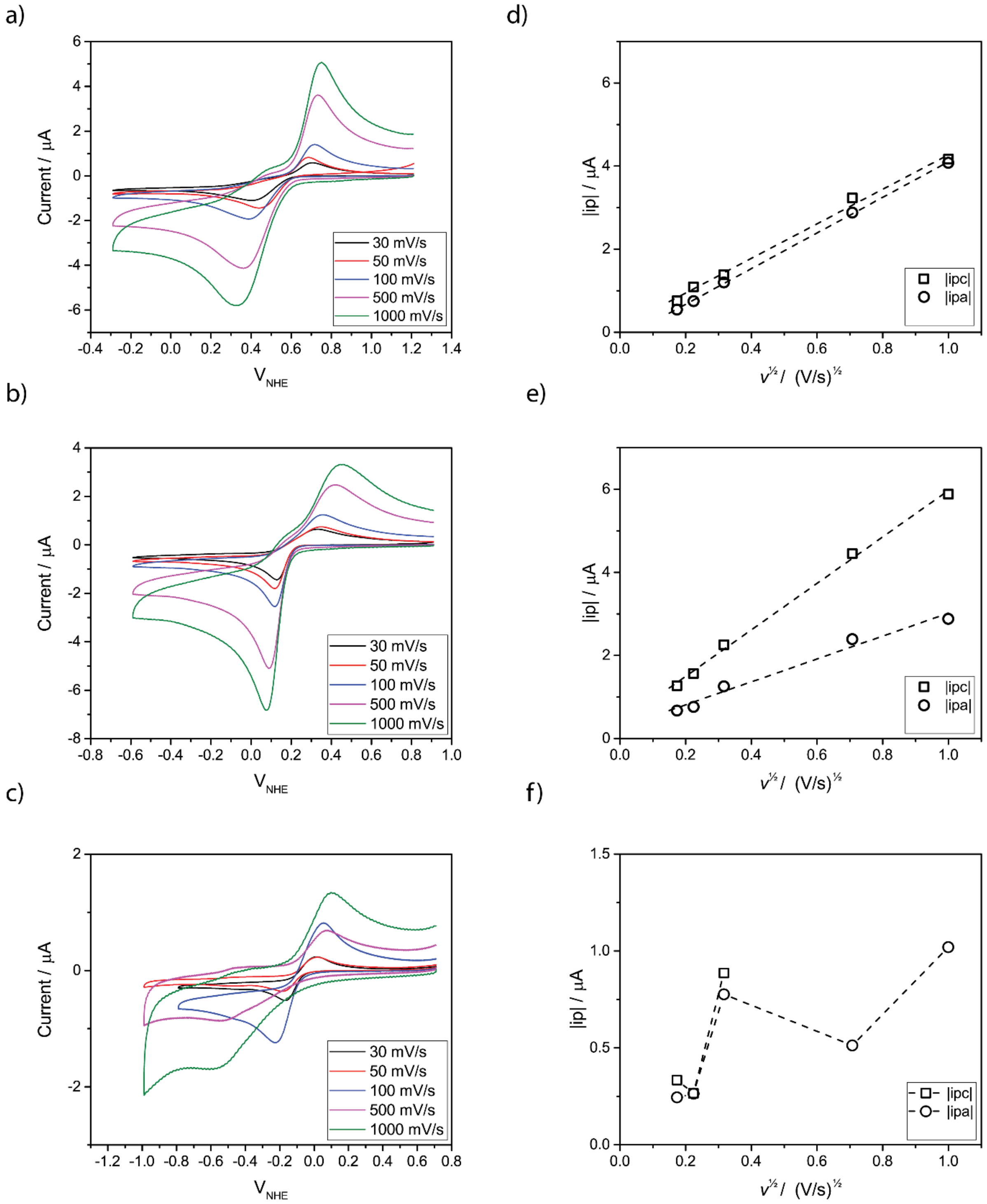

Figure 11: CV and ip vs. v plot for NQ(1,2)S a)+d) pH 0, b)+e) pH 7 and c)+f) pH 13.

NQ(1,4)HB (Lapachol): 2-hydroxy-3-(3-methyl-2-butenyl)-1,4-naphthoquinone

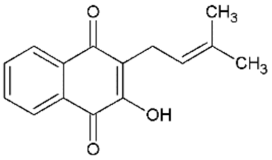

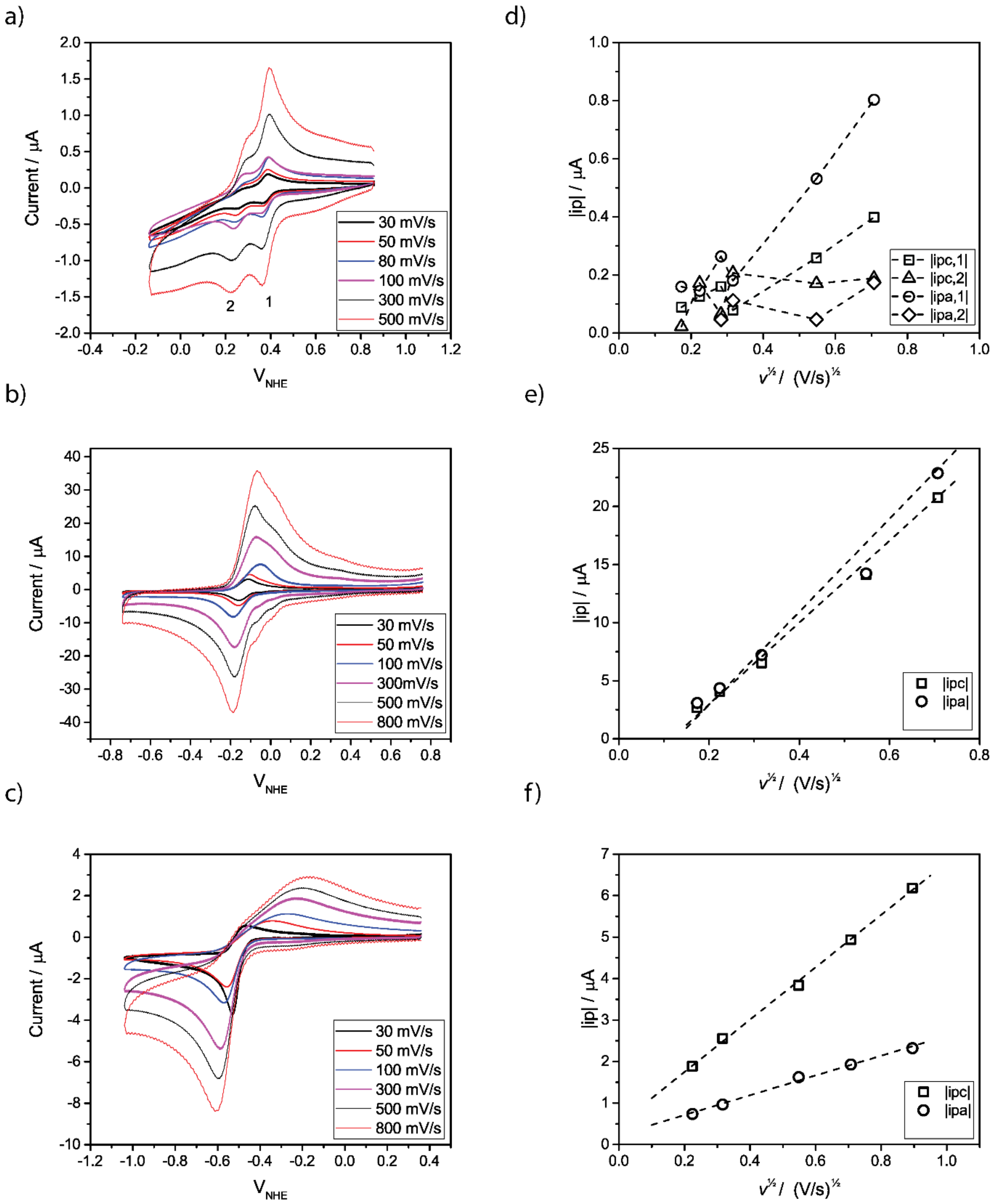

Figure 12: CV and ip vs. v plot for NQ(1,4)HB a)+d) pH 0, b)+e) pH 7 and c)+f) pH 13.

NQ(1,4)H: 2-hydroxy-1,4-naphtoquinone

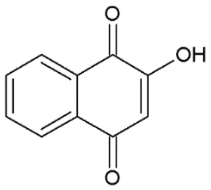

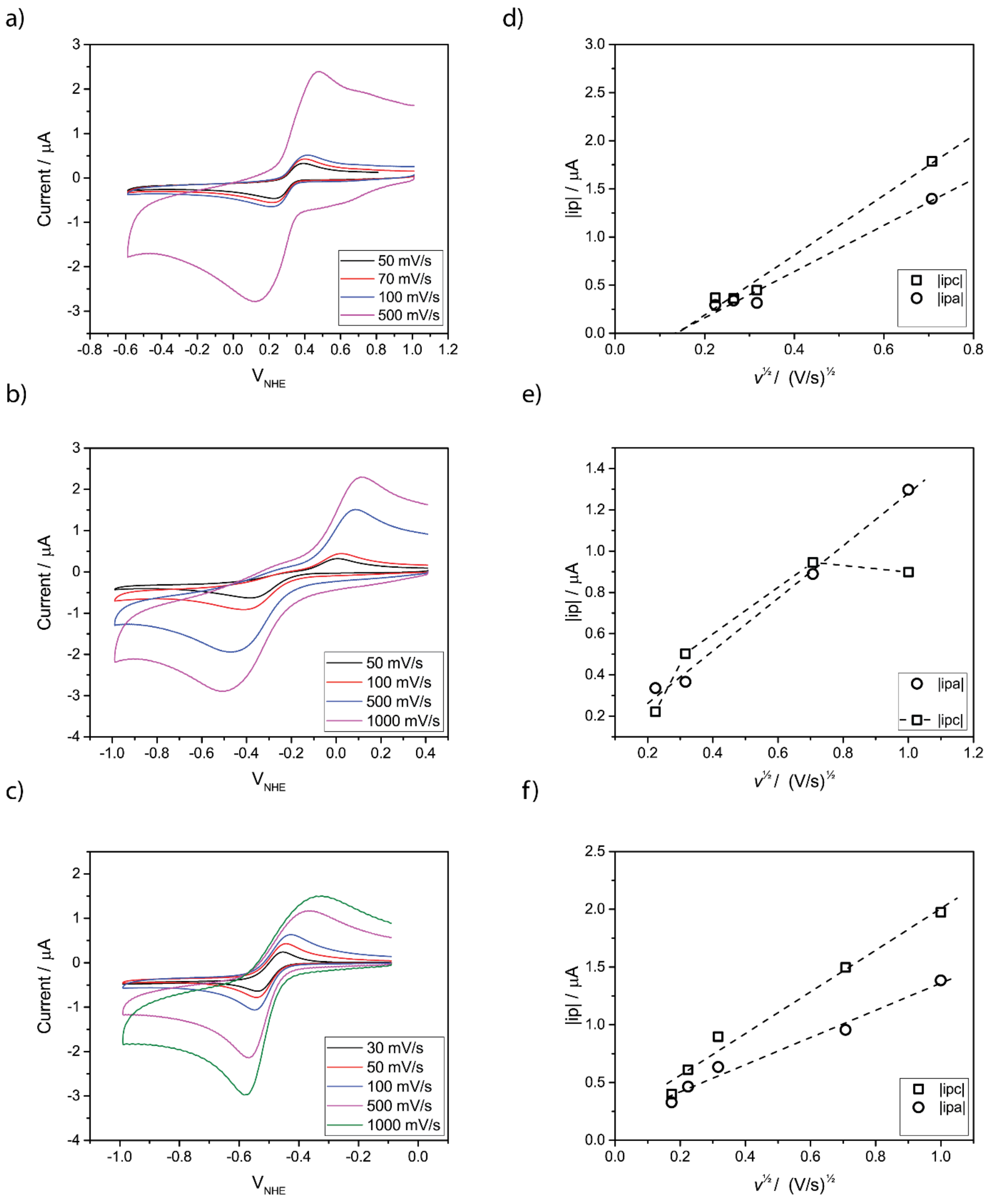

Figure 13: CV and ip vs. v plot for NQ(1,4)H a)+d) pH 0, b)+e) pH 7 and c)+f) pH 13.

NQ(1,4)DHCl: 2,3-dichloro-5,8-dihydroxy-1,4-naphtoquinone

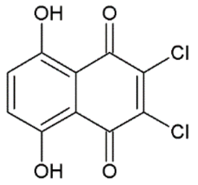

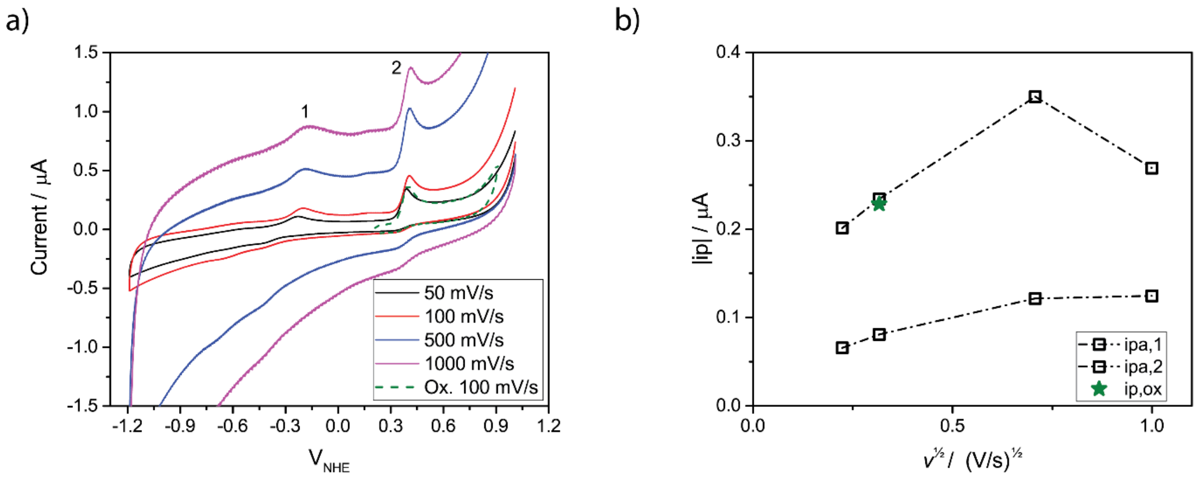

Figure 14: a) CVs and b) ip vs. v plot for NQ(1,4)DHDCl at pH 13.

AQDH(2,6) (Anthraflavic acid): 2,6-dihydroxy-9,10-anthraquinone

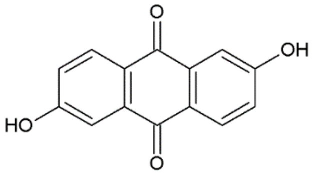

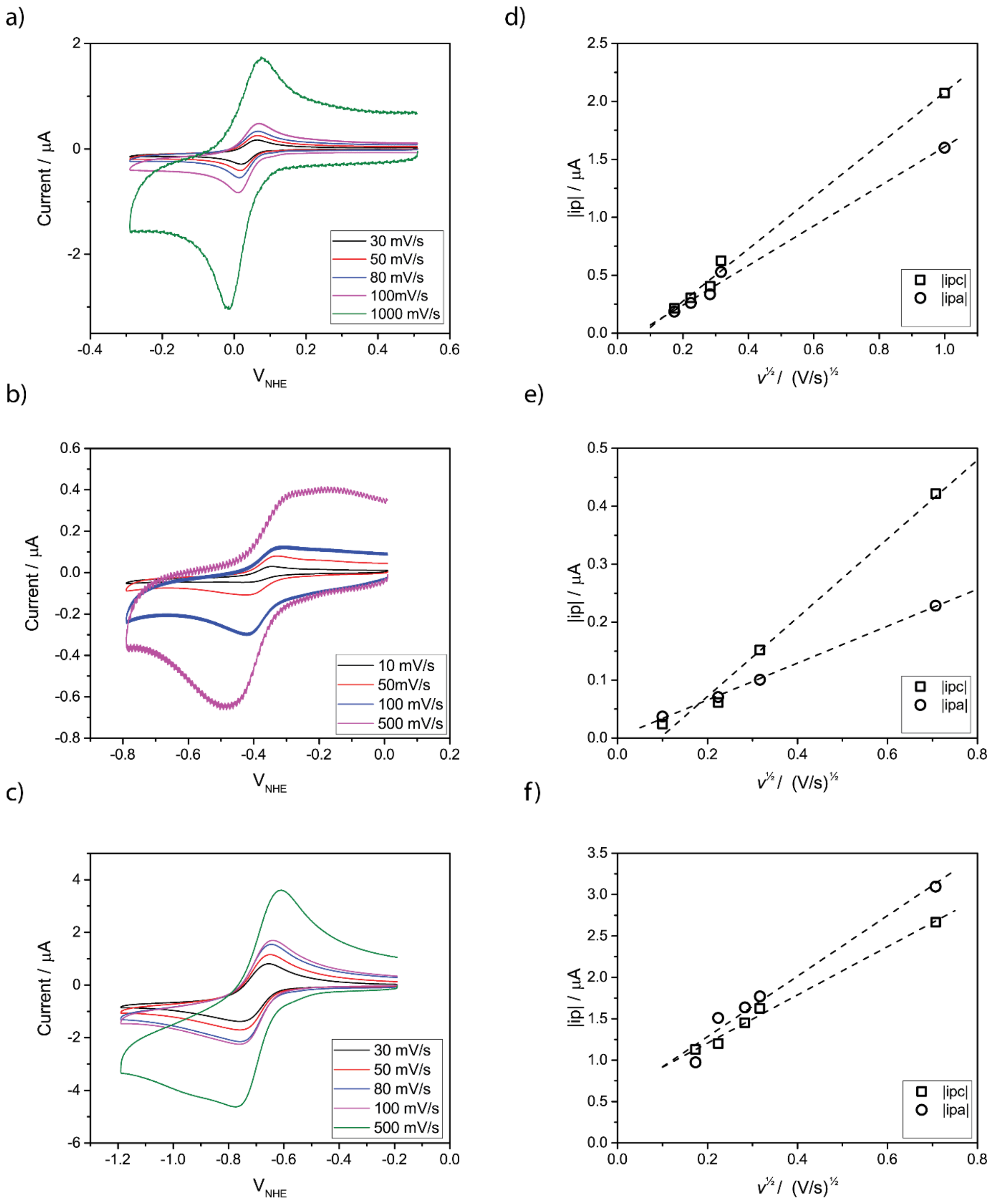

Figure 15: CV and ip vs. v plot for AQDH(2,6) a)+d) pH 0, b)+e) pH 7 and c)+f) pH 13.

AQDH(1,8) (Chrysazin): 1,8-dihydroxy-9,10-anthraquinone

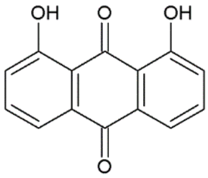

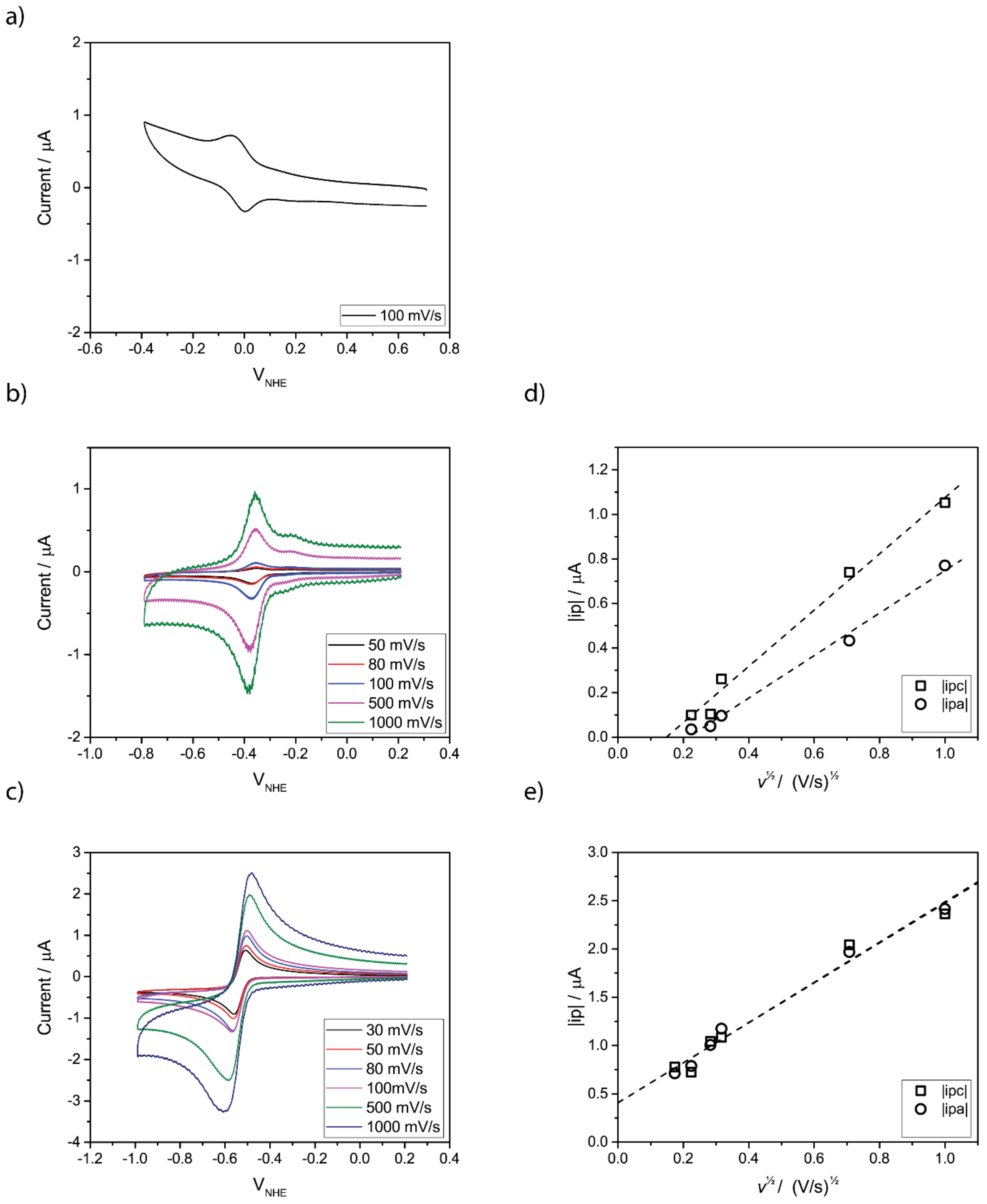

Figure 16: CV and ip vs. v plot for AQDH(1,8) a) pH 0, b) + d) pH 7 and c)+e) pH 13.

AQDH(1,5) (Anthrarufin): 1,5-dihydroxy-9,10-anthraquinone

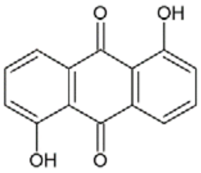

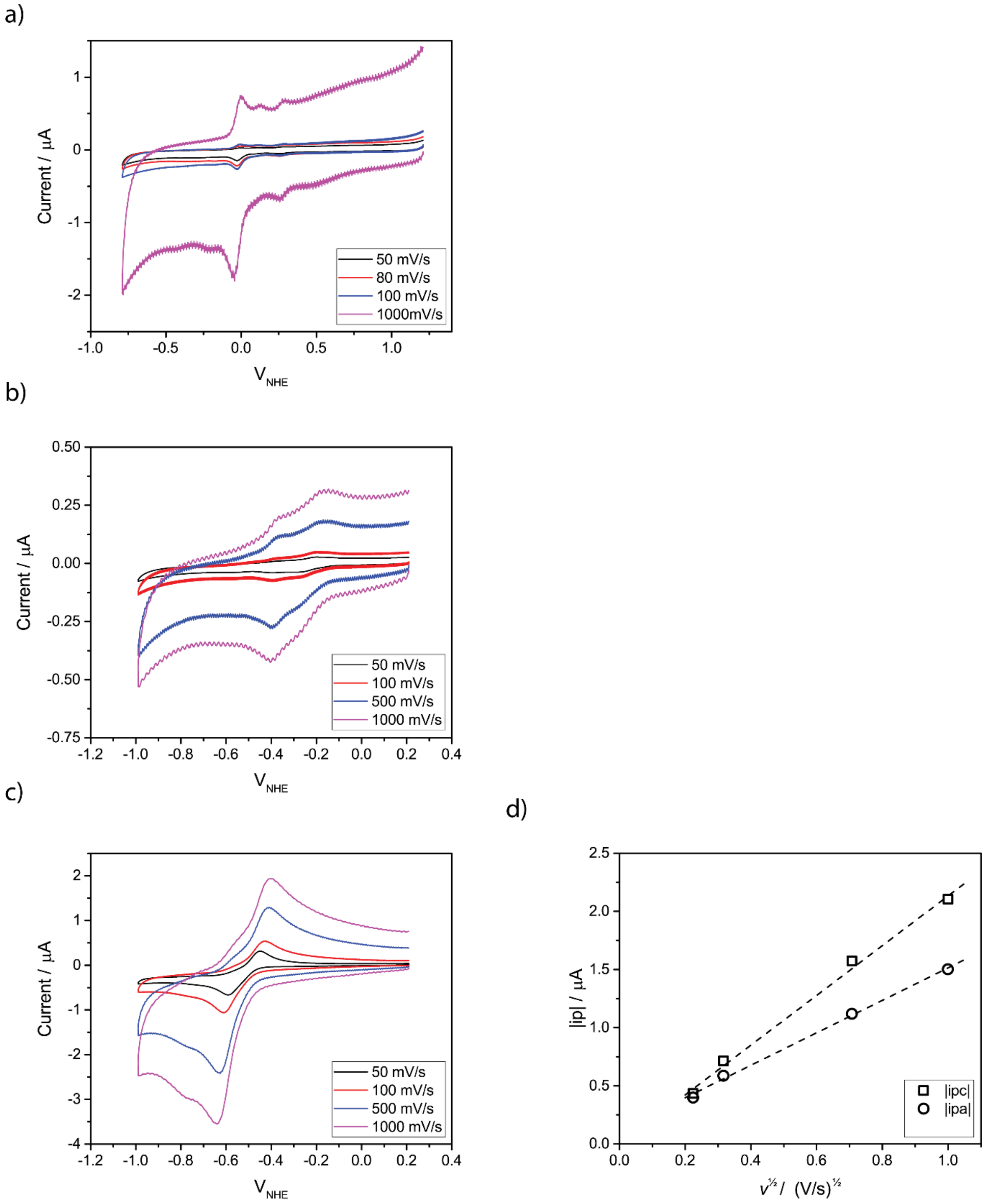

Figure 17: CV for AQDH(1,5) a) pH 0, b) pH 7 and c)+d) pH 13 and ip vs. v plot.

AQTH(1,2) (Quinalizarin): 1,2,5,8-tetrahydroxy-9,10-anthraquinone

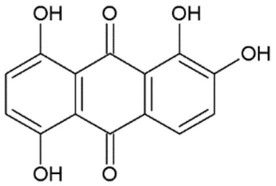

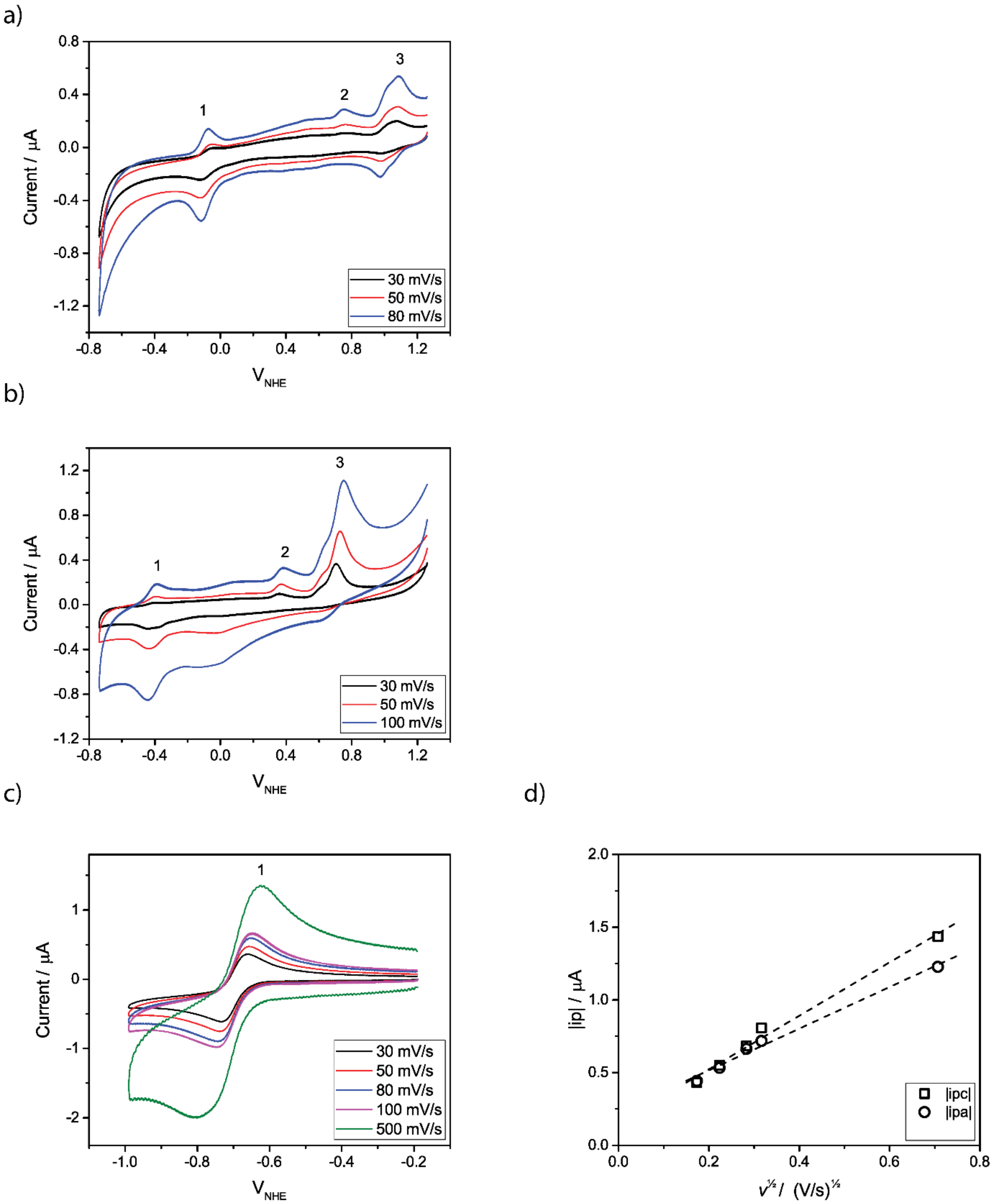

Figure 18: CV for AQTH(1,2) a) pH 0, b) pH 7 and c)+d) pH 13 and ip vs. v plot.

AQDH(1,2) (Alizarin): 1,2-dihydroxy-9,10-anthraquinone

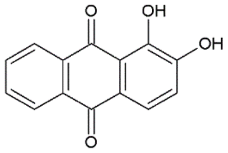

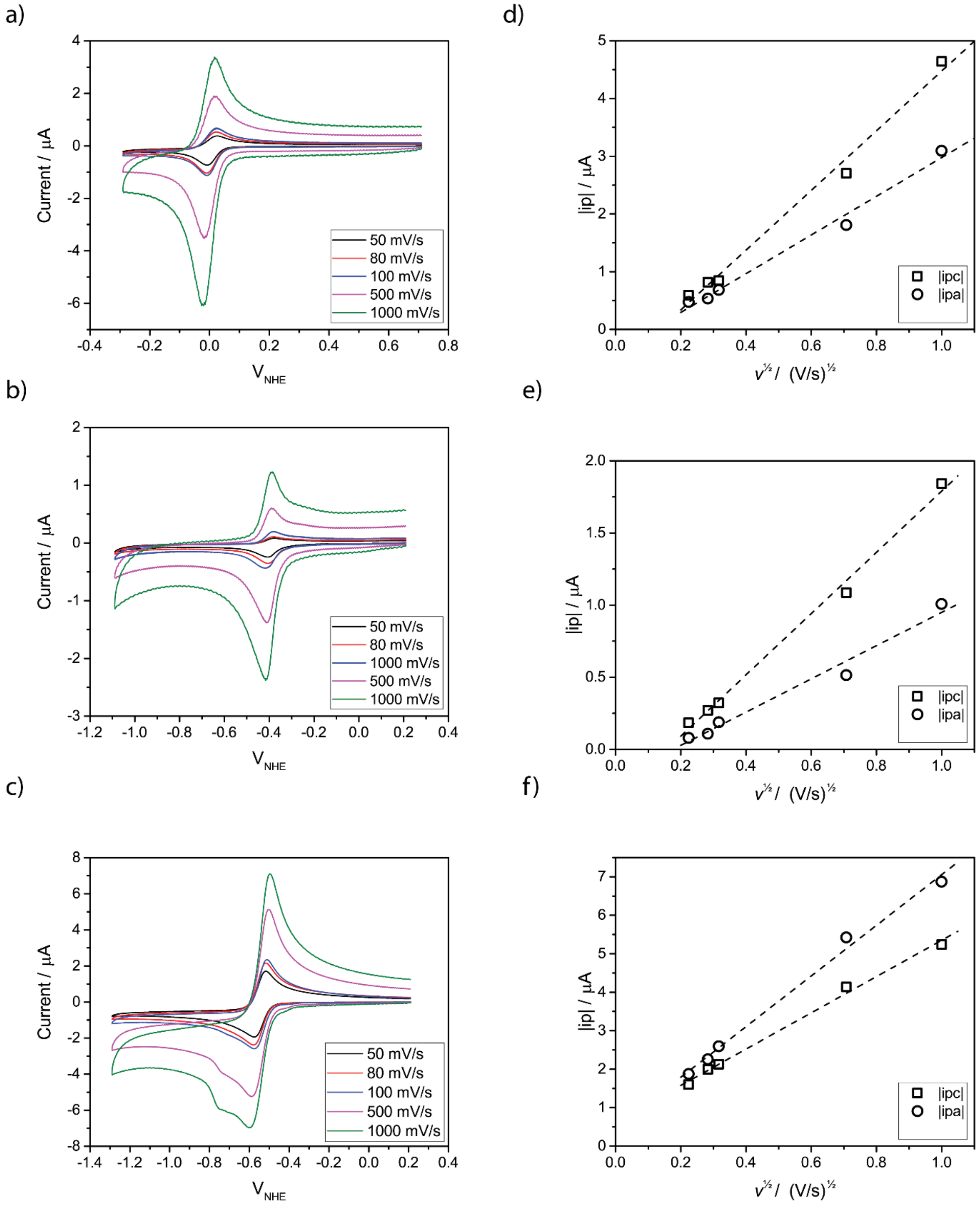

Figure 19: CV and ip vs. v plot for AQDh(1,2) a)+d) pH 0, b)+e) pH 7 and c)+f) pH 13.

AQDH(1,4) (Leucoquinizarin): 2,3-dihydro-9,10-dihydroxy-1,4-anthraquinone

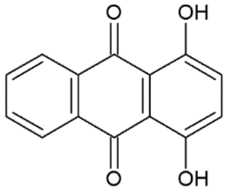

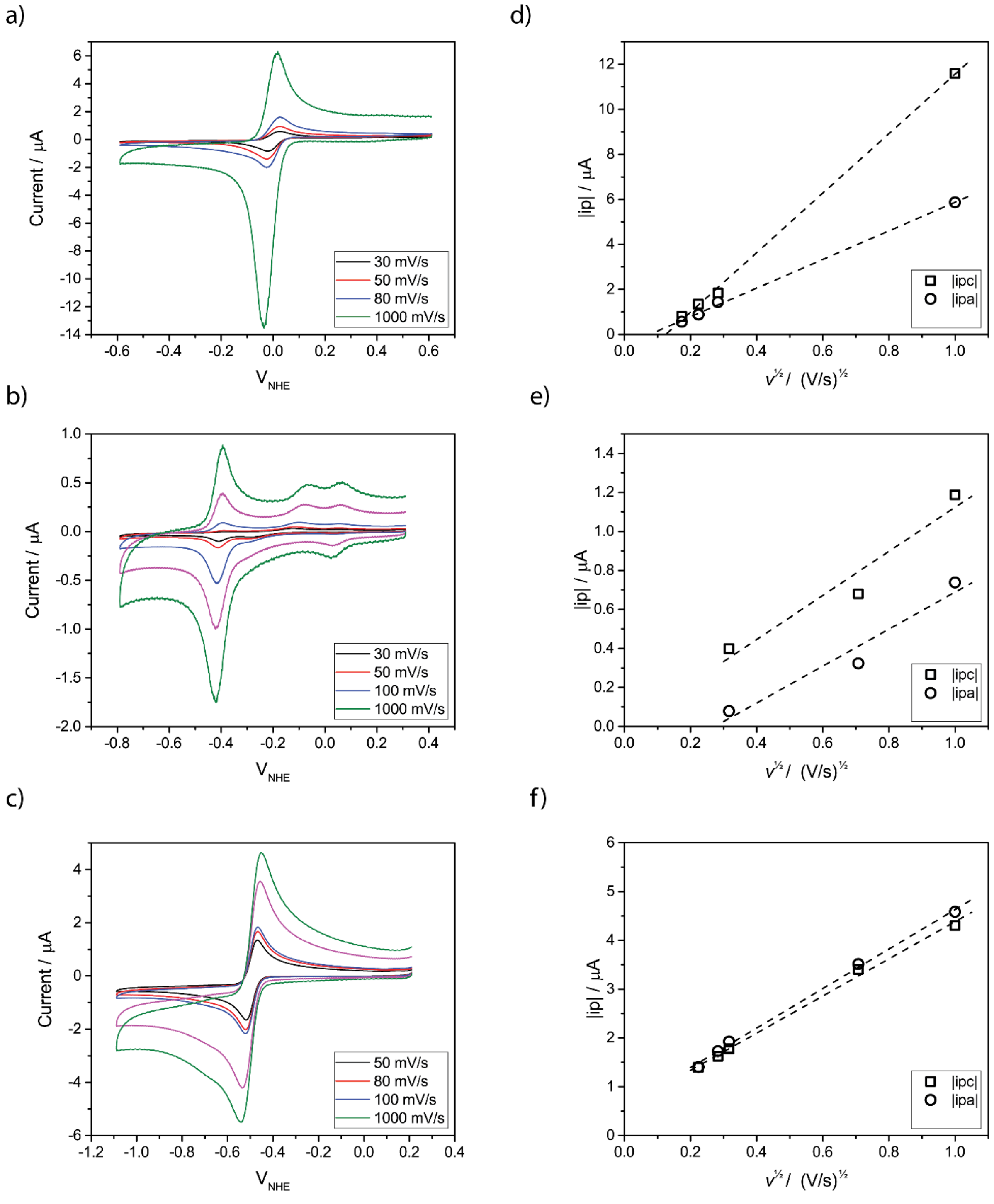

Figure 20: CV and ip vs. v plot for AQDH(1,4) a)+d) pH 0, b)+e) pH 7 and c)+f) pH 13.

AQDH(1,8)MH (Aloemodin): 1,8-dihydroxy-3-(hydroxymethyl)-9,10-anthraquinone

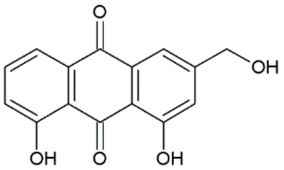

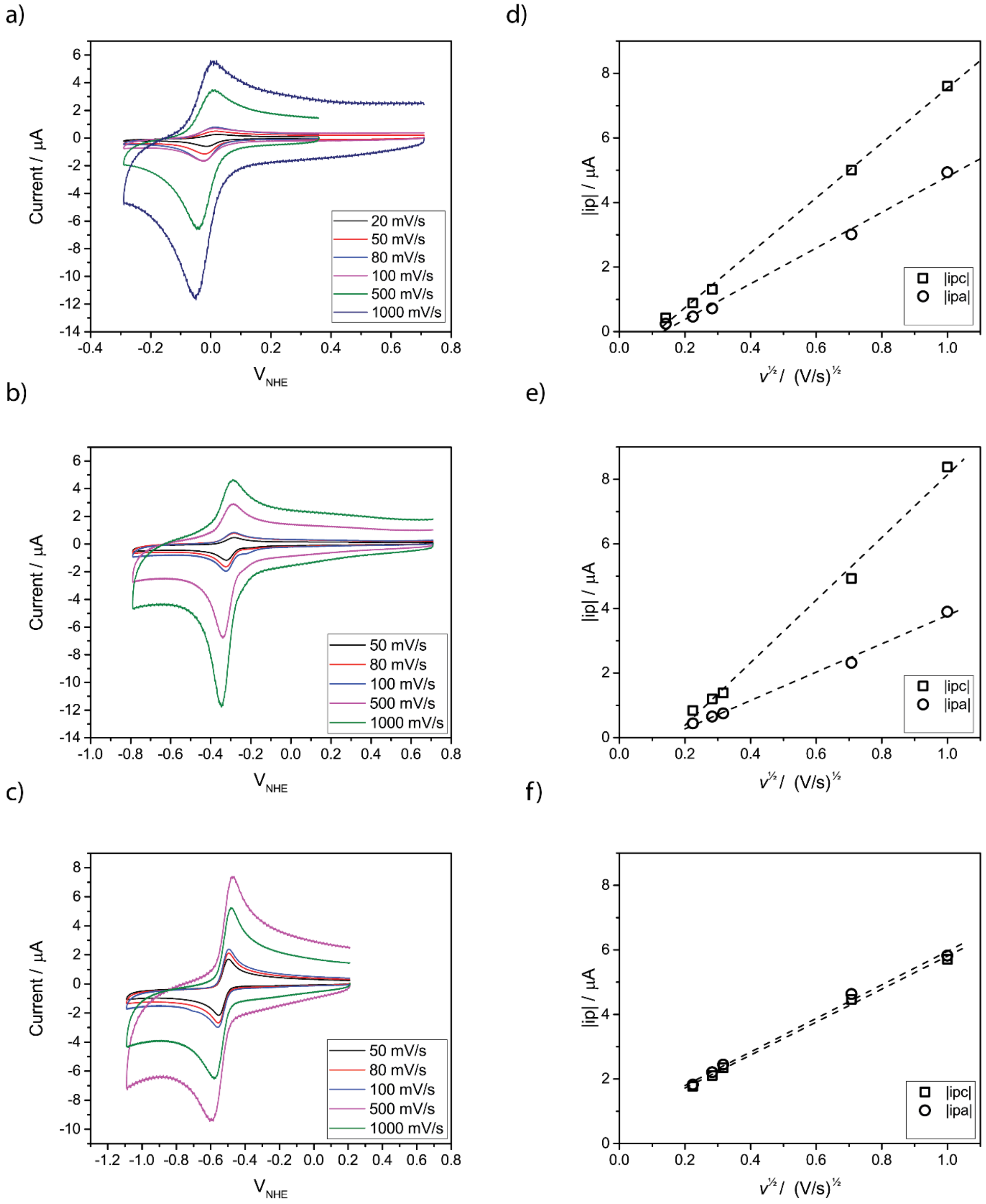

Figure 21: CV and ip vs. v plot for AQDH(1,8)MH a)+d) pH 0, b) + e) pH 7 and c)+f) pH 13.

AQTrHM (*Emodin*): 1,3,8-trihydroxy-6-methylanthracene-9,10-dione

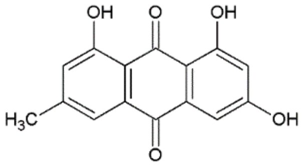

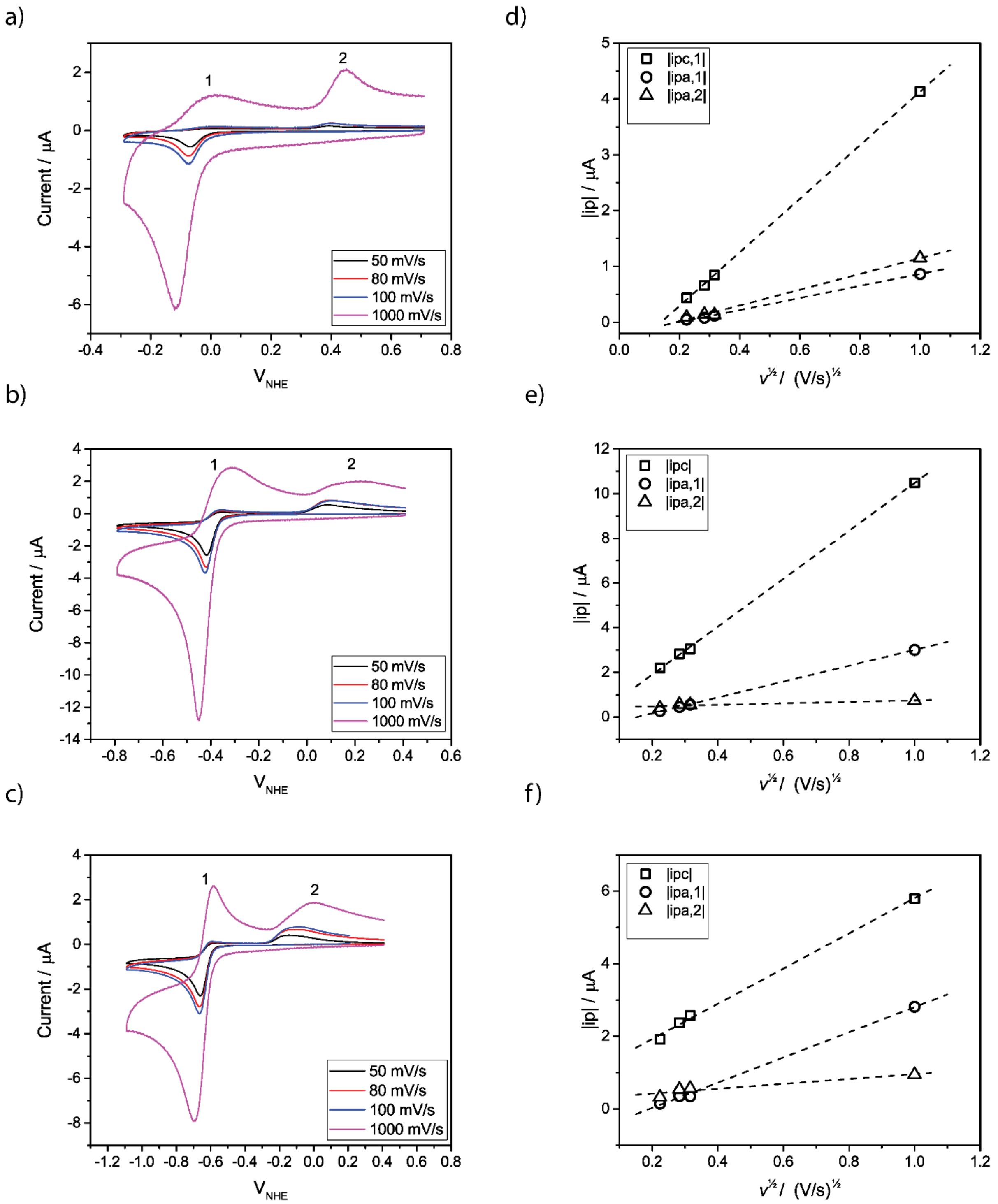

Figure 22: CV and ip vs. v plot for AQTrHM a)+d) pH 0, b) + e) pH 7 and c)+f) pH 13.

AQTH(1,4): 1,4,5,8-tetrahydroxy-9,10-anthraquinone

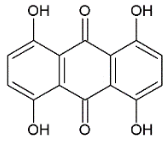

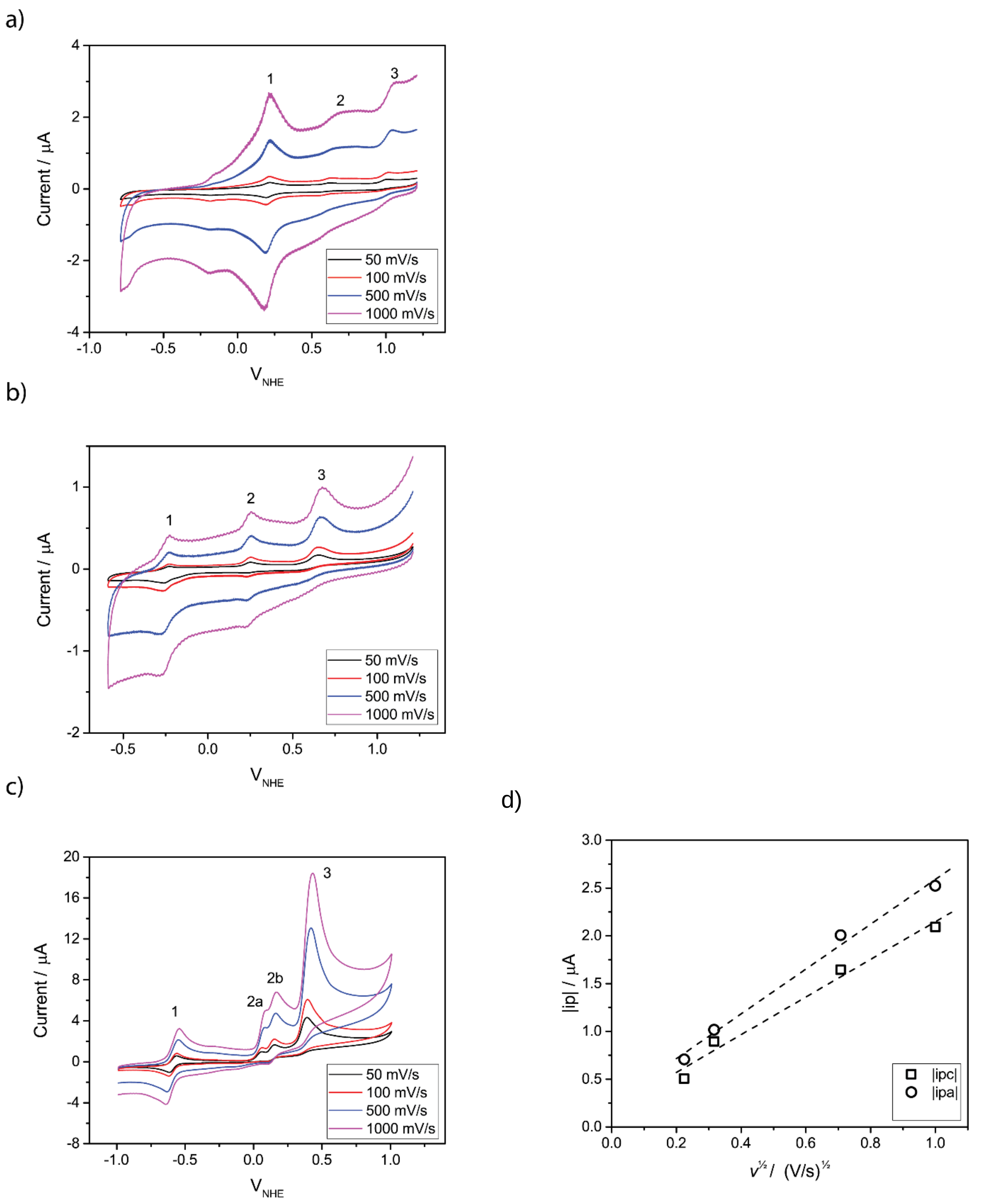

Figure 23: CV for AQTH(1,4) a) pH 0, b) pH 7 and c) pH 13 and d) corresponding ip vs. v plot.

AQDH(1,8)CA (Rhein): 4,5-dihydroxy-9,10-anthraquinone-2-carboxylic acid

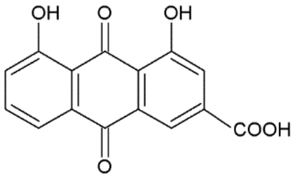

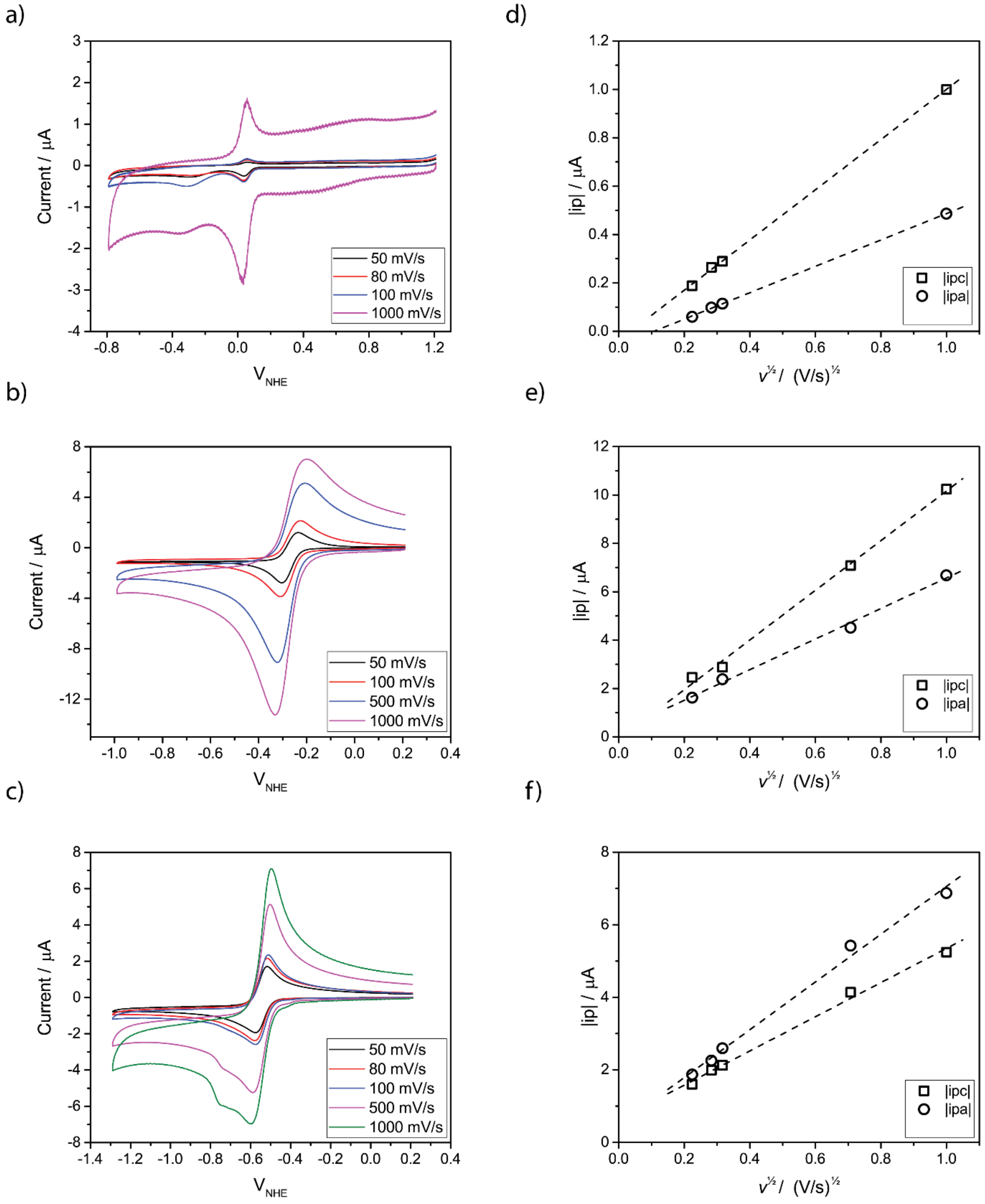

Figure 24: CV and ip vs. v plot for AQDH(1,8)CA a)+d) pH 0, b)+e) pH 7 and c)+f) pH 13.

HQ(1,4)S: 2,5-dihydroxybenzenesulfonate potassium

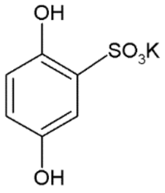

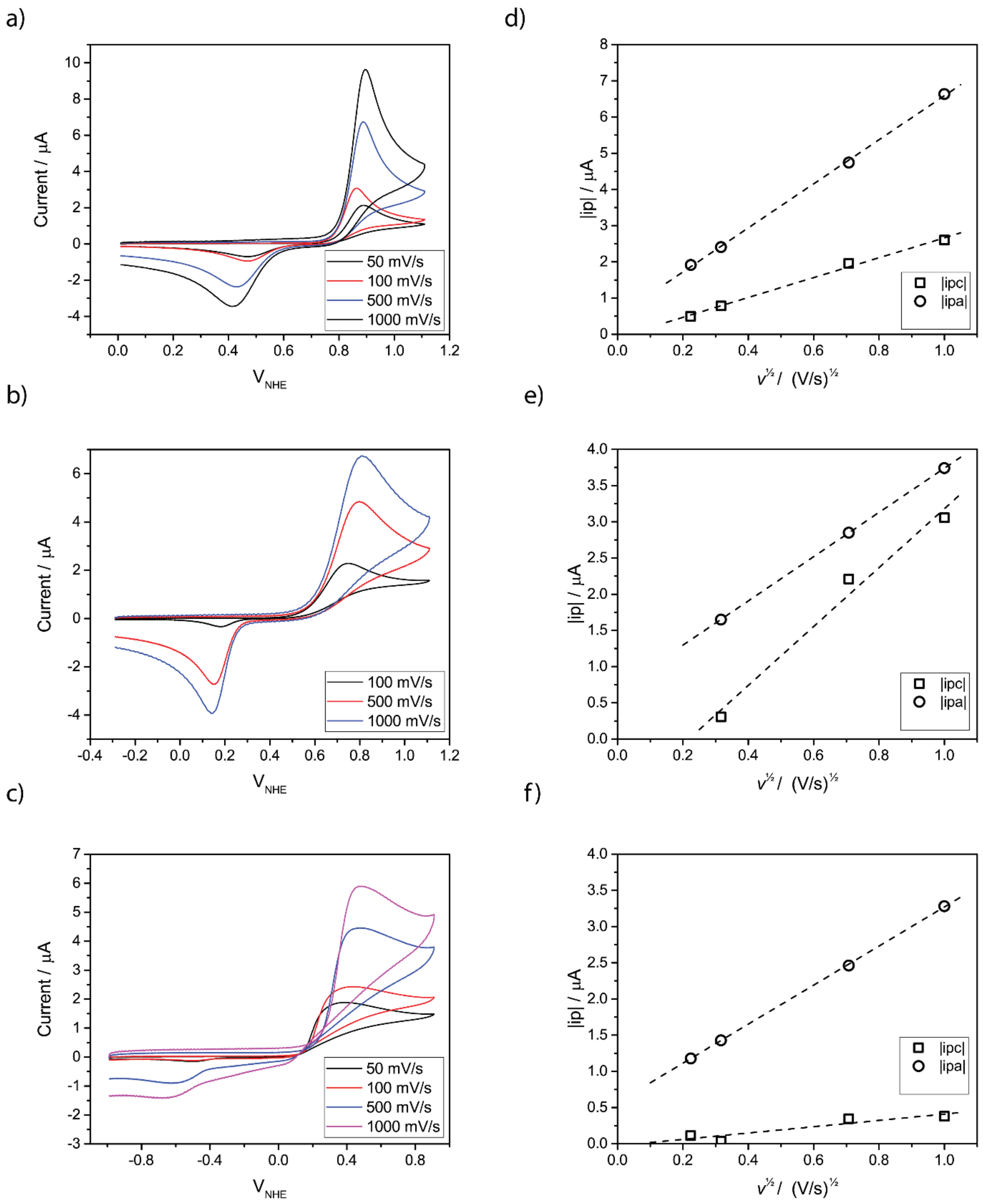

Figure 25: CV and ip vs. v plot for HQ(1,4)S a)+d) pH 0, b)+e) pH 7 and c)+f) pH 13.

HQ(1,2): Ortho hydroquinone

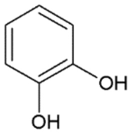

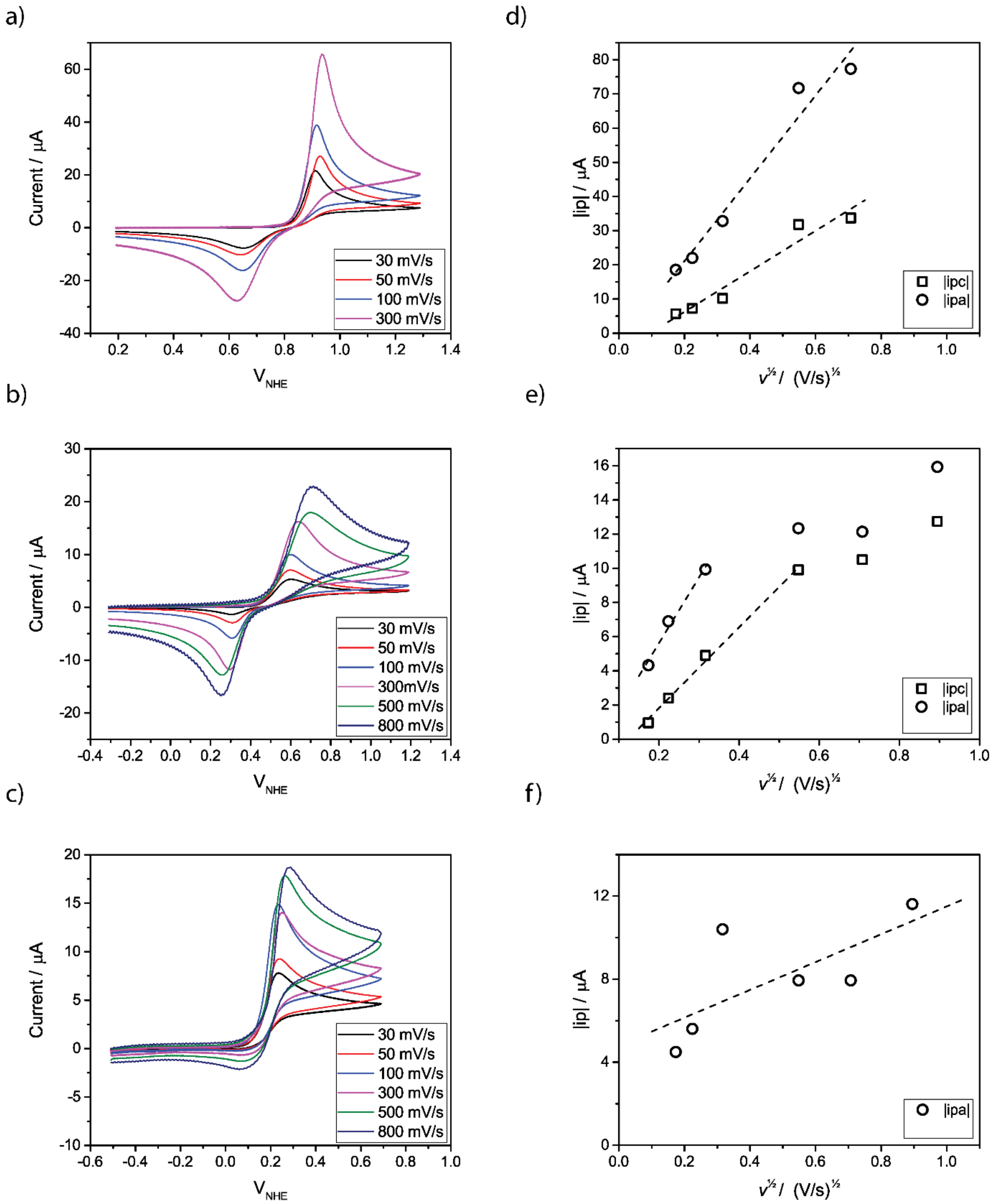

Figure 26: CV and ip vs. v plot for HQ(1,2) a)+d) pH 0, b)+e) pH 7 and c)+f) pH 13.

HQ(1,4): Para hydroquinone

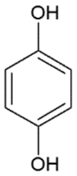

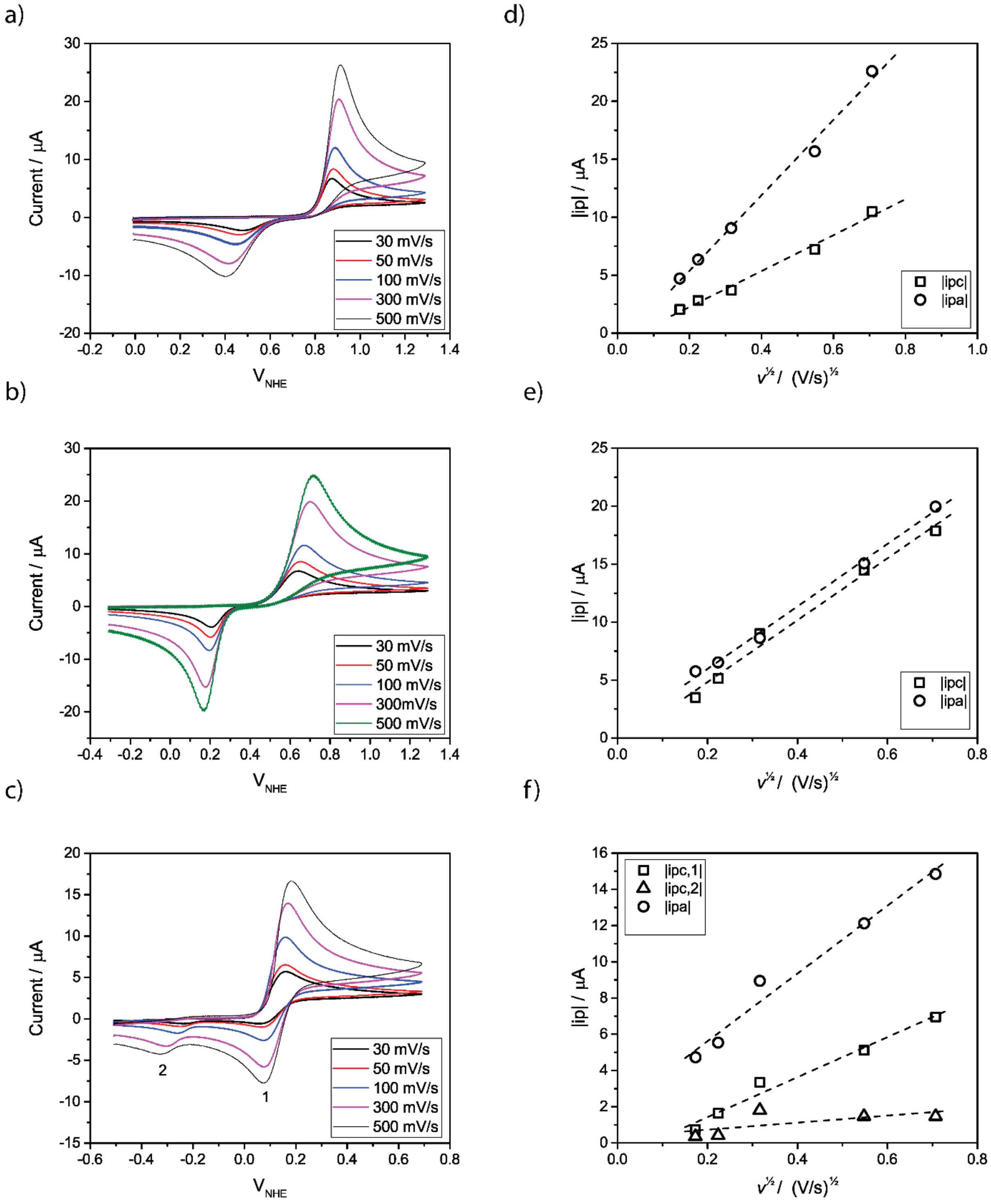

Figure 27: CV and ip vs. v plot for para hydroquinone a)+d) pH 0, b)+e) pH 7 and c)+f) pH 13.

HQ(1,2)DS (Tiron): 4,5-dihydroxy-1,3-benzenedisulfonate disodium monohydrate

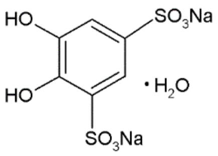

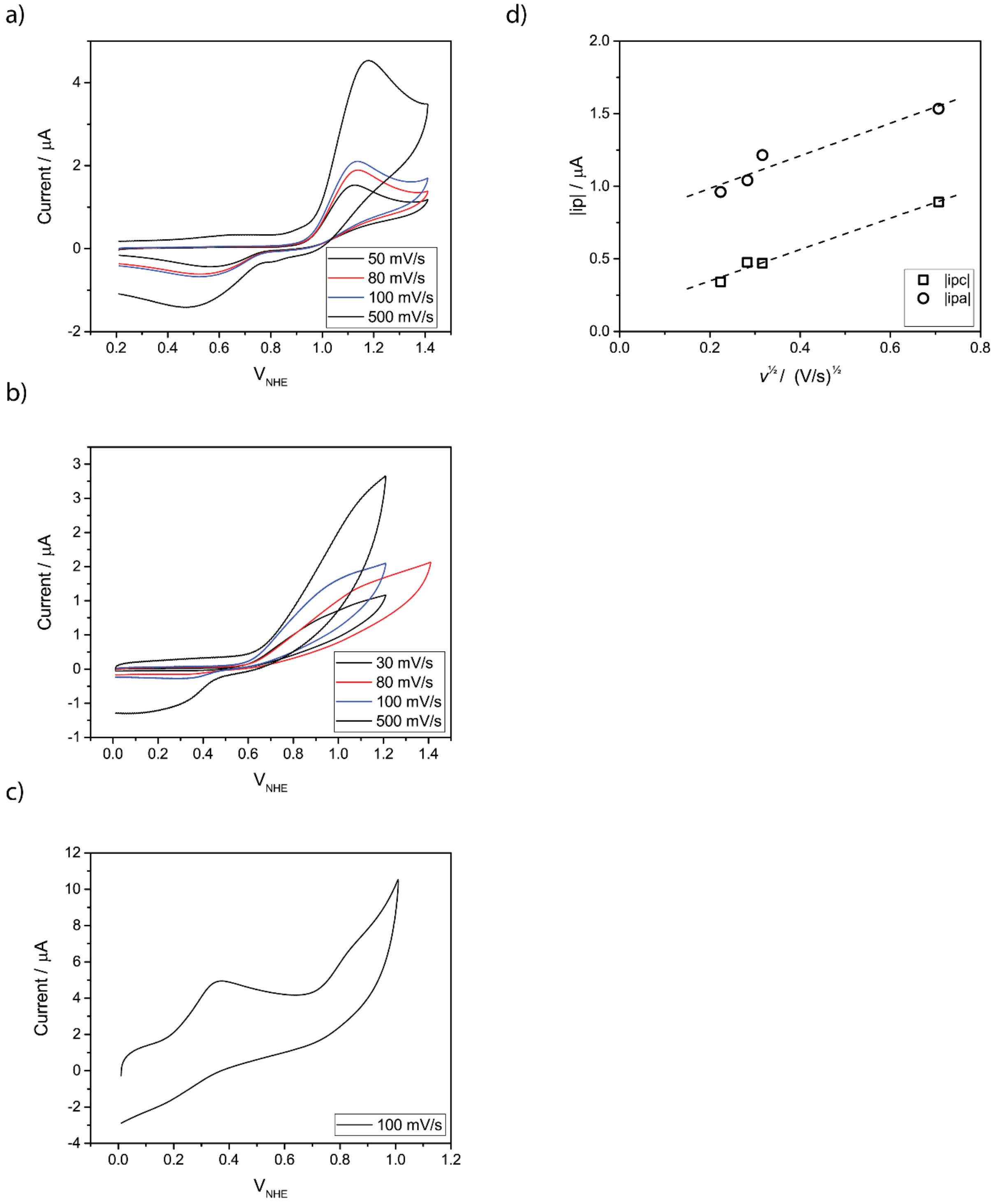

Figure 28: CV and ip vs. v plot for HQ(1,2)DS a)+d) pH 0, b) pH 7 and c) pH 13.

HQ(1,4)DH: 2,5-dihydroxy-1,4-benzoquinone

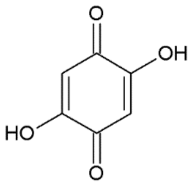

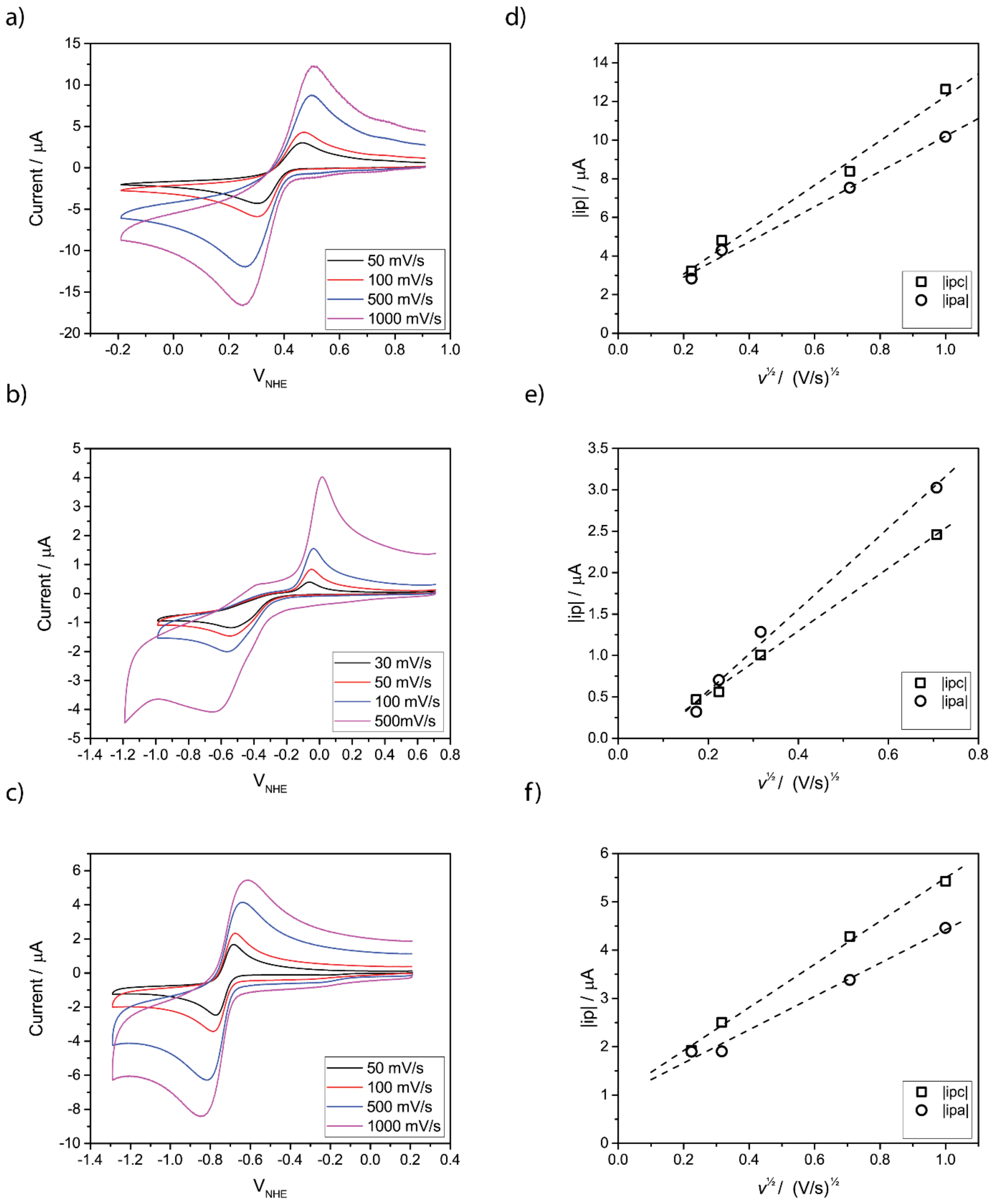

Figure 29: CV and ip vs. v plot for HQ(1,4)DH a)+d) pH 0, b)+e) pH 7 and c)+f) pH 13.

BQ(1,4)DHDCl (Chloranilic acid): 2,5-dichloro-3,6-1,4-benzoquinone

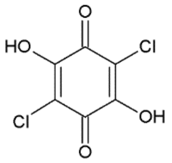

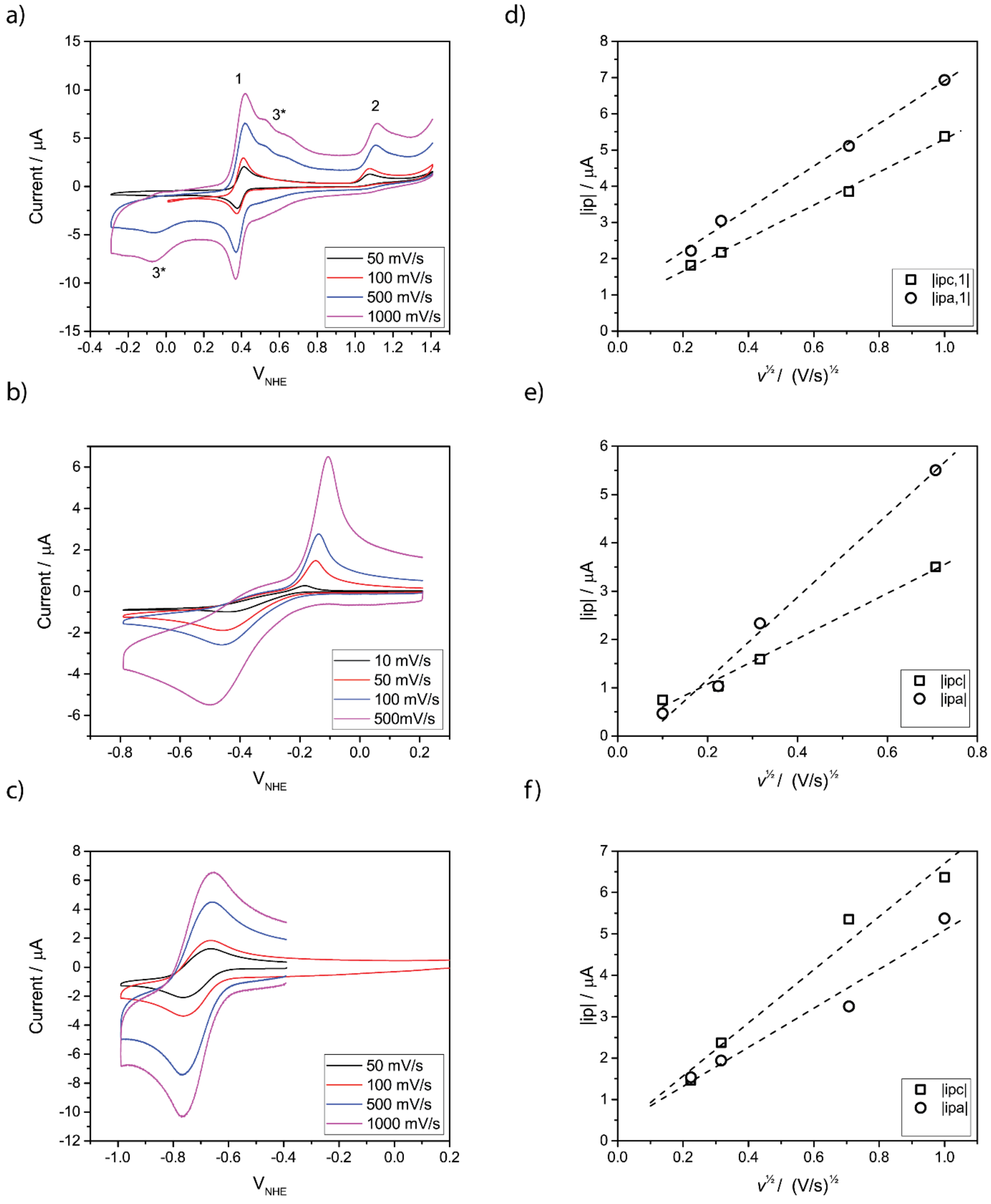

Figure 30: CV and ip vs. v plot for BQ(1,4)DHDCl a)+d) pH 0, b)+e) pH 7 and c)+f) pH 13.

HQ(1,4)TCl: Tetrachloro hydroquinone

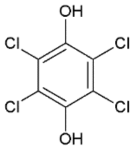

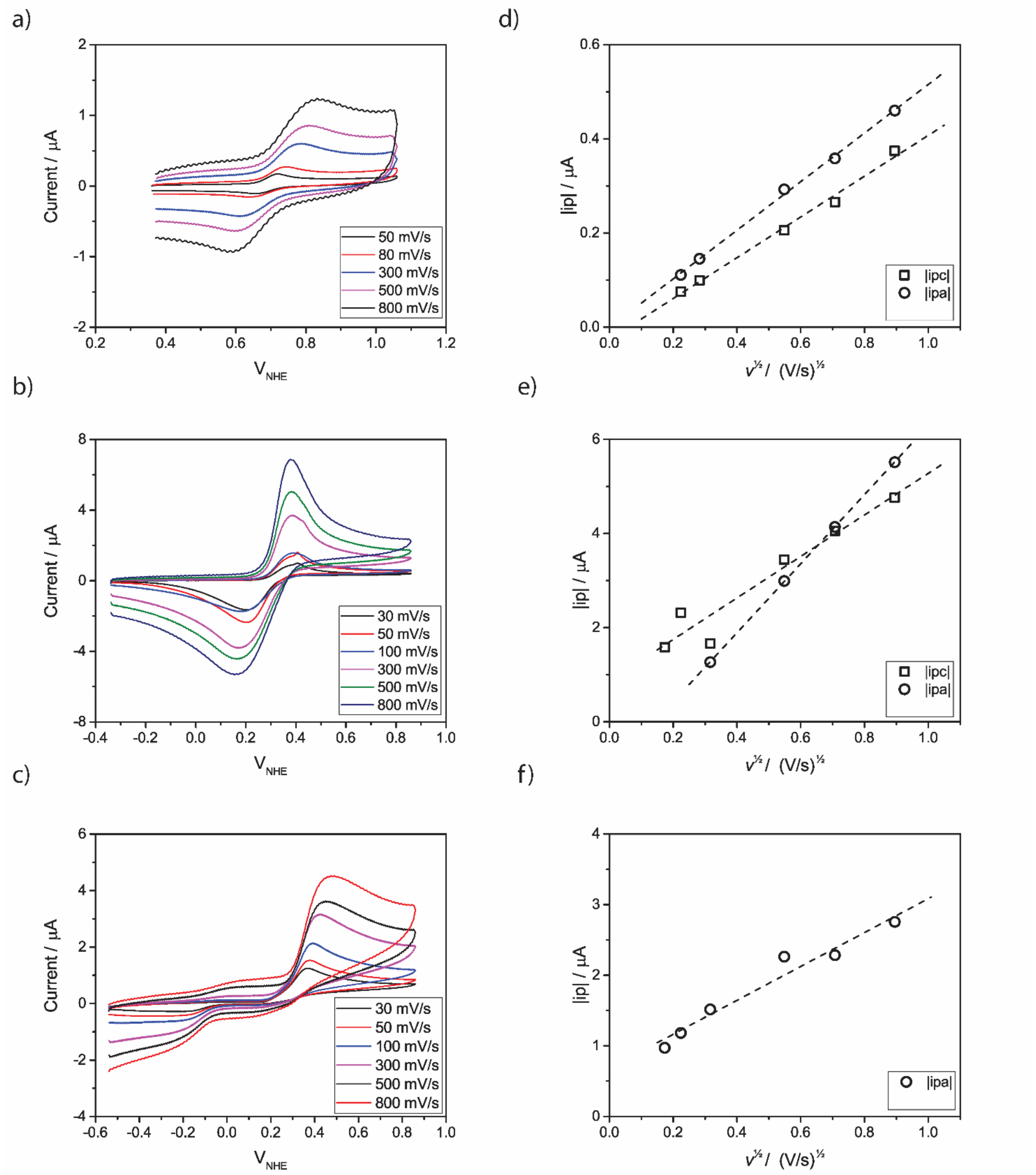

Figure 31: CV and ip vs. v plot for HQ(1,4)TCl a)+d) pH 0, b)+e) pH 7 and c)+f) pH 13.

BQ(1,4)TH: Tetrahydroxy-1,4-benzoquinone

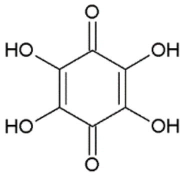

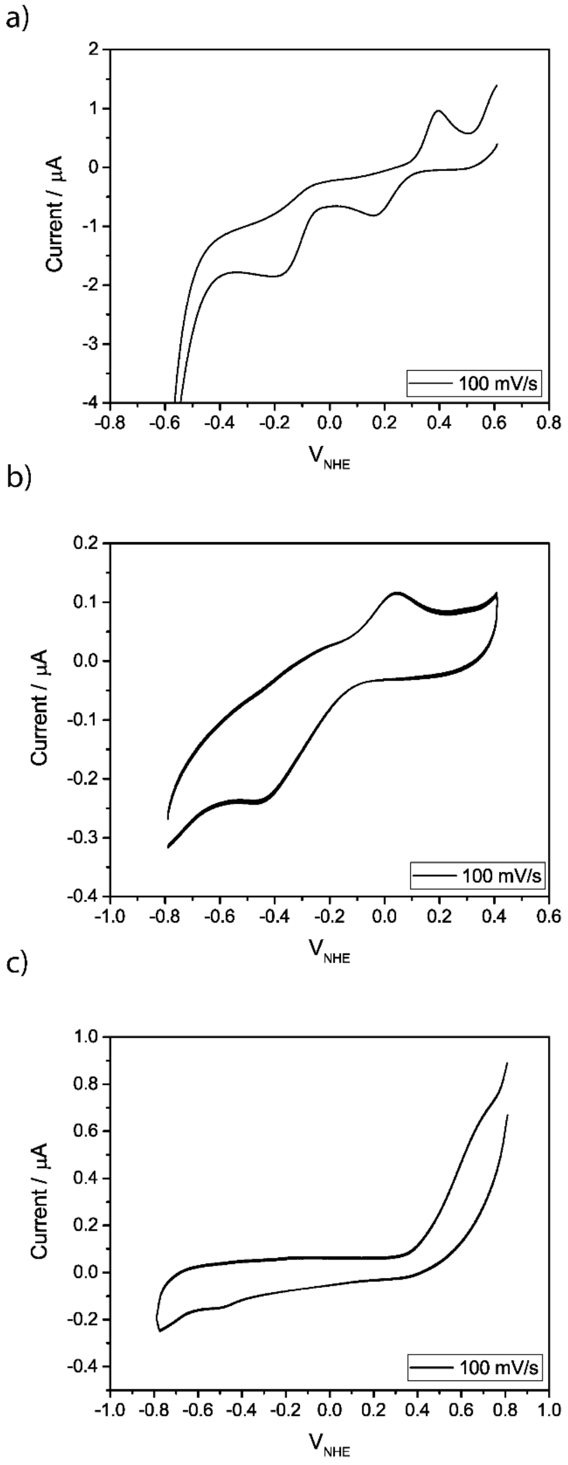

Figure 32: CV for BQ(1,4)TH a) pH 0, b) pH 6.2 and c) pH 13.

HQ(1,4)TF: 1,2,4,5-tetrafluoro-3,6-dihydroxybenzene

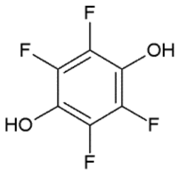

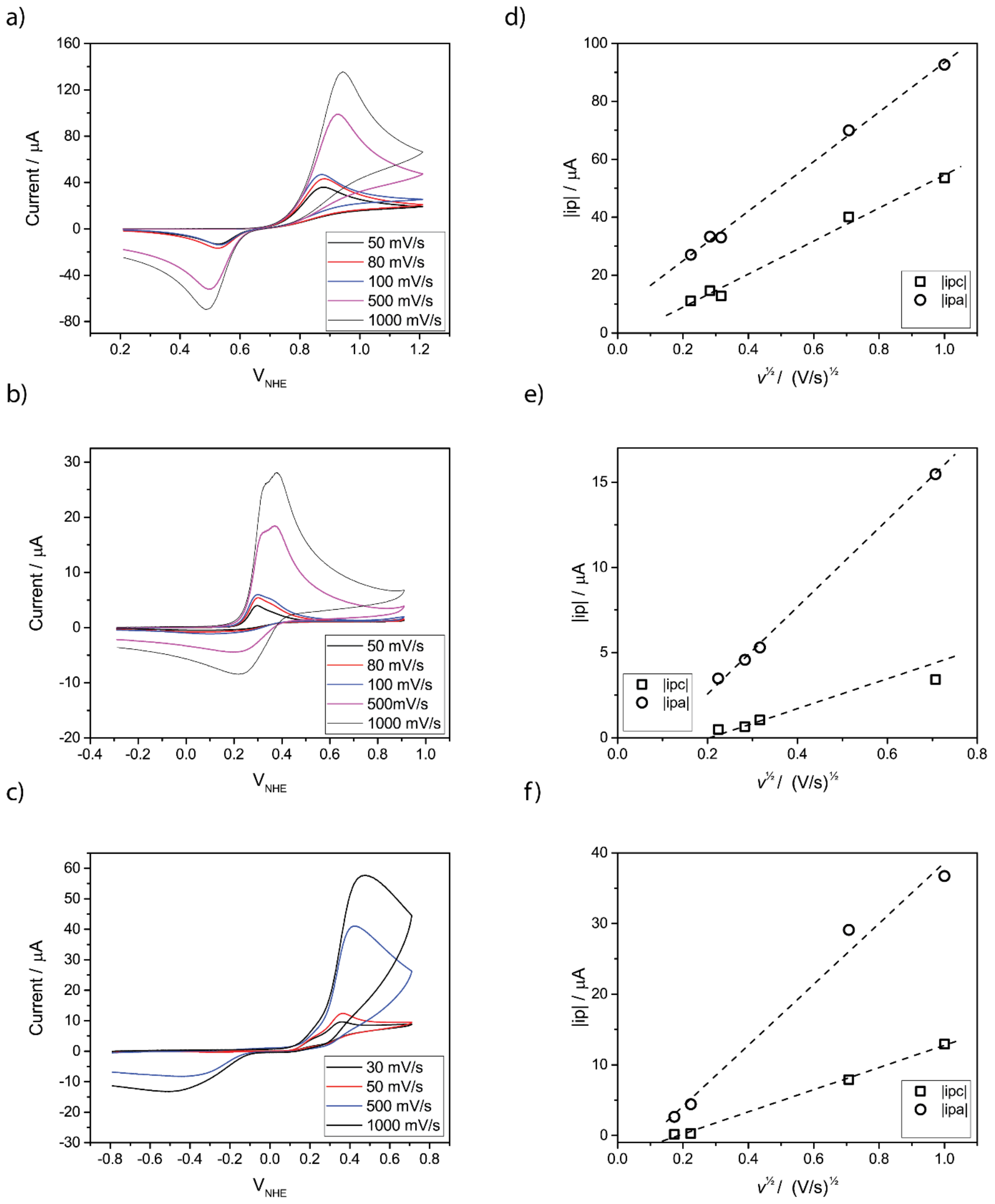

Figure 33: CV and ip vs. v plot for HQ(1,4)TF a)+d) pH 0, b)+e) pH 7 and c)+f) pH 13.

Pyrazine: 1,4-diazine

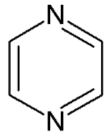

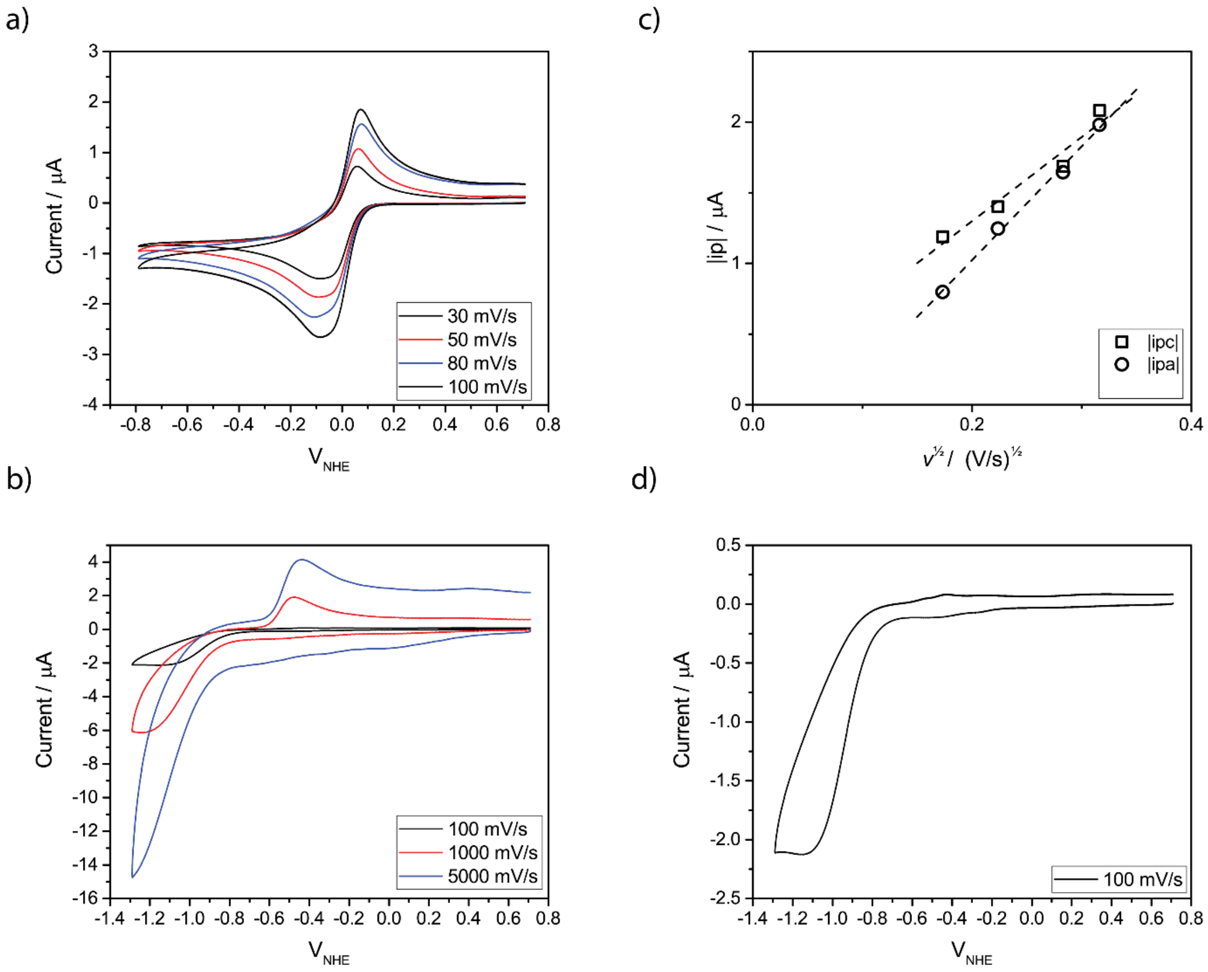

Figure 34: CV for pyrazine a)+c) pH 0 and ip vs. v plot b) pH 7 and d) pH 7 100 mV/s zoom.

Pyrazine-2-carboxylic acid

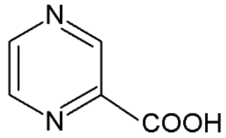

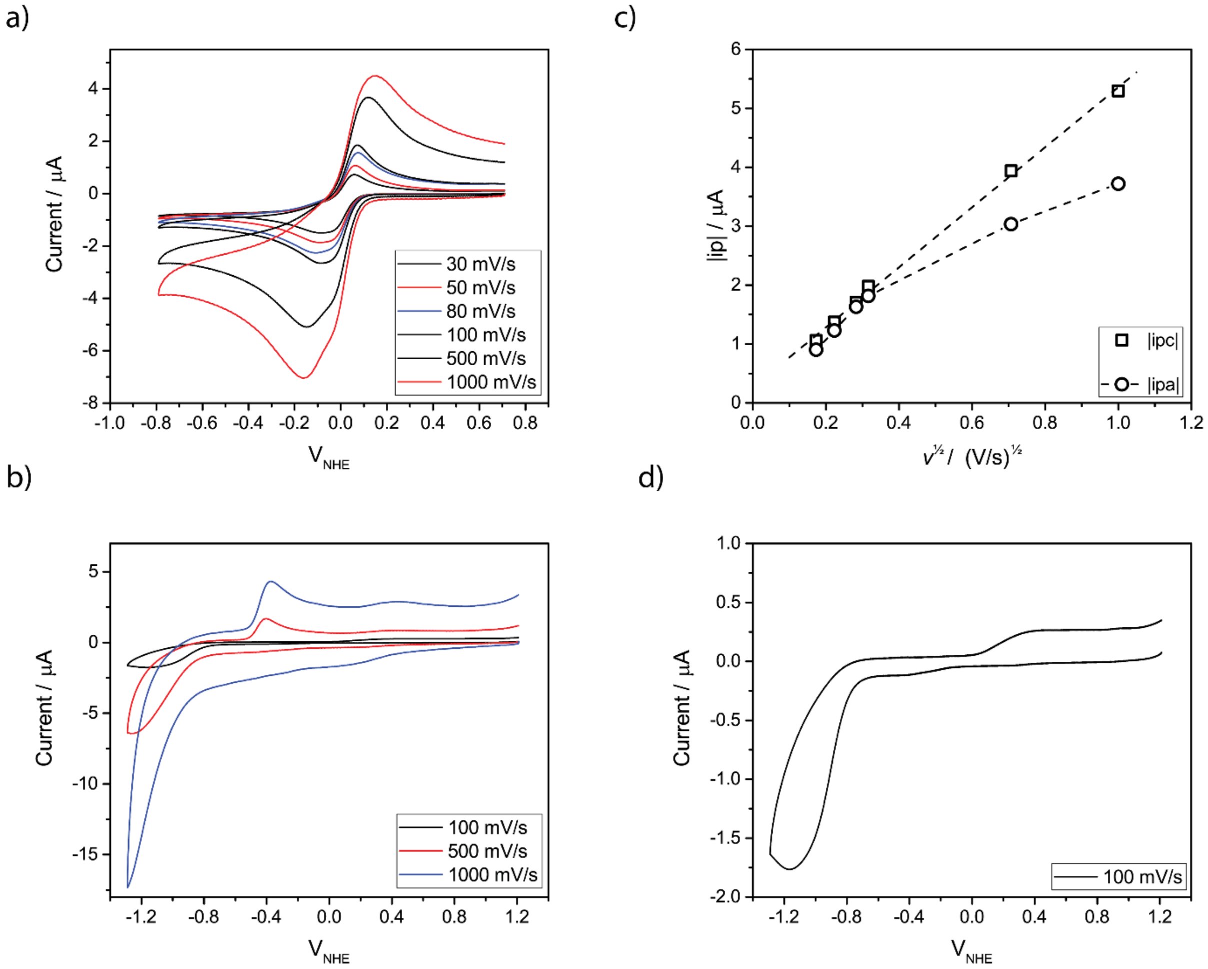

Figure 35: CV for pyrazine carboxylic acid a)+c) pH 0 and ip vs. v plot , b) pH 7 and d) pH 7 100 mV/s zoom.

Phenazine

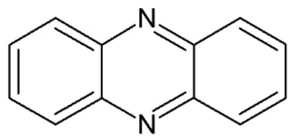

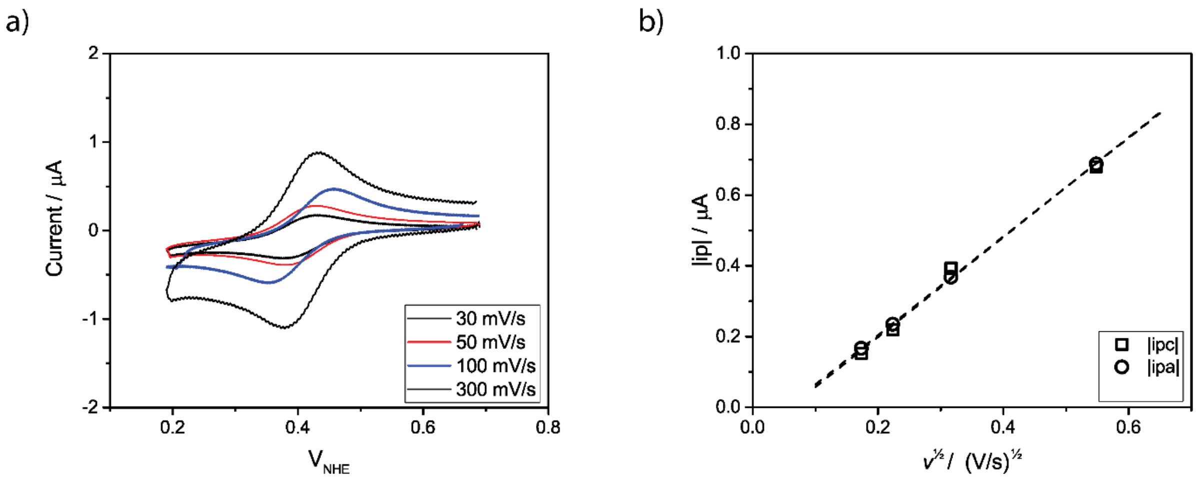

Figure 36: CV and ip plot for phenazine a)+b) pH 0.

TEMPOL: 4-hydroxy-2,2,6,6-tetramethylpiperidine-1-oxyl

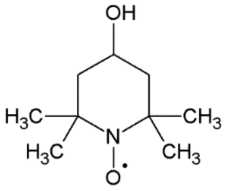

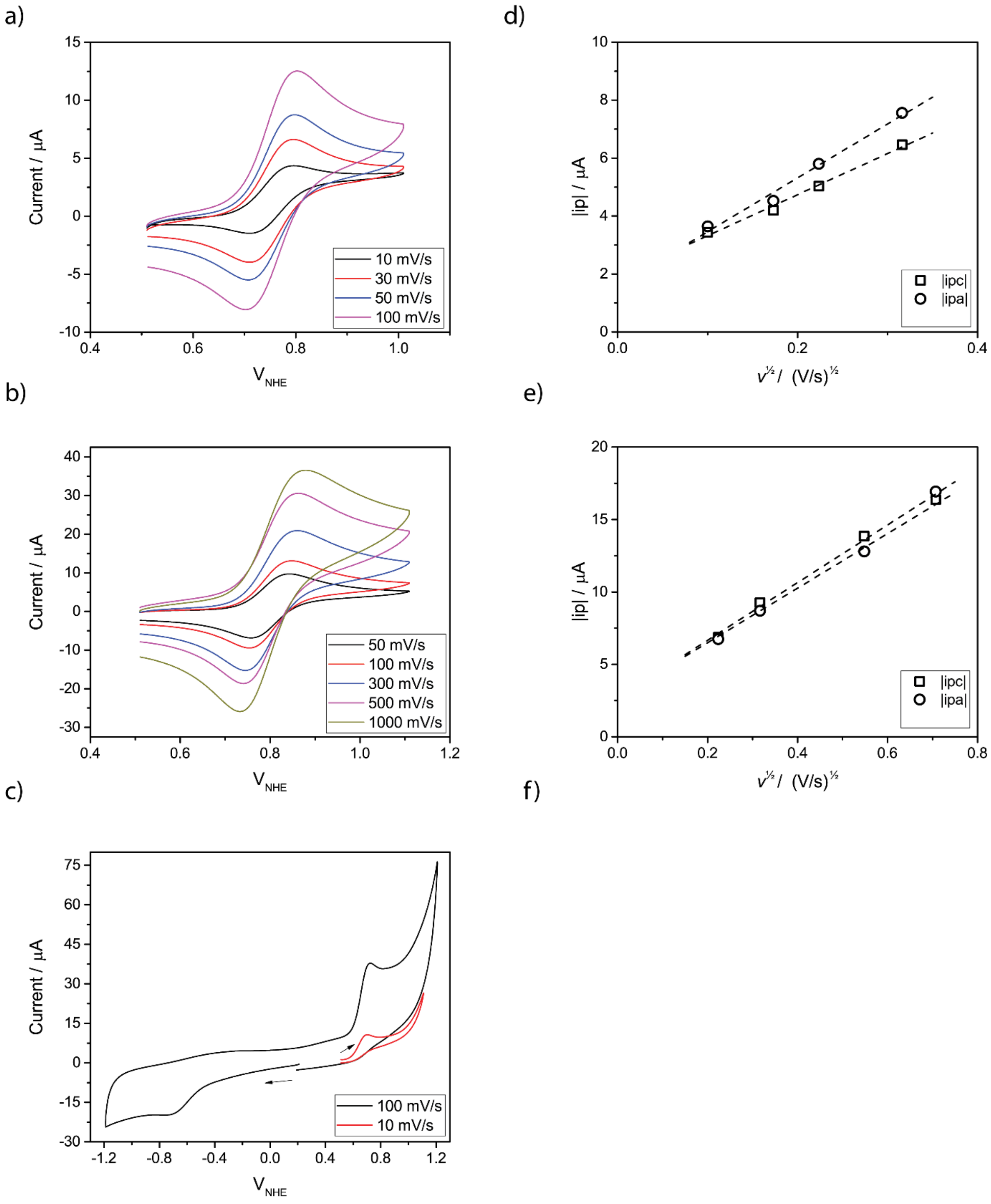

Figure 37: CV and ip vs. v plot for TEMPOL a)+d) pH 0 (oxidation), b)+e) pH 7 (oxidation) and c) CV pH 13 (oxidation and reduction).

S7: TEMPOL stability

Stock solutions of 1 M TEMPOL (reduced/uncharged state) in 1 M H_2SO_4 and 1 M KCl was prepared, purged with argon and hereafter kept oxygen-free in stoppered flasks. At different days, small volumes were taken out with the use of a needle and the stock solution dissolved in supporting electrolyte (1 m H_2SO_4 and 1 M KCl, respectively) to 50 mM concentration for CV evaluation at a GC electrode.

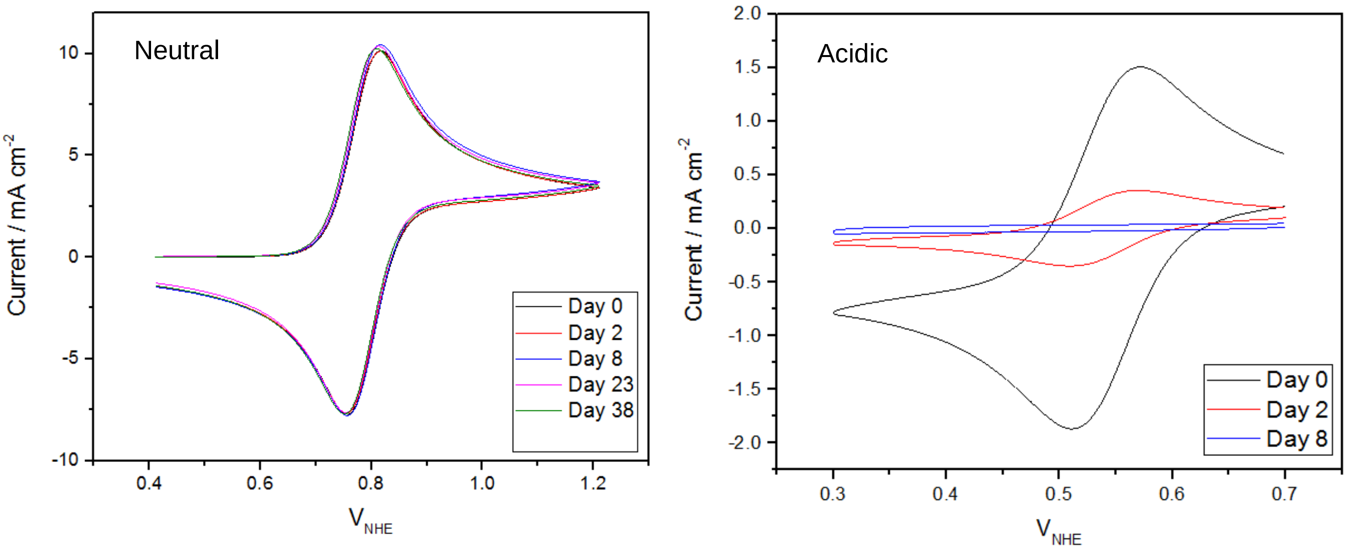

Figure 38: Overlay of CVs at different days for the TEMPOL solutions at 100 mV/s.

S8: Examples of side reactions

Sulfonyl substitution with nucleophiles

a.) OH^- as nuclefile[1]:
b.) OPh^- as nucleofile[2]:

a`)

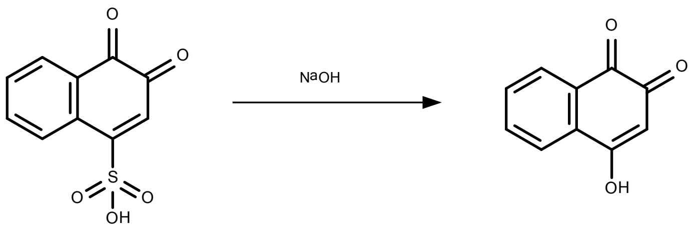

b`)

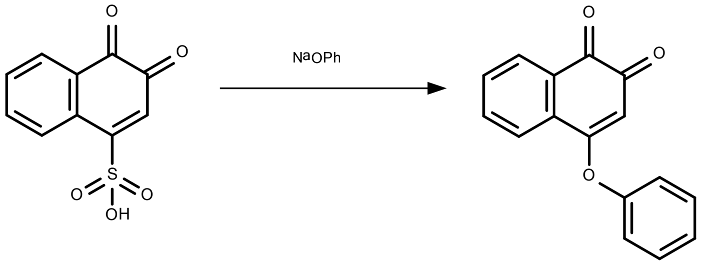

Substitution can happen in the whole pH range. OH^- is a far better nucleofile than water (H_2O), so the reaction is more predominant under alkaline conditions. However, because the product of $[H^+]$ and $[OH^-]$ is constant at 10^{-14}, at acidic pH the activity of OH^- falls from the point of view of nucleophilicity, but then water will act as nucleophile, since the water concentration is always high at about 55 M. Even if $[OH^-]$ is 14 orders of magnitude less, it does not mean that the rate of reaction will be 14 orders of magnitude lower. But it means that the substitution occurs faster in alkaline media than in acidic/neutral.

There are examples of sulfonic acid substitution even on aromatic ring and not on naphtoquinones. The reaction goes better if the system is poorer in electrons. For example in case of nitro, carbonyl and sulphonyl substituted benzene, the sulfonic acid will leave, even at 25 °C[3]

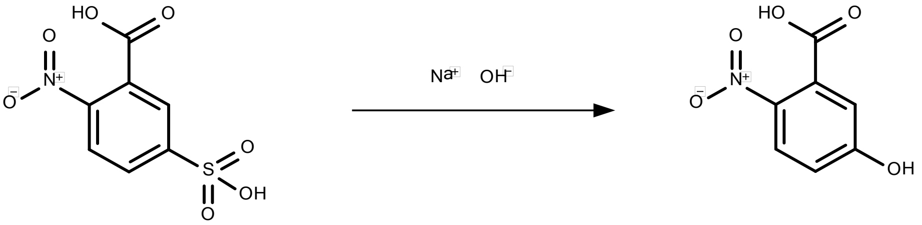

[1] J. Am. Chem. Soc., 1926, 48 (4), pp 1097–1107
[2] Bioorganic & Medicinal Chemistry 21 (2013) 523–531.
[3] J. Am. Chem. Soc., 1980, 102 (21), pp 6586–6587

c.) Disproportion reaction of anthraquinone in acidic media[4]

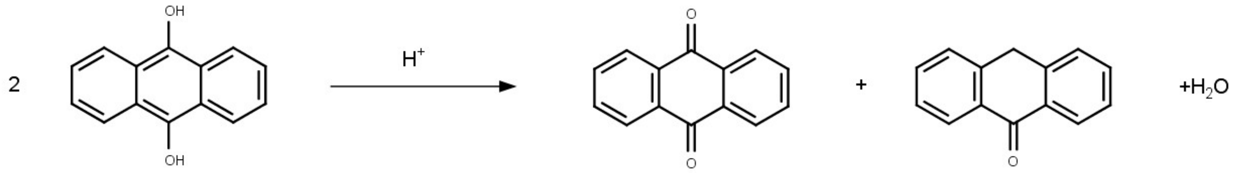

[4] Denki Kagaku, 1994 (62), p1202-1205

S9: Cost estimation of organic redox species

Bulk cost of Benzene is taken from www.icis.com/chemicals/channel-info-chemicals-a-z.

With respect to anthraquinone, it is somewhat more difficult to estimate the bulk cost. However, from www.cybex.in it is possible to get an overview of imported chemicals (to India) and the associated value. On www.cybex.in/imports-data-india/anthraquinone-hs-29146100.aspx (July 15, 2016) three different listings (> 10 MTS) were found. Here the average price was about \$2.9 kg^{-1}.

On www.alibaba.com Anthraquinone is found in the range from about \$2 kg^{-1} to \$4 kg^{-1}.

From these sources it appears to be safe to assume a cost of about \$3.0 kg^{-1} which corresponds to \$11.6 kAh^{-1} and in the main article the range \$10-15 kAh^{-1} is used.
